# A Survey of 802.15.4 TSCH Schedulers for a Standardized Industrial Internet of Things

**DOI:** 10.3390/s22010015

**Published:** 2021-12-21

**Authors:** Andreas Ramstad Urke, Øivind Kure, Knut Øvsthus

**Affiliations:** 1 Faculty of Information Technology and Electrical Engineering, Norwegian University of Science and Technology, Postboks 8900, 7491 Trondheim, Norway; 2 Faculty of Mathematics and Natural Science, University of Oslo, Postboks 1072, 0316 Oslo, Norway; knut.ovsthus@hvl.no; 3 Department of Computer Science, Electrical Engineering and Mathematical Sciences, Western Norway University of Applied Sciences, Postboks 7030, 5020 Bergen, Norway; oivind.kure@its.uio.no

**Keywords:** Time Slotted Channel Hopping (TSCH), scheduling, Industrial Internet of Things (IIoT), 6TiSCH, DetNet, cyber-physical systems

## Abstract

Concepts such as Industry 4.0 and Cyber-Physical Systems may bring forward a new industrial revolution. These concepts require extensive connectivity far beyond what is provided by traditional industrial networks. The Industrial Internet of Things (IIoT) bridges this gap by employing wireless connectivity and IP networking. In order for wireless networks to meet the strict requirements of the industrial domain, the Time Slotted Channel Hopping (TSCH) MAC is often employed. The properties of a TSCH network are defined by the schedule, which dictates transmission opportunities for all nodes. We survey the literature for these schedulers, describe and organize them according to their operation: Centralized, Collaborative, Autonomous, Hybrid, and Static. For each category and the field as a whole, we provide a holistic view and describe historical trends, highlight key developments, and identify trends, such as the attention towards autonomous mechanisms. Each of the 76 schedulers is analyzed into their common components to allow for comparison between schedulers and a deeper understanding of functionality and key properties. This reveals trends such as increasing complexity and the utilization of centralized principles in several collaborative schedulers. Further, each scheduler is evaluated qualitatively to identify its objectives. Altogether this allows us to point out challenges in existing work and identify areas for future research, including fault tolerance, scalability, non-convergecast traffic patterns, and hybrid scheduling strategies.

## 1. Introduction

A transformation is ongoing in industry where information technology (IT) is being integrated with operational technology (OT). Through concepts such as Industry 4.0, this is expected to significantly increase productivity and open for new applications [1,2]. The Industrial Internet of Things (IIoT) is crucial in this transformation since it enables massive collection of data, and process control without a wired infrastructure put into place. The wireless connectivity is therefore a critical component of IIoT. Although existing sensor networks employ wireless communication, they were developed for different requirements than those posed by industrial systems. This has warranted significant research efforts to develop wireless communication to match the performance seen in industrial cabled networks for example in reliability, deterministic latency, and IP interoperability.

Efforts to meet these challenges have been significant in the standardization bodies: The IETF Deterministic Networking (DetNet) working group https://datatracker.ietf.org/wg/detnet (accessed on 12 December 2021) aims at providing the upper layers with deterministic *flows*. These operate across multiple links- and network segments, and provide bounded latency, jitter, and loss. Flows may include wireless links, which is the focus of the Reliable and Available Wireless (RAW) working group https://datatracker.ietf.org/wg/detnet (accessed on 12 December 2021). The DetNet working group cooperates with its link-layer equivalent IEEE 802.1 Time-Sensitive Networking (TSN) task group https://ieee802.org/1/pages/tsn.html (accessed on 12 December 2021), which similarly aim for deterministic service over IEEE 802 networks. Lastly, there also exist full or partial network stacks for the industrial wireless domain such as WirelessHART [3], ISA100.11a [4], and WIA-PA [5], which constitute the most popular standards for industrial wireless sensor networks [6].

Making wireless communication a viable option to cabled networks requires research on all layers of networking. For the Media Access Control (MAC) layer, a popular approach relies on a combination of time-slotted access and slow channel hopping: Time Slotted Channel Hopping (TSCH). It offers potential for bounded delay and robustness against interference.

TSCH was added to the IEEE 802.15.4 [7] standard in 2016 (First introduced in the 2012 802.15.4e amendment [8]) and was based on the implementations found in WirelessHART and ISA100.11a. With TSCH, all nodes communicate according to a *schedule* that dictates the access to each cell, i.e., who may transmit and receive at a particular time and channel. Schedules may be built in a centralized fashion, such as done by the Network Manager in WirelessHART. This strategy allows for increased operator control and reservation of resources across the network in a holistic manner. However, decentralized approaches such as collaborative or autonomously built schedules are also feasible and typically allow for increased adaptability and fault tolerance. These different opportunities are giving rise to a significant research effort in the area of TSCH scheduling.

Our scope is limited to schedulers proposed for the 802.15.4 TSCH MAC with IIoT in mind. The scheduling of resources fundamentally impacts important networking metrics such as duty cycle, packet delivery ratio, end-to-end delay, network capacity, etc. These which are all key to fulfilling the requirements posed by industrial networks.

Existing surveys targeting TSCH scheduling are found in [9,10]. In addition to providing an up-to-date view in an area of rapid development, our survey differs in several ways: We employ a bottom-up approach when analyzing schedulers, where each scheduler is broken into basic components. This allows for a more granular insight as opposed to when viewed in terms of its goal or type of mechanism employed. We also include a survey of industrial requirements for the MAC layer and qualitatively evaluate the schedulers against these. It allows for an understanding of research effort focus and identifies open areas. Lastly, our survey is complemented by a holistic view of the field and each class of schedulers, where we identify and discuss seminal works, patterns, and trends.

The contribution of this survey is as follows:

- An up-to-date overview and classification of schedulers for the TSCH MAC approach;
- A holistic view on the field of TSCH scheduling, describing its evolution, key contributions and highlights, and current trends;
- Enhanced insight into TSCH scheduling through novel bottom-up analysis and qualitative objective evaluation;
- Identification of areas open for future research such as fault tolerance and heterogeneous traffic patterns, as well as challenges in existing research, including unrealistic assumptions and lack of repeatability;
- Description and evaluation of 76 surveyed TSCH schedulers available as supplementary material;
- Proposed improvements to the established TSCH scheduling taxonomy;
- Survey of industrial requirements posed on MAC layer from IIoT.

The rest of this survey is organized as follows: Section 2 describes the requirements of IIoT and their relations to the MAC layer. It also presents specific requirements identified for selected applications. Next, we provide a primer on TSCH, including the channel hopping mechanism and its rationale, before describing the 6TiSCH suite, which ties TSCH together with IPv6. Altogether this provides the necessary context for Section 5 and onward, where we survey and evaluate the state-of-art of TSCH schedulers according to our taxonomy and criteria. We start by describing the evaluation method used in the survey, summarize key contributions, and detail the evolution of the collective research effort. Each following section addresses one category of the schedulers and presents trends, features, and challenges. We conclude by describing areas open for future research and the challenges observed in existing work.

## 2. Requirements and Applications

Requirements on OT networks differ from that of IT networks. Generally, they are stricter and require more challenging bounds on metrics such as packet loss, delay, jitter, etc. Following is a description of these requirements and how they relate to the MAC layer.

- *Deterministic latency:* Industrial communication typically requires messages to be delivered within an upper bound on latency, and with as minor fluctuations in this latency as possible, i.e., minimal jitter. When a network is part of a control loop, this may be critical for the process to operate correctly. The network’s capability to provide such guarantees is also coined as its determinism [11], as well as its predictability [12]. Together with reliability, deterministic latency is widely considered the fundamental requirement in industrial communication [11,13,14,15,16,17,18,19,20].
- *Short latency:* Several industrial applications require the network to provide short delays on communication, i.e., the time needed for a message to reach its destination [11,21]. This is especially true for, e.g., closed-loop control applications where requirements can be in the microseconds range, as illustrated in Table 1.
- *Reliability:* Reliability relates to the network’s ability to transfer data successfully between sender and receiver, and it is typically measured using end-to-end packet delivery ratio (PDR). The MAC layer should aim to keep the frame loss at a minimum, described as “link-reliability” in [21]. Losses may incur retransmission mechanisms at MAC or higher layers, increasing latency and decreasing effective throughput. Industrial environments are especially challenging with difficult channel conditions due to co-existing networks, metal surfaces, industrial equipment, etc., [22,23].
- *Fault tolerance:* Industrial environments are harsh, both in terms of physical conditions such as dust, dirt, humidity, vibrations, and complex RF environments. The network must cope with challenging situations, such as, e.g., link, node, or gateway failures [24,25]. The MAC protocol must respond appropriately and provide the routing layer with available links to route over [26].
- *Scalability:* A large number of devices (hundreds or even thousands) are expected to be present due to their low cost, ease of deployment, increased industrial process complexity, and the requirements from realizing a Cyber-Physical System (CPS) [19,27,28,29].
- *Heterogeneous traffic:* An industrial network is not a static homogeneous entity, especially in the CPS paradigm. It typically consists of heterogeneous applications with differing requirements operating simultaneously, e.g., periodic process monitoring and an emergency action application. In addition, their corresponding requirements may change with time, e.g., monitoring processes that move between a transient- and a steady-state [30].
- *Throughput:* Especially in automation applications, a certain throughput is needed to meet the requirements of the industrial control loop. This is influenced by the payload size (which is typically small, below 100 bytes [13]) and the application control *cycle time*. In, e.g., closed-loop applications, the cycle time can be less than a millisecond (see Table 1), requiring more throughput [11].
- *Resource utilization*
  – *Energy:* Wireless nodes typically depend solely on batteries for energy. Each node is expected to last for at least several years and up to decades without requiring battery replacement or charging [31]. The MAC layer must keep the radio duty cycle and CPU utilization as low as possible.
  – *Bandwidth:* Given the scarcity of (especially unlicensed) frequencies, the available bandwidth for wireless networks is low and contested. The MAC should keep its overhead to a minimum, and minimize its band occupancy, i.e., utilize as few channels as possible to improve interoperability with co-existing technologies.
  – *Computational:* A wireless node may have less than 10 kB memory and 100 kB flash available [32]. Such hardware may not be able to accommodate complex and computational exhaustive MAC protocols. Requiring more capable hardware may increase the cost of each node, which may impede scalability.
- *Other:* A flexible and efficient *topology organization* is critical to accommodate, e.g., mobile nodes [18], or nodes which abruptly leave or join the topology [17]. As nodes are numerous and may be placed in hard-to-reach or dangerous locations, the network is required to be self-organizing and self-healing without manual human intervention [21]. *Co-existence and interoperability* between existing and new systems is a necessity in IIoT and CPS, where rich deployments are expected [17,31]. *Security* breaches in an industrial setting typically have more severe consequences than a traditional network and may lead to dangerous or even disastrous situations [29]. In addition to its role in hop-by-hop security, the MAC protocol often relies on signaling between devices or with a centralized unit, which must be secure [33]. A key benefit of wireless networking is reduced cost [34], and a MAC protocol should therefore offer an implementation, deployment, and maintainability which does not require significant capital- and operational expenditures.

**Table 1:** Example requirements for communication in the industrial automation domain [11,13,14,31,35].

Automation Domain	Class	Latency	Jitter	Packet Error Rate	Cycle Time	Range	Scale
Process	Monitoring	ms–s		>$10^{-9}$	s–days	10–100 m	50–1000 nodes
	Closed-loop control	ms		$10^{-9}$	<250 ms	10–100 m	<50
Factory	Closed-loop control	0.1–2.5 ms	1–20 μs	$10^{-9}$	0.5–5 ms	10–100 m	<50

Specific requirements are highly dependent on the industrial application and may also be settled by a holistic view on both application and network, as argued by Franchi et al. in [36]. The Industrial Society of Automation (ISA) https://isa.org (accessed on 12 December 2021) classify applications and their requirements into three categories:

- *Safety applications* such as emergency shutdowns are always critical and have the most stringent requirements.
- *Control applications* are diverse, and the requirements depend on whether the network serves a closed- or open-loop system. Table 1 showed example requirements for the automation domain, divided into (1) Process control/automation, e.g., oil, gas, and mining, and (2) Factory/manufacturing, which is typically assembly line productions such as in the automotive industry.
- *Monitoring applications* such as asset tracking and history collection are typically limited to gathering non-critical data at longer time-spans and thus have the laxest requirements.

More on the classification of industrial applications may be found in [21,37,38].

## 3. 802.15.4 Time Slotted Channel Hopping

**Figure 1:**
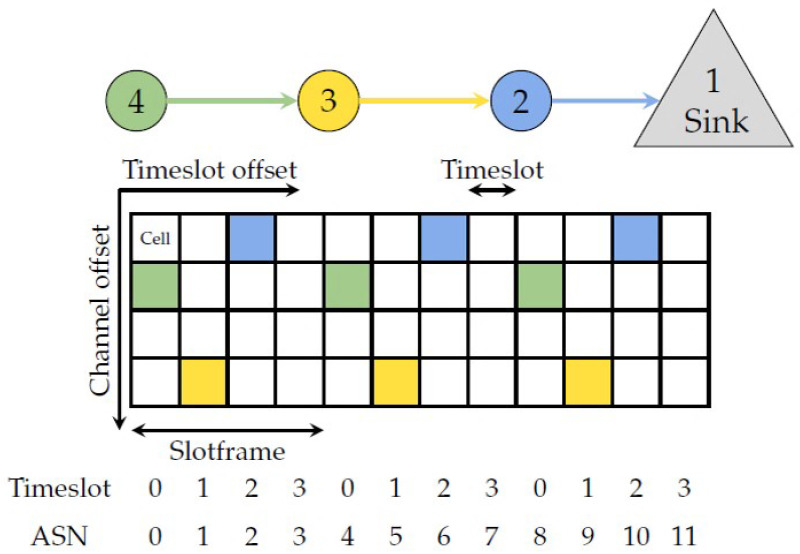
Simple wireless network topology with example TSCH schedule.

Figure 1 illustrates a simple topology with an accompanying TSCH schedule, where time is divided into timeslots horizontally and channels vertically. A specific *cell* allows transmitting one packet and an optional acknowledgment, and can be identified by its timeslot and channel offset. A cell can be shared between multiple nodes, e.g., for broadcasts, or it can be dedicated, which yields contention-free communication. This scheme allows for low energy consumption since devices sleep if the schedule does not dictate otherwise.

A collection of timeslots repeats in periods called *slotframes*, which in Figure 1 is 4 timeslots long. The 802.15.4 standard does not specify the schedule content or how it is built. This allows for application-specific solutions and opens up the research area of TSCH schedulers.

Coordinator nodes periodically broadcast Enhanced Beacons which contains the current *absolute slot number* (ASN). ASN is the total number of timeslots elapsed since the deployment of the network, as illustrated in Figure 1. The broadcasts ensure all nodes are synchronized and in agreement on which is the current timeslot in the slotframe.

This synchronization is also used to implement channel hopping: The industrial RF environment is challenging with metal surfaces and interference introduced by machinery, engines, welders, etc. This is further emphasized by co-existing technologies such as Wi-Fi [19,39]. Together this exacerbates external interference and multi-path fading, reducing the reliability of the communication [40]. These effects are frequency-dependent and may thus be combated by hopping to a different channel every time a cell is used. The ASN is used to identify the physical channel to use:

ChannelIndex=(ASN+ChannelOffset)modNumCh

where *ChannelIndex* identifies which channel in the hopping sequence list to use, and *NumCh* is the number of channels. Consequently, the same cell will use a different channel for each slotframe. The most popular 802.15.4 PHY in the 2.4 GHz band specifies 16 non-overlapping channels which may be utilized in the hopping list. In [41] it was shown this scheme may decrease the expected transmission count (ETX) by 56%, with additional improvements being possible by employing a blacklist such that the hopping scheme avoids the least optimal channels.

Whereas channel hopping combats the impact of frequency-dependent multi-path and interference, dedicated cells mitigate collisions. Combined, this allows for high reliability, low-power operations, and deterministic behavior. Interested readers may refer to [22] for additional details on 802.15.4 TSCH.

## 4. Combining TSCH and IPv6-6TiSCH

Realizing the visions of Cyber-Physical Systems and IIoT requires combining industrial networks, i.e., operational technology, with IP-based networks, i.e., informational technology. A crucial part of this is realizing a network protocol stack that combines the connectivity of IPv6 with an industry-capable MAC. In this section, we will describe relevant work from the 6TiSCH working group https://datatracker.ietf.org/wg/6tisch (accessed on 12 December 2021). This is important since many schedulers are designed for 6TiSCH networks and utilize included mechanisms such as the 6top Protocol (6P) [42]. A tutorial on 6TiSCH may be found in [43].

### 4.1. 6TiSCH Stack

**Figure 2:**
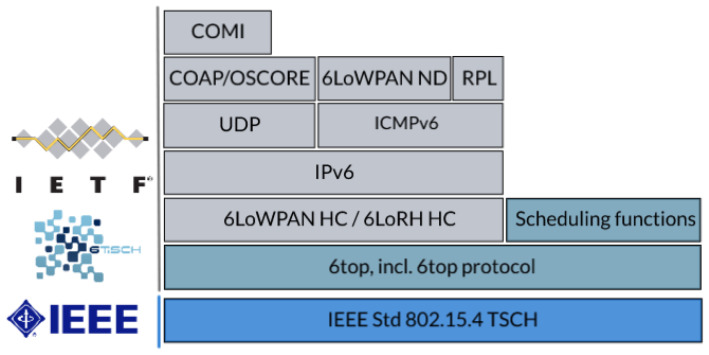
The 6TiSCH protocol stack.

Figure 2 depicts the proposed protocol stack for 6TiSCH networks. It consists of existing higher-layer IETF standards such as UDP, IPv6, RPL [44], and 6LoWPAN, on top of the IEEE 802.15.4 TSCH MAC. To integrate the two parts, a new *6top sublayer* [42] is specified in between. It provides an interface to TSCH resources such as the schedule and connectivity statistics, as well as defining how nodes should communicate scheduling requests to each other utilizing the *6top Protocol* (6P) [42]. The 6TiSCH protocol stack is implemented in at least four open-source embedded operating systems as per December 2021 [45]: OpenWSN (http://openwsn.org (accessed on 12 December 2021)), Contiki-NG (https://contiki-ng.org (accessed on 12 December 2021)), RIOT (https://riot-os.org (accessed on 12 December 2021)), and TinyOS (http://tinyos.net (accessed on 12 December 2021)).

**Figure 3:**
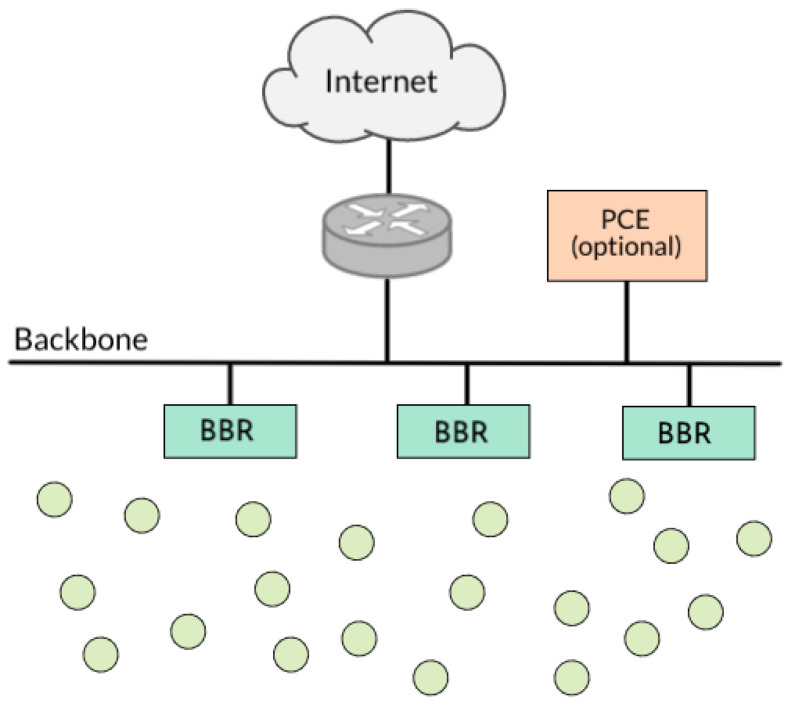
6TiSCH network with several backbone routers (BBR) and an optional Path Computation Entity (PCE).

A 6TiSCH network consists of one or more Low Power Lossy Networks (LLN) sharing an IPv6 subnet and running a TSCH-based mesh [46]. Figure 3 depicts this envisioned network. Inside the 6TiSCH LLN, each node runs the 6TiSCH stack and has an 802.15.4 TSCH capable radio. The Border Routers acts as a gateway between the LLN and the outside, performing additional duties such as 6LoWPAN termination and root role in the RPL routing tree.

### 4.2. 6TiSCH Scheduling

The Scheduling Function (SF) decides the content of a node’s schedule. SFs are interchangeable, offering operators the flexibility to employ whichever SF meets their requirements. The workgroup therefore has limited contributions on scheduling functions: 6TiSCH specifies a *minimal mode* simple static schedule [47] for network bootstrapping. Secondly, it specifies the Minimal Scheduling Function (MSF) [48] intended for generic use-cases.

6TiSCH describes four different scheduling approaches [46] to manage the TSCH schedule: *Remote monitoring and schedule management* realizes a centralized scheme, *Static* is a preset fixed schedule. *Neighbor-to-neighbor*, and *Hop-by-hop scheduling* are decentralized approaches. In hop-by-hop, a communication track is envisioned over several hops through the network, while neighbor-to-neighbor limits its scope to the node neighborhood.

The 6top sublayer offers ways for the SF to add, delete, count, etc., cells with a neighbor node. Any negotiations between nodes to achieve this is done by the 6P protocol described below. 6top also offers link statistics such as RSSI, time since last packets, number of packets, etc., which an SF can use to make scheduling decisions.

### 4.3. 6top Protocol

When an SF requires the 6top sublayer to change the schedules between two nodes, 6top utilizes the 6top Protocol (6P) [42] to communicate with its neighbor. Through exchange of *messages*, 6P allows for negotiation of scheduling modifications such as adding, deleting, relocating, and listing cells. These *transactions* typically follow a request-response pattern where one side initiates the operation and suggest the relevant cells, upon which the other side will respond with the result.

**Figure 4:**
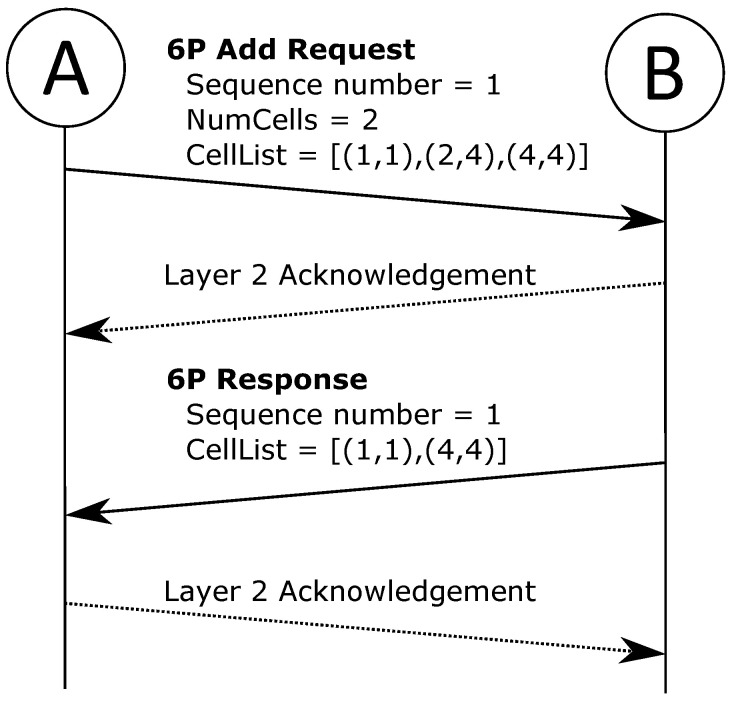
Simplified example of 6P Add transaction between node A and B.

To exemplify the operation, Figure 4 shows a 2-step adding-of-cell transaction. At node A, the SF has decided two cells are needed towards node B, and a 6P transaction is initiated. Node A transmits a 6P request message with the ADD command, accompanied by a list of candidate cells. Node B consults its SF and responds with a subset of acceptable cells—thus, the transaction is completed, and two new cells are scheduled between nodes A and B.

## 5. Survey of TSCH Schedulers

The scheduling function is the heart of a TSCH solution. The arrangement of reservations in time and frequency is the key decider to fulfill essential requirements such as reliability, latency, and energy consumption. Neither the 802.15.4- nor 6TiSCH-standard specify anything other than minimal schedules—leaving room for significant research into scheduling mechanisms. The following sections survey these efforts and the resulting schedulers.

### 5.1. Methodology

Schedulers were identified by searching Google Scholar (https://scholar.google.com (accessed on 12 December 2021)) with keywords “TSCH”, “Time Slotted Channel Hopping”, and “6TiSCH”, and limited to publishing before Jan. 1st 2021. From the set of matches, actual schedulers were identified qualitatively. Lastly, schedulers were disqualified if they did not address 802.15.4 TSCH or if the application was unrelated to the industrial domain.

### 5.2. Taxonomy

A common approach is to categorize schedulers according to the fashion a schedule is generated. This is especially useful because the generation of a schedule dictates or influences most of its properties. Our survey is therefore organized accordingly, and each category is presented in turn. This taxonomy is employed in several other works: Hermeto et al. [9] utilize only *Centralized* and *Distributed* categories, while 6TiSCH adds a *Static* class and divide distributed schedulers into hop-by-hop and neighbor-to-neighbor. Lastly in [10], the authors leave out the static class and employ *Centralized*, *Distributed*, *Autonomous* and *Hybrid* categories. Thus, based on our findings, no proposal captures the complete range of schedulers while providing the necessary level of detail.

**Figure 5:**
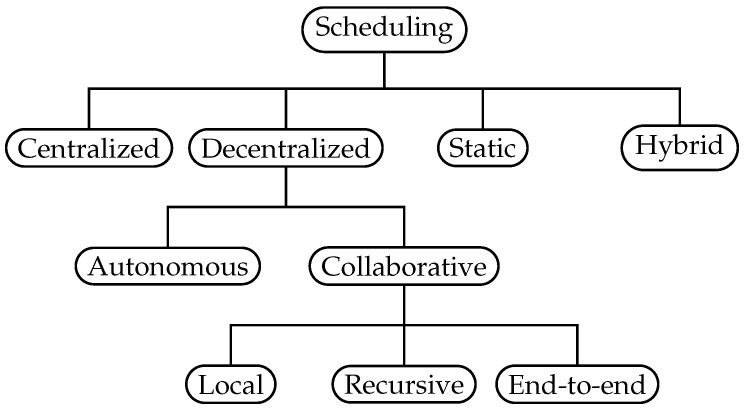
Classification of scheduling strategies according to schedule generation.

Based on existing schemes, we suggest an improved taxonomy in Figure 5 that encompasses all categories. Further, a *Collaborative* class replaces the distributed class. This is done to distinguish autonomous self-sustained approaches from the collaborative joint effort strategy. With collaborative scheduling, neighboring nodes schedule cells by negotiating or sharing dedicated information, e.g., via the 6P protocol or piggy-backing on data- or routing-packets. This as opposed to autonomous scheduling, where each node builds a schedule without any dedicated communication between neighbors or a central entity, denoted as “pure” autonomous scheduling in [49]. Lastly, we capture the crucial differences within the collaborative strategy by sub-dividing into three groups, *local*, *recursive* and *end-to-end*. This grouping reflects the differences in how reservations are made and the awareness of traffic requirements, as shown in Table 2. We distinguish neighbor-to-neighbor schedulers (coined in 6TiSCH, see Section 4.2) which operate only on local information such as queue sizes, from those aware of neighbors traffic requirements—allowing for greater insight. We favor the term *end-to-end* as opposed to *hop-by-hop* in 6TiSCH, as it clearly conveys how the reservations are managed between two peers.

Within the *local* group, schedules are based on local traffic requirements, and the scheduling protocol only operates across one hop. Nodes evaluate only their own local information such as queue size or cell utilization to decide the cell allocation. Consequently, to meet traffic requirements end-to-end, the local approach relies on each node to react on its queue length, cell utilization, etc.

Within the *recursive* group, the traffic requirements are known along a path, while reservations are made on a one-hop basis. Signaling the requirements are either done using a dedicated protocol or deduced from existing cell reservations. By combining this information with knowledge of the traffic generated at the node, a scheduler can calculate the accumulated requirements. This process operates recursively and depends on each node fulfilling and forwarding the requirements to ensure sufficient resource allocation.

Within the *end-to-end* group, both requirements and reservations are made in an end-to-end fashion. This is achieved by employing a multi-hop protocol such as the Resource Reservation Protocol—Traffic Engineering (RSVP-TE) [50] to disperse information and ensure end-to-end reservations. This also allows the originating node to be aware of the success or failure of the reserved path.

### 5.3. Timeline

**Figure 6:**
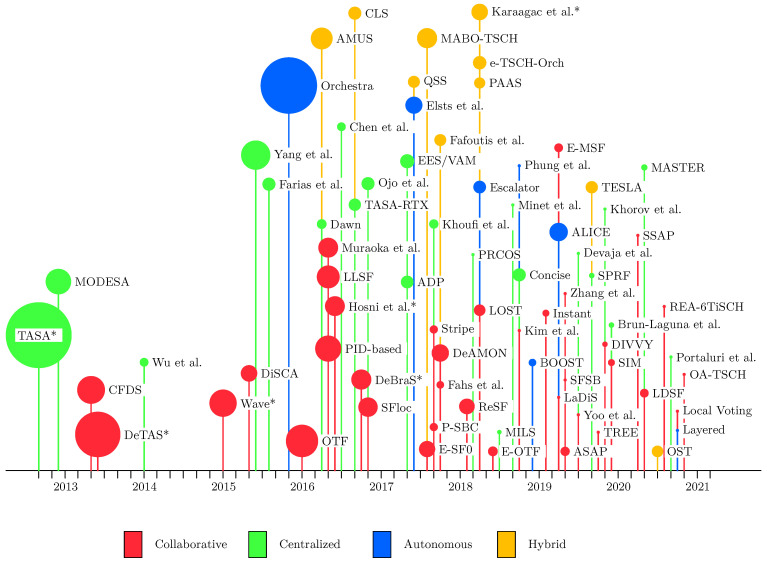
Timeline of schedulers. Size of bubble is relative to number of citations according to Google Scholar. * indicate multiple publications for same scheduler—earliest publication date is shown.

To show the evolution of TSCH schedulers, Figure 6 presents a timeline of the 76 different proposals reviewed in our survey. The bubble size indicates the number of citations—intended to give an impression of the impact of each scheduler. Seminal contributions become highlighted, such as TASA [51], DeTAS [52] and Orchestra [53], upon which many other schedulers expand or compare themselves against. It is also worth noting more recent proposals like LOST [54], DeAMON [55], ALICE [56], and MABO-TSCH [57] which have already garnered attention. Other schedulers of interest include OTF [16], which served as an early foundation for the 6TiSCH Minimal Scheduling Function (MSF). Surveying the more recent years showed that advances are now happening in increasingly specialized and smaller increments, such as the numerous enhancements to Orchestra. This as opposed to the larger leaps seen in the earlier years of TSCH scheduling research.

In some cases, a scheduler is presented across multiple publications. This is often done to expand the scheduler evaluation, e.g., adding testbed experiments as in DeTAS [58], or to expand or enhance the scheduler as done by Hosni et al. [59,60].

**Figure 7:**
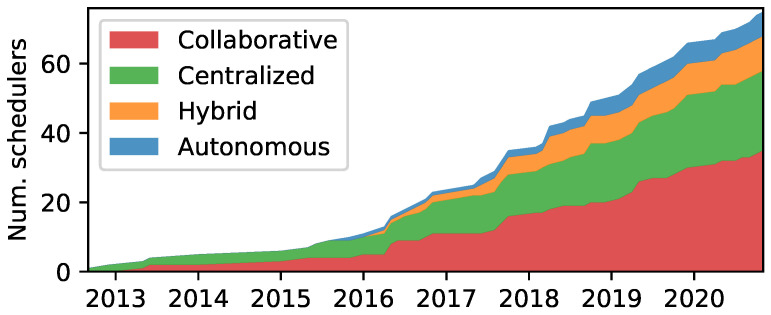
Cumulative distribution of schedulers per category.

After 2015 there has been a marked increase in new scheduler proposals, indicating a growing interest in the area, as depicted in Figure 7. Contributions are concentrated around centralized and collaborative approaches. However, recently, a slight decline in centralized proposals has been observed. Autonomous and hybrid schedulers were first proposed in 2015 and 2016, yet have lately gotten increased traction. Despite the limited number of schedulers, there are notable proposals in the autonomous category, especially Orchestra, as indicated in Figure 6. It should also be noted that four out of the ten hybrid schedulers employ autonomous mechanisms—which will be discussed in Section 9.

**Figure 8:**
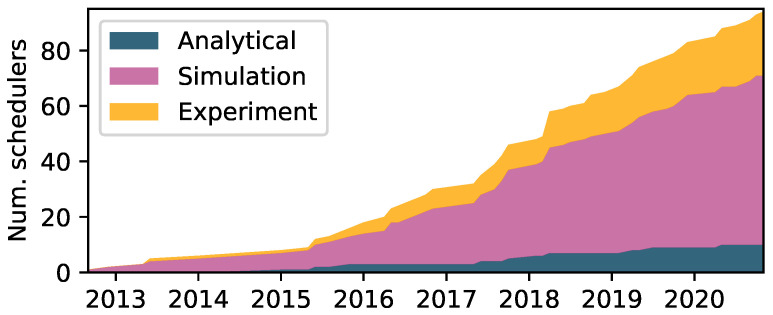
Cumulative distribution of schedulers per evaluation method.

Simulation is, and has always been, by far the most popular method for evaluating TSCH schedulers. Figure 8 depicts this trend. The most popular simulators include COOJA [61], 6TiSCH simulator [62] and OpenSim [63]. Surprisingly, experimental evaluation is still not commonplace, despite the increased availability of open testbeds such as FlockLab 2 [64] and FIT IoT-Lab [65]. However, later years have seen increased employment, perhaps spurred by initiatives such as the recently established annual Workshop on Benchmarking CPS and IoT [66], and related works towards a common framework [67] and methodology [68]. Real-world or testbed experiments are typically the final steps in evaluations due to their complexity and time-consuming setup. Thus the evaluation method(s) usually indicate a scheduler’s maturity, e.g., the influential proposals Orchestra, OTF, and DeTAS have all been evaluated in testbed setups. A survey on available testbeds and simulators may be found in [69]. Analytical modeling is rarely used and typically employed as a preliminary to, e.g., identify theoretical bounds.

**Figure 9:**
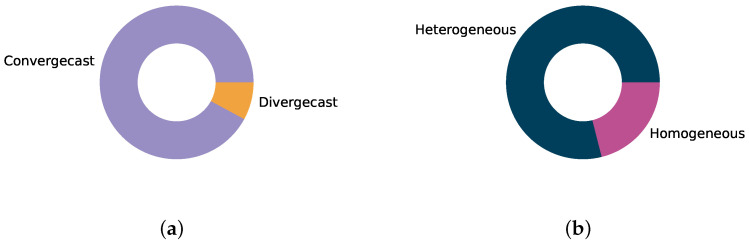
Distribution of evaluated traffic scenarios in schedulers. Divided into *patterns*, i.e., where traffic flows, and *profiles*, i.e., the intensity of each flow, as suggested by [69]. (**a**) Traffic patterns; (**b**) Traffic profiles.

The vast majority, 70 out of the 76 surveyed schedulers, target or evaluate a convergecast traffic pattern where all nodes transmit to one sink, typically located at or beyond the network root. Convergecast mimics the classical monitoring application where a logger or controller receives information from a range of sensors. Convergecast patterns yield a funneling effect where traffic intensity increases close to the sink. Schedulers must adjust for this, which may be challenging in, e.g., autonomous strategies where the uneven distribution of resources is difficult to implement. As depicted in Figure 9a, only six schedulers employ divergecast patterns in their evaluation. These include the autonomous schedulers Orchestra and ALICE, which consider IoT-like applications such as request-response transactions and firmware downloads. Thus there has been limited attention towards, e.g., sensor-to-actuator traffic patterns required in concepts such as Smart Manufacturing [70]. However, note that several collaborative and (especially) centralized schedulers have designs that should enable them to support divergecast patterns, even though they have only been evaluated for convergecast traffic.

The traffic profiles, i.e., the intensity of traffic generated at each node, are heterogeneously distributed in 60 of the surveyed schedulers, as shown in Figure 9b. This matches event-based applications where, e.g., alarms trigger sudden transmissions, or process monitoring where traffic changes according to conditions in the process such as temperatures, pressure, etc. A heterogeneous traffic profile is typically challenging for autonomous and centralized schedulers where the schedule may need to be continuously adjusted to accommodate the changing traffic and stay energy efficient. Homogeneous profiles, where all nodes transmit with the same intensity, are usually simpler to meet. However, this caters to a narrower range of applications, typically limited to monitoring.

### 5.4. Evaluation

In the following sections we go through each category successively, starting with collaborative, autonomous, centralized, static, and lastly hybrid schedulers. For each category we describe common properties, trends, and notable findings for the category as a whole. The schedulers are presented chronologically within each category, except for schedulers that are related, e.g., one being an extension or depends on another; these are addressed after each other chronologically. We apply a bottom-up approach where the surveyed schedulers are analyzed by splitting them into *components*, i.e., the two main decisions:

1. *Cell amount*: Number of cells to be scheduled
2. *Cell selection*: Which cells to select in the slotframe

Concerning collaborative schedulers, one or both of these tasks are solved in a collaborative fashion, while an autonomous scheduler always solves both autonomously. With centralized schedulers, the tasks are typically handled simultaneously: A scheduling algorithm is executed, and the output is a schedule with an appropriate placed cells. When analyzing in this fashion, one will also notice that some proposals only address one of the tasks, typically the cell selection, as in, e.g., DeBraS [71].

Further we evaluate each scheduler to identify design objectives according to the key requirements identified in Section 2: Latency, reliability, fault tolerance, scalability, heterogeneous traffic, throughput, energy, and overhead. We employ a qualitative assessment based on the claimed objectives, the evaluations conducted, and most importantly, we investigate the mechanisms behind the proposed scheduler and to which objective they contribute. These evaluations provide an overview of the research attention given toward each requirement. The rationale behind our assessment is found in the description of each scheduler. These descriptions are omitted here for brevity, yet the interested reader may find them as supplementary material, see end section.

## 6. Collaborative Scheduling

A total of 35 collaborative schedulers were surveyed, making it the most popular approach. Table 3 overviews all these broken down into their components. A significant trend is the incremental improvement of existing work. One prominent example of this is the On-the-Fly Bandwidth Reservation (OTF) [16]: It is an early TSCH scheduler, received significant attention, and was selected by 6TiSCH as the foundation for its Scheduling Function Zero (SF0) (which later became the Minimal Scheduling Function (MSF)). With OTF being a fairly simple scheduler, there was room for different optimizations. This can be seen in Table 3 by looking at the number of schedulers which employ OTF as a component—expanding or enhancing its functionality. Not surprisingly, an overall trend is observed where complexity increases as development progress.

Local collaborative scheduling is the most common with 23 out of the 35 surveyed, as seen in the top section of Table 3. They are typically less complex than recursive and end-to-end schedulers since traffic requirements are not transmitted but inferred from local information such as queue size. Local collaboration may be advantageous in volatile networks with shifting topology and traffic, and its simplicity may also be essential when nodes have limited memory and computation capabilities. However, the convergence suffer in some scenarios since cell allocations are updated re-actively as, e.g., queues are filled. This as opposed to the proactive approach based on updated requirements, e.g., new nodes added, or change in application requirements, which is the case with recursive and end-to-end schedulers. Local collaborative schedulers may therefore be more suitable for applications with less stringent requirements.

With recursive schedulers, the added signaling allows for a more accurate understanding of traffic requirements than a local strategy. Especially in convergecast applications, both local and recursive schedulers may yield similar schedules since local information such as the queue at a node close to the sink also grows recursively. Yet the signaling of actual requirements provides a more precise picture of needs and allows for proactively adjusting allocations as requirements change. This does however come at the cost of additional overhead and increased complexity. Recursive schedulers are thus typically more complex than local schedulers, which may explain why most recursive proposals are more recent and less numerous—11 out of the 35 surveyed.

End-to-end schedulers are the most complex and introduce the most overhead among collaborative schedulers. This must be taken into account if the topology or traffic is volatile, with frequent schedule changes. They do however allow for the most accurate allocations, making it suitable for scenarios with stringent requirements. The only found scheduler is the Completely Fair Distributed Scheduler (CFDS) [72]—leaving much room for research within this category.

When deciding *which cells* to allocate, a substantial portion of collaborative schedulers randomly selects cells in the slotframe. This is especially true for earlier proposals such as OTF. This simple strategy may yield sub-optimal performance for several metrics: For example, LLSF [73] optimizes OTF for shorter latency by selecting cells sequentially towards the sink. A random selection may yield collisions where the same cell is scheduled by two nodes within interference-range. Such collisions are challenging if deterministic performance is required, therefore housekeeping functions have been suggested. These typically monitors each cell performance, and re-negotiate those performing poorly, see, e.g., Muraoka et al. [74]. Later proposals employ more sophisticated collision avoidance algorithms where nodes proactively avoid collisions. This is thus more common with recursive schedulers, and it typically requires nodes to acquire additional information about their neighborhood to avoid contended cells. One local example is DeBraS, where nodes broadcast their schedules, while the recursive scheduler LOST [54] depend on nodes overhearing negotiations between other nodes. Knowledge of the neighborhood schedule may also be exploited to sequentially place cells towards the sink for reduced latency, as seen in recursive schedulers Wave and Kim et al. [75]. However, these improvements typically come at the cost of overhead and increased complexity compared to local schedulers.

Several proposals also focus on performance improvements, and target only one aspect of scheduling, predominantly the selection of cells. As earlier mentioned, extensions to OTF are a classic example of this, but we also find, e.g., SFSB [76] and P-SBC [77] which both do not treat how many cells to schedule.

Deciding the *number of cells* to allocate is typically done in a straightforward fashion, i.e., simply reserving sufficient cells to meet the requirement indicated by various inputs. The overview in Table 3, therefore, does not include a description of the algorithms. The input to local schedulers is only local information such as queue size or cell utilization. With the recursive group, traffic requirements and thus the need for cells are known along a path. For both local and recursive schedulers, later proposals often include ETX when deciding the number of cells to accommodate lossy links. This can be seen in, e.g., ReSF [78] and DeAMON [55]. Some schedulers overprovision the number of cells to accommodate varying traffic and link qualities. This trades reliability with latency and energy as evaluated and addressed in [79,80]. Similar approaches are seen in, e.g., OTF which adds new cells proactively *before* bandwidth estimations require it. LDSF [81] accommodates the worst-case and allocates cells to all possible retransmissions along a path. Lastly, a few schedulers include more sophisticated algorithms, such as EMSF [82] which predicts needs based on Poisson distributed traffic, Local Voting [83] aims for fairness in the neighborhood, and the PID-based (Proportional, Integral, and Derivative) proposal by Doming-Prieto et al. [84] rooted in control theory.

A novel approach is found in SSAP [85] where each node receives exactly one cell. However, this cell is only activated at a slotframe-interval which matches the necessary throughput and latency required by the application. This requires a slotframe length significantly shorter than the expected traffic interval. A similar approach is found in ReSF which allows nodes to specify how often the reserved cells need to be activated to reduce idle listening.

Notably, 20 of the 35 surveyed collaborative schedulers employ a proprietary protocol to disperse necessary information or implement its schedule. This trend might change going forward as the 6P protocol matures. The *Collaboration protocol* column in Table 3 shows which protocol is utilized to build the schedule or exchange information between collaborating nodes. This highlights those proposals using standardized solutions (typically the 6P protocol), and those requiring new protocols or extensions. All proposals utilize a protocol to implement the schedule, e.g., through negotiation as with 6P. The *Information* sub-column indicates if the protocol is also used to disperse information, such as traffic requirements or schedule density, as in DeTAS [52] and E-SF0, respectively. This may introduce a prolonged convergence period for these schedulers as new information must be dispersed before the schedule is accurately updated.

As collaborative approaches rely on information exchanged between nodes, it is worth noting that few schedulers consider the performance of the utilized protocol. Similar to regular traffic, these exchanges are also prone to delays and failures which might impact a scheduler’s performance [86]. Furthermore, the used protocol dictates the amount of overhead introduced with every negotiation. An evaluation of the 6TiSCH 6P protocol can be found in [87], where they identify 6P parameters such as timeout limits and retransmission- and transmission opportunities for optimal performance in their grid scenarios.

In most schedulers, all nodes have the same role with regard to scheduling. The *Non-uniform* column in Table 3 shows the 11 schedulers which require some nodes to take on special roles, of which most are recursive. Such special roles are typically assigned to the sink node, which becomes responsible of, e.g., initiate the scheduling, or collect and disperse traffic requirements. In, e.g., Wave, scheduling is started at a leaf node to ensure a daisy-chaining of cells towards the sink node to shorten latency. This allows the collaborative scheduler to get a sense of global coordination typically reserved for centralized schedulers. One might argue this makes these schedulers hybrid, yet since the actual scheduling is done through negotiations between neighboring nodes, we classify them as collaborative. Schedulers with non-uniform roles may also employ phases such as in Stripe [88], where the schedule is built in a separate scheduling period before the network is operational. This may negatively impact heterogeneous traffic support since adapting the schedule requires executing a new scheduling period.

A part of the increasing scheduler complexity is handling the channel hopping list. This is typically motivated by external interference from, e.g., co-located networks, causing a subset of the channels to perform poorly. Three proposals incorporate such mechanisms, as indicated in the *Hop-list* column. Implementations typically involve maintaining blacklists, which identify poor-performing channels to be avoided, see, e.g., LOST and P-SBC.

The objectives of all collaborative schedulers is seen in Table 4. Most proposals target deterministic latency, reliability, energy and heterogeneous traffic. This is expected since the collaborative strategy allows nodes to negotiate for resources dynamically. Thus, when required, nodes typically schedule additional cells to, e.g., accommodate a surge in traffic or reduced link quality, or release cells to conserve energy when possible.

Of the least addressed objectives, we find fault tolerance, which few schedulers address. One example is by Yoo et al. [89] which always schedules resources to multiple alternative parents to improve fault tolerance and introduce load balancing. Another approach is seen in Wave [90] which first evaluates any new links and triggers a re-scheduling only if it causes collisions or if a new parent is selected.

**Table 3:** Components of collaborative TSCH schedulers.

Scheduler	Strategy	Cell Selection		Cell Amount	Collaboration Protocol		Non-Uniform	Hop-List
		Algorithm	Input	Input	Name	Info.		
OTF [16]	Local	Random	Node schedule	Cell utilization	6P			
LLSF [73]	Local	Sequential	Node schedule	OTF	6P			
Muraoka et al. [74]	Local	Random	Node schedule, PDR	OTF	6P			
DeBraS [71]	Local	Collision avoidance	Neighborhood schedule	OTF	6P + own	✓		
E-SF0 [91]	Local	Collision avoidance	Neighbor schedule density	OTF	6P + own	✓		
Fahs et al. [92]	Local	Collision avoidance	Neighborhood schedule	OTF	6P			
E-OTF [93]	Local	Random	Node schedule	OTF, ETX	6P			
ASAP [94]	Local	Random	Node schedule	Fixed	Own			
TREE [95]	Local	Random	Node schedule	Queue, Cell utilization, ETX	6P			
Doming-Prieto et al. [84]	Local	Random	Node schedule	Queue, cell utilization	6P			
Zhang et al. [96]	Local	Random, blacklist	Node schedule, RSSI meas.	Queue, cell utilization	Own			
$SF_{loc}$ [97]	Local	Random or sequential	Node schedule	Queue, ETX	6P			
Hosni et al. [59]	Local	Sequential, random	Hop count	$SF_{loc}$	6P			
P-SBC [77]	Local	Best channel	PDR estimate	N/A	Own	✓		✓
Stripe [88]	Local	Sequential	Node schedule	Child count	Own		✓	
SFSB [76]	Local	Random, blacklist	Node schedule, RSSI meas.	Fixed	6P	✓		✓
Yoo et al. [89]	Local	Random	Node schedule	RX/TX statistics, ETX	6P + own	✓		
Instant [98]	Local	Fixed	Node requests	Neighborhood mobility	Own	✓	✓	
EMSF [82]	Local	Random	Node schedule	Traffic	6P			
SIM [99]	Local	Latin Rectangle	Node schedule	Traffic	6P			
REA-6TiSCH [100]	Local	Opportunistic	Node schedule, traffic type	Packet characteristics	Own	✓		
Local Voting [83]	Local	Random	Node schedule	Neighborhood traffic	6P + own	✓		
OA-TSCH [101]	Local	Collision avoidance	Distributed channel	Queue	Own	✓	✓	
DeTAS [52]	Recursive	Queue min., sequential	Rank, parent	Traffic requirement	Own	✓	✓	
Wave [90]	Recursive	Sequential, coll. avoid.	Neighborhood schedule	Traffic requirement	Own	✓	✓	
DiSCA [102]	Recursive	Sequential, coll. avoid.	Neighborhood schedule	Traffic requirement	Own	✓	✓	
DeAMON [55]	Recursive	Coll. avoid., sequential	Neighborhood schedule	Traffic req., ETX, rank	6P + own	✓	✓	
ReSF [78]	Recursive	Coll. avoid., sequential	Application, node schedule	Traffic req., ETX, queue, path	6P + own	✓		
LOST [54]	Recursive	Collision avoidance	Neighborhood schedule	Traffic req., PER	Own	✓		✓
Kim et al. [75]	Recursive	Sequential	Neighborhood schedule	Traffic	6P		✓	
DIVVY [103]	Recursive	Collection avoidance	Neighborhood schedule	Traffic, cell stats.	Own	✓		
LaDiS [104]	Recursive	Sequential	Neighbor schedule	Traffic requirement	Own	✓	✓	
SSAP [85]	Recursive	Collision avoidance	Parent schedule	Neighborhood sched., path delay	Own	✓	✓	
LDSF [81]	Recursive	Sequential, random	Hop count	Max. retransmissions, hop count	6P			
CFDS [72]	End-to-End	Sequential, Random	Rank, traffic, blacklist	Traffic requirement	RSVP-TE		✓	

Short latency is also often not targeted because it typically requires global coordination to allocate cells in a daisy-chain manner from leaves to sink. One approach to solving this is through non-uniform roles discussed earlier. As pointed out in Table 3, most recursive schedulers have this property, where some nodes are responsible for, e.g., initiating or coordinating the schedule generation., e.g., in DeAMON [55], the sink node transmits a build-command to leaf nodes to initiate scheduling. With the process working upwards from the leaves, it achieves daisy-chaining and thus shorter latency.

**Table 4:** Evaluation of collaborative TSCH schedulers.

Scheduler	Objectives								
	Det. Latency	Short Latency	Reliability	Fault Tolerance	Scalability	Hetero. Traffic	High Throughput	Energy	Overhead min.
OTF [16]	✓		✓			✓		✓	✓
LLSF [73]	✓	✓	✓			✓		✓	✓
Muraoka et al. [74]	✓		✓		✓	✓		✓	✓
DeBraS [71]	✓		✓		✓	✓		✓	✓
E-SF0 [91]	✓		✓		✓	✓		✓	✓
Fahs et al. [92]	✓		✓		✓	✓		✓	✓
E-OTF [93]	✓		✓	✓		✓		✓	✓
ASAP [94]				✓					✓
TREE [95]	✓		✓			✓		✓	
PID-based [84]	✓		✓			✓		✓	✓
Zhang et al. [96]	✓		✓			✓		✓	✓
$SF_{loc}$ [97]	✓	✓	✓			✓		✓	
Hosni et al. [59]	✓	✓	✓			✓		✓	
P-SBC [77]		✓	✓			✓			
Stripe [88]	✓	✓							✓
SFSB [76]			✓						
Yoo et al. [89]				✓				✓	
Instant [98]				✓		✓	✓	✓	✓
EMSF [82]	✓				✓	✓			✓
SIM [99]	✓		✓			✓		✓	
REA-6TiSCH [100]	✓	✓	✓			✓			
Local Voting [83]	✓		✓			✓		✓	
OA-TSCH [101]	✓		✓			✓			
DeTAS [52]	✓	✓	✓		✓		✓		✓
Wave [90]	✓	✓	✓	✓		✓	✓		
DiSCA [102]	✓	✓	✓			✓	✓		
DeAMON [55]	✓	✓	✓	✓	✓	✓	✓	✓	✓
ReSF [78]	✓	✓	✓		✓	✓		✓	✓
LOST [54]	✓		✓		✓	✓		✓	✓
Kim et al. [75]	✓	✓	✓			✓			
DIVVY [103]	✓	✓	✓			✓		✓	
LaDiS [104]	✓	✓			✓	✓	✓	✓	
SSAP [85]			✓	✓		✓		✓	
LDSF [81]	✓	✓	✓					✓	✓
CFDS [72]	✓	✓	✓			✓		✓	

Similarly, schedulers seldom optimize for high throughput, and this typically requires short slotframes such that cells repeat often. However, most collaborative approaches avoid this because smaller slotframes increase the chance of scheduling collisions since there is no global coordination. The few schedulers who address this utilize non-uniform roles to achieve this. The common approach, e.g., in DeTAS [52], is for the sink node to learn traffic requirements, calculate an optimal slotframe length, and lastly signal this to all other nodes when initiating the scheduling process.

Lastly, scalability is rarely addressed directly. It is however crucial in collaborative scheduling since nodes do not have a network-wide view, and interfering nodes may schedule the same cells. This problem is exaggerated as the network scales and becomes denser. Mechanisms to mitigate this typically involve nodes learning more about their neighborhood and using this to implement more intelligent cell selection. Examples employing this include the previously discussed DeBRAS and LOST, and E-SF0 [91] where neighbors share the density of their schedule. A reactive approach is suggested in Muraoka et al. [74], where a housekeeping mechanism aims to identify and re-schedule any colliding cells.

Scalability also ties in with overhead minimization since the number of negotiations needed may increase non-linearly as the network grows. This is especially true for topologies with mobile nodes or varying traffic patterns, where a change may trigger re-negotiations across the network. Most schedulers address this with mechanisms such as overprovisioning and hysteresis, as in OTF, or the more sophisticated PID-based algorithm mentioned earlier. The aim is to set the number of cells so that varying requirements do not trigger re-scheduling, and not waste energy on idle listening.

## 7. Autonomous Scheduling

With autonomous scheduling, each node independently generates a schedule without dedicated communication between neighbors or a central entity. It typically exploits other existing sources of information to deduce which cells to utilize. These sources may include node identifiers or addresses, cross-layer routing information such as depth in RPL tree, or time. This eliminates overhead from the scheduling process as the information utilized is already maintained by other network functions. Thus, no additional energy or bandwidth is needed to build the schedule. In addition, autonomous setups should allow for faster convergence in cases of node joining, faults, and similar—especially compared to centralized approaches where a Path Computation Entity (PCE) must be involved. Its simplicity also allows operators improved understanding of the network a priori, and easier monitoring and debugging at run-time. Lastly, with little or no need for configuration, an autonomous scheduling deployment may be more straightforward and require minimal knowledge. This could open up the usage to a broader audience, where it was previously considered too complex.

The major drawback of the current autonomous proposals is their inability to adapt to changing conditions such as network size or heterogeneous traffic while fulfilling industrial requirements for latency and reliability. Similarly, it is challenging to employ techniques such as spatial reuse of cells or optimize for minimal slotframe size since this typically requires collaboration.

Based on these properties, autonomous schedulers are typically favored in use-cases without strict deterministic requirements. These include generic IoT, as seen with Orchestra [53], or for bootstrapping or fallback from more optimized schedules, as seen in 6TiSCH Minimal Scheduling Function (MSF) [48].

Autonomous scheduling is a fairly recent topic with TSCH. However, similar techniques have been utilized in earlier MAC proposals in, e.g., the Time Division Hashing (TDH) (2005) [105] scheduling scheme and the Crankshaft MAC protocol (2007) [106]. As such, only seven TSCH autonomous schedulers are found. Orchestra [53] being the first, it has garnered significant attention as was seen in the timeline in Figure 6. ALICE [56] expands on Orchestra, improving especially its scalability.

Escalator [107] is a significant development that addressed some of the limitations of Orchestra by including rank into the algorithm input. This allowed an autonomous scheduler targeting deterministic and short latency by utilizing rank and source ID to allocate dedicated cells sequentially at every hop towards the sink. Layered [108] continued in the same fashion but introduced autonomous spatial reuse to reduce the channels occupied by the scheduler—an objective seldom addressed. More recent approaches such as BOOST [109] and Phung et al. [110] are opportunistic, relying on reducing contention instead of elimination. Note Phung et al., which uses a novel trial-and-error scheme where all nodes randomly try to transmit and receive packets during a learning phase to identify appropriate cells to insert into the schedule. This might be viewed as a collaborative approach, with the communication happening implicitly through successful and erroneous transmission attempts.

Autonomous schedulers are inherently much simpler compared to the collaborative strategy. This is reflected in Table 5, which overviews the components. Most schedulers use a simple hash algorithm to decide which cells to select. The hash may yield a collision-free schedule, however this typically requires the number of nodes to be less than the slotframe length. ALICE mitigates this problem by ensuring any collisions are not persistent: It adds time as an input, represented by Absolute SlotFrame Number (ASFN) (A global counter indicating the current slotframe since network deployment, defined as ASN (see Section 3) divided by slotframe length), such that the hash-output and thus schedule is different for every slotframe.

Three out of the seven schedulers utilize a single node id when selecting cells. These may be categorized as node-based schedulers, i.e., they assign cells to particular nodes. On the contrary, ALICE is link-based since it employs node ids from both sides of a link in addition to the traffic direction and thus assigns cells to particular links. They argue this better matches the characteristics and needs of the network since, e.g., downward and upward traffic receives separate resources. Lastly, Escalator and Layered may be denoted as flow-based schedulers as they assign cells to particular flows. This opens unexplored possibilities such as sharing flows between multiple nodes or differentiated scheduling by, e.g., adding overprovisioning only to specific flows.

Most autonomous schedulers allocate a fixed amount of cells to each node or link. They rely on overprovisioning to accommodate for heterogeneous traffic intensity and tackle retransmissions. Elst. et al. [111] adds shared cells that are utilized opportunistically based on queue size for this purpose. Similarly, Phung et al. propose nodes to employ trial-and-error allocations for the number of required cells.

One of the key traits of autonomous schedulers is their lack of dedicated communication to build the schedule; hence it does not introduce any overhead. This is reflected when evaluating the objectives in Table 6. One exception is Phung et al. which inevitably introduce overhead during its learning phase. The lack of dedicated communication also yields an inherent tolerance to faults, as there are no negotiations, recovery mechanisms, or similar to be executed when the topology changes.

Several objectives are however still largely untreated in the autonomous domain. These include the ability to uphold requirements as the network scales, as discussed in Section 2. Although autonomous schedulers do not introduce overhead, all current proposals either assume a fixed maximum number of nodes, or have performance issues as the amount of nodes grows. Another crucial problem is the ability to handle heterogeneous and changing traffic demands. This also makes the common convergecast scenario challenging for autonomous schedulers since it creates a funneling effect where additional cells are needed close to the sink. As discussed earlier, most proposals utilize a fixed amount of cells, relying on overprovisioning or contended cells. Contention rules out the possibility of achieving deterministic latency since the varying throughput make a pratical bound on the latency unattainable. Escalator and Layered are the only schedulers that tackle this in a deterministic fashion by allocating cells sequentially at each rank for every node—thus also aiming at deterministic latency. Elst et al. and Phung et al. address this in opportunistically by employing shared and contended cells. The remaining proposals use probabilistic overprovisioning, as discussed earlier. The rigidity of autonomous schedules also impedes energy efficiency since schedulers typically allocate a fixed number of cells regardless of actually offered traffic. BOOST tries to overcome this by monitoring cell utilization and periodically ignoring cells that have not seen traffic for a time. Lastly, Elst et al. aims to improve throughput by allowing nodes to reuse cells that are known to be available in their sub-tree. This technique relies on nodes learning about their tree through RPL.

Common solutions to the open challenges typically involve exchanging information between nodes, which is not an option in an autonomous approach. Thus, if novel solutions are not put forward, combinations with other strategies into hybrid schedulers might be necessary. However, both algorithms and inputs were limited in variety, as highlighted in Table 5. Indeed, the entire set of input includes only node id, source id, rank, time, direction and queue size—leaving many facets unexplored. As a final point, it is worth noting that most autonomous schedulers have been evaluated in a testbed or actual deployment. This is contrary to other scheduling categories and might be explained by the proposals being of newer date, with testbeds and equipment continuously maturing and becoming more available. A survey on autonomous scheduling may be found in [49] (This includes “non-pure” autonomous schedulers where information is exchanged in order to build the schedule—categorized as collaborative or hybrid schedulers in our survey).

## 8. Centralized and Static Scheduling

In the centralized approach, a single Path Computation Element (PCE) running a scheduling algorithm is typically employed to generate and distribute the schedule. Usually, the algorithms require detailed information from the network, including, e.g., node capabilities, wireless link properties, routing- and physical topologies, etc. Using the collected information, it is possible to create highly optimized schedules. However, frequent changes in network properties, e.g., mobile nodes, changing radio environments or altered application requirements, may be challenging to accommodate in a timely manner without significant overhead [112,113]. This is especially true as the network scales and the number of nodes to collect from and distribute information to increases. Thus, centralized schedulers may be preferred when traffic, topology, and environment are less volatile such that re-scheduling is rarely needed. In total, we surveyed 22 centralized schedulers, in addition to two static schedulers, which will be treated at the end of this section.

The centralized strategy is utilized by existing industrial solutions such as, e.g., WirelessHART [3], implemented by its *Network Manager*. It also fits well into the Deterministic Networking (DetNet) architecture [114]: Achieving the deterministic traffic flows envisioned by DetNet requires a capability to reserve bandwidth, e.g., schedule cells, throughout the network. This may be realized by a centralized TSCH scheduling approach, as discussed in [115].

The common approach for centralized schedulers is to formalize the scheduling problem, identify an optimal solution, and lastly apply this solution into a scheduler. With the problem typically being NP-hard, implementations are usually approximations, as seen with ADP [116]. The solutions often involve an allocation algorithm rooted in graph theory such as with TASA [51] and PRCOS [117], yet may be simpler heuristic algorithms such as found in EES [118]. Of the 22 surveyed schedulers, TASA is especially notable since it has had a significant influence on other centralized proposals, similar to the role of Orchestra for autonomous and OTF for collaborative. Many succeeding schedulers employ TASAs models or expand the scheduler itself, such as TASA-RTX [119] which extends TASA to improve the handling of lossy links.

As mentioned, several algorithms stem from graph theory. See, e.g., *coloring* by TASA, PRCOS and SPRF in Table 7 which shows all algorithms and their input. A novel approach can be found in CONCISE [120] where multiple functions such as routing, in-network processing, aggregation, etc., is treated alongside the schedule in a cross-layer approach. With centralized scheduling being a fairly mature category, a range of eight different algorithm inputs have already been proposed. Almost all schedulers require topology knowledge, and 12 out of the 22 surveyed schedulers require knowledge of the physical topology, i.e., all neighbors known to a node. This is typically utilized to avoid interference and allow for *spatial reuse* where multiple nodes use the same cell. A novel approach is proposed by Ojo et al. in [118] and EES, which combine knowledge of physical distance with an interference model to deduce collision-domains.

Most schedulers limit their input to topology information and offered traffic. This may be impeding in real deployments since several other factors may impact the network operation. Examples include link quality, node energy, queue size, and link utilization, which may change during network operating. Only nine schedulers augment their algorithm with such inputs.

This is contrary to the trend found in collaborative schedulers which increasingly utilized, e.g., estimated transmissions attempts (ETX) to improve reliability. The effect of this is seen in Table 8: Whereas centralized schedulers typically address latency, few proposals focus on the key industrial requirement of reliability by taking, e.g., link quality and queue sizes into account. Lacking knowledge of such properties may be an issue when schedulers are utilized in real-world scenarios. An illustrative example is found in EES, which optimizes the schedule for energy efficiency and network lifetime, yet does not consider the energy available at each node.

Note however that such considerations may be difficult to realize in a centralized scheduler, as it typically would require frequent exchanges of, e.g., link statistics and schedule updates between nodes and a central entity. This could lead to an unacceptable overhead, as well as it would be challenging to ensure the schedule adapts quickly enough to maintain the required latency and reliability. Examples of approaches in this direction include TASA-RTX which employs link quality to support retransmission capabilities, and PRCOS which utilize node energy when optimizing the schedule for network lifetime.

The issues of scalability and overhead, especially in dynamic scenarios, are typically not addressed in centralized scheduling, as seen in Table 8. One recent proposal has focused on this: By adding shared overprovisioned cells assigned to particular flows, MASTER [121] allows the schedule to adapt to fluctuations in ETX across multiple links without re-scheduling. Furthermore, as discussed in the next section, hybrid schedulers such as CLS [122] and QSS [123] show how a combination of strategies may be employed to mitigate this issue. Similarly, fault tolerance is typically not addressed or mentioned in proposals. It is thus clear that most centralized schedulers require a costly re-scheduling process in cases of faults. Notable exceptions include Wu et al. in [124] which proactively schedule alternate links. Centralized schedulers may therefore be more suitable when topology and links are stable and less numerous.

**Table 7:** Components of centralized TSCH schedulers.

Scheduler	Algorithm	Input							
		Physical Topol.	Routing Topol.	Geographic Topol.	Offered Traffic	Link Quality	Node Energy	Queue Size	Link Utilization
TASA [51]	Coloring & matching	✓	✓		✓				
TASA-RTX [119]	Coloring & matching, Inverse Greedy	✓	✓		✓	✓			
Farias et al. [125]	Queue-based		✓		✓				
MODESA [126]	Greedy MODESA	✓	✓		✓				
Wu et al. [124]	Margin slots	✓	✓		✓				
Yang et al. [127]	SSA, FSC, free node		✓						
Dawn [128]	Not specified				✓				
Chen et al. [129]	LSS & LPS	✓	✓						
Ojo et al. [130]	Hungarian			✓				✓	✓
EES & V-H. [118]	Greedy, VAM			✓				✓	✓
ADP [116]	Approximate Dynamic Programming	✓				✓		✓	
Khoufi et al. [131]	Debt-based		✓		✓				
PRCOS [117]	Coloring & pruning, Cross-layer	✓					✓		
MILS [132]	Constrained Satisfaction Problem	✓							
Minet et al. [133]	Debt-based		✓		✓				
CONCISE [120]	Cross-layer	✓			✓				
Devaja et al. [134]	Message-passing max-product belief prop.	✓	✓					✓	
SPRF [135]	Coloring & matching, blossom & heuristic	✓	✓		✓				
Khorov et al. [136]	Retry & shared cell optimization				✓	✓			
Brun-Laguna et al. [137]	Load-based		✓		✓	✓			
MASTER [121]	Flow-based TX & Reverse Longest Path First	✓			✓	✓			
Portaluri et al. [138]	Shell-game-based				✓				

Heterogeneous traffic is typically addressed since most centralized schedulers take the offered traffic or similar information from each individual node as input when building the schedule. However, similar to when faults occur, any changes while the network operates may require significant overhead to accommodate. A few schedulers such as Chen et al. [129] and PRCOS [117] strictly assume homogeneous traffic intensity and does not utilize information on offered traffic, queue size, or similar. Thus, support for heterogeneous traffic is not aimed for, as indicated in Table 8. SPRF [135] is the only centralized scheduler that directly addresses a divergecast traffic scenario. This might be surprising but follows the trends seen in the other surveyed categories where convergecast dominates. However, based on the algorithms utilized, most proposals should also be able to accommodate divergecast traffic.

**Table 8:** Evaluation of centralized and static (bottom) schedulers.

Scheduler	Objectives								
	Det. Latency	Short Latency	Reliability	Fault Tolerance	Scalability	Hetero. Traffic	High Throughput	Energy	Overhead min.
TASA [51]	✓	✓				✓	✓		
TASA-RTX [119]	✓	✓	✓			✓	✓		
Farias et al. [125]	✓					✓			
MODESA [126]	✓	✓				✓	✓		
Wu et al. [124]				✓		✓			
Yang et al. [127]	✓	✓	✓	✓					
Dawn [128]	✓					✓			
Chen et al. [129]	✓	✓					✓		
Ojo et al. [130]	✓					✓	✓		
EES & VAM-HSA [118]	✓					✓		✓	
ADP [116]	✓	✓	✓			✓		✓	
Khoufi et al. [131]	✓	✓				✓	✓		
PRCOS [117]	✓	✓						✓	
MILS [132]		✓						✓	
Minet et al. [133]	✓	✓		✓		✓	✓		
CONCISE [120]	✓	✓				✓		✓	
Devaja et al. [134]	✓		✓			✓	✓		
SPRF [135]	✓		✓			✓	✓		
Khorov et al. [136]	✓	✓	✓			✓			
Brun-Laguna et al. [137]	✓		✓			✓		✓	
MASTER [121]	✓		✓		✓	✓			✓
Portaluri et al. [138]	✓					✓			
Park et al. [139]			✓	✓	✓	✓			✓
Khorov et al. [136]			✓						

With regards to throughput, we saw in Table 7 how most centralized schedulers are aware of the physical topology in the network. This may be used to incorporate spatial reuse in the slotframes, thus reducing the slotframe length and improving throughput. However, experimental evaluation is needed to ensure models and assumptions for the interference range hold in real-world scenarios.

Lastly, it should be noted that several centralized proposals make assumptions such as, e.g., perfect links in MODESA and data aggregation in [129] by Chen et al. Such assumptions are unrealistic for actual deployments. Corroborating this, only three of the surveyed schedulers were evaluated in a testbed or actual deployment. This is somewhat surprising, given the utilization of centralized schedulers in tightly related technologies such as WirelessHART.

Only two static schedulers were identified, which may not be surprising given the strategy’s simplicity and limitations. With static scheduling, a fixed schedule is shared among all nodes either before deployment or learned at the first association. It is typically envisioned for bootstrapping the network, association of new nodes, or as a fallback in case of failures. For example, a static schedule is specified by IETF in the “Minimal 6TiSCH configuration” [47], consisting of a slotframe with one shared broadcast cell. Park et al. [139] propose a static scheduler for a smart metering application where a large number of devices periodically report data. The suggested static schedule is similar to a slotted ALOHA approach where reliability and latency objectives are met through significant overprovisioning, which is traded for increased energy consumption. Khorov et al. [136] focus on how to a priori identify an optimal amount of shared cells in a slotframe—similar to work done by Elst et al. [111] in an autonomous setting.

## 9. Hybrid Scheduling

Hybrid scheduling combines multiple strategies, trying to leverage the benefits of each approach while mitigating the drawbacks. Most hybrid proposals take existing schemes and address deficiencies by employing a second strategy of scheduling. Several examples include the autonomous scheduler Orchestra, which is expanded with several new mechanisms producing multiple hybrid schedulers. For proposals based upon a centralized component, most opt to take a minor part of the scheduling task and solve collaboratively to improve some properties. The ten surveyed hybrid schedulers can be seen in Table 9, which shows the strategies employed to address each component. As indicated in the table, the schedulers can be divided into two groups according to the strategies they expand upon.

First there are schedulers who in varying degrees expand on a static or autonomous foundation by adding a collaborative mechanism. This is typically to alleviate some of the inherent drawbacks of a particular scheduler or strategy. Examples include PAAS [140] and e-TSCH-Orch [141], both targeting the lack of support for heterogeneous traffic in autonomous scheduling. They address this by expanding Orchestra with collaborative mechanisms which allow nodes to exchange scheduling adjustments as traffic intensity changes. This will however negate some of the benefits of autonomous scheduling such as minimal overhead and fault tolerance. Fafoutis et al. [142] is the only hybrid scheduler that includes a static strategy. They aim to improve the energy-efficiency in an overprovisioned static schedule by having nodes exchange information on how many of the overprovisioned cells they intend to use.

Table 10 shows the components of the first group of schedulers. The table follows the format of the collaborative schedulers (Table 3) as it provides the most valuable insight. Except for PAAS, the collaborative contribution is limited to the number of cells to be utilized., e.g., with e-TSCH-Orch, nodes transmit the additional cells needed, based on current queue size, at the end of each packet. PAAS adjusts the level of contention among child nodes by schedule instructions added to RPL packets.

Note that none of the schedulers utilize the standard 6P protocol to schedule cells. This may be explained by the limited functionality required: Typically, nodes only exchange a single number such as the cells required, as by Fafoutis et al. and e-TSCH-Orch. The inclusion of a protocol such as 6P might therefore introduce unnecessary overhead that could indicate the need for a more lightweight protocol when simplistic collaboration is required. It may also be noted that all schedulers employ local collaborative mechanisms (as opposed to recursive or end-to-end), i.e., they employ only local information such as queue sizes or traffic load instead of exchanging requirements. More complex collaboration may be an area for future research.

The second group consists of schedulers who expand on centralized schemes, again to improve some inherent disadvantages such as overhead and scalability. In all surveyed schedulers, this involves adding a collaborative mechanism, as illustrated in Table 9. Further details can be seen in Table 11 which shows the components of each scheduler. The collaborative expansions are typically limited, as indicated in the final column. MABO-TSCH [57] adds a collaborative mechanism to modify the channel hop-list for improved reliability, while the remaining functionality is handled in a centralized fashion (it should be noted that this is one of the few schedulers which optimize the hop-list, as discussed in Section 6) AMUS [143] adds the exchange of a simple end-of-queue signal so that remaining cells in the current slotframe will not incur idle listening. CLS [122] and QSS [123] allows deallocations to be conducted in a collaborative fashion for reduced signaling. Lastly, Kaaragac et al. [113] employ a different approach, by envisioning critical traffic to be centrally scheduled, while other traffic classes are handled collaboratively via OTF [16]. It may be argued this is not a hybrid scheduler but rather two schedulers operating simultaneously. Such approaches may however be an interesting area for future research, and are discussed later. Furthermore, almost all hybrid proposals focus on cell *deallocation* in a decentralized fashion. This leaves an open area of handling *allocation* through decentralized means.

**Table 9:** Overview of components in hybrid TSCH schedulers.

Scheduler		Cell Selection	Cell Amount	Hop-List
Fafoutis et al. [142]		Static	Collaborative	
PAAS [140]		Collaborative	Autonomous	
e-TSCH-Orch [141]		Autonomous	Collaborative	
TESLA [144]		Autonomous	Collaborative	
OST [145]		Autonomous	Autonomous & Collaborative	
MABO-TSCH [57]		Centralized	Centralized	Collaborative
AMUS [143]		Centralized	Centralized & Collaborative	
Karaagac et al. [113]		Centralized & Collaborative	Centralized & Collaborative	
CLS [122]		Centralized	Centralized & Collaborative	
QSS [123]		Centralized	Centralized & Collaborative	

**Table 10:** Components of decentralized hybrid TSCH schedulers.

Scheduler	Cell Selection			Cell Amount
	Algorithm	Input		Input
Fafoutis et al.	Static	N/A		Queue, cell utilization
PAAS	Collision optimization	Neighborhood		Fixed (varying contention)
e-TSCH-Orch	Autonomous	Node ID		Queue
TESLA	Autonomous	Node ID		Traffic
OST	Autonomous	Node ID		Traffic

**Table 11:** Components of centralized-based hybrid TSCH schedulers.

Scheduler	Algorithm	Input								Decentralized Part
		Physical Topol.	Routing Topol.	Geographic Topol.	Offered Traffic	Link Quality	Node Energy	Queue Size	Link Utilization	
MABO-TSCH	Greedy degree-ordering	✓								Hop-list
AMUS	Scheduling Sequence Matrix	✓	✓		✓					Temporary dealloc.
Karaagac et al.	Not specified	✓	✓		✓					Application depend.
CLS	Greedy CLS		✓							Deallocation
QSS	Greedy CLS, QSS optimization		✓							Deallocation

As mentioned, the motivation behind hybrid schedulers is often to address a limitation or drawback of existing schedulers or strategies. This is true for, e.g., PAAS, e-TSCH-Orch and TESLA which inherit the objectives of Orchestra, yet enhance it by adding support for heterogeneous traffic. Table 12 shows the objectives targeted by all the hybrid schedulers. The trade-off is typically a minor increase in overhead and complexity. Similarly, Fafoutis et al. add the same feature to a static scheduler. In the second group of centralized-based schedulers, drawbacks of the strategy are typically addressed. AMUS addresses the possible energy inefficiency of centralized schedules which may be sub-optimal in, e.g., periods with less traffic than expected. Similarly, the collaborative cell deallocation in CLS and QSS is intended to mitigate the overhead issue in centralized scheduling by not requiring a new schedule to be calculated and distributed when cells are no longer needed.

With only ten hybrid proposals identified, there are still many unexplored facets. In addition to those mentioned above, the combination of autonomous and centralized scheduling is still untreated. Similarly, more extensive inclusions of other strategies in centralized schedulers may be explored. Further perspectives on hybrid scheduling may be found in Karaagac et al. [113] which discuss several hybrid strategies not implemented by schedulers in our survey. These include having different traffic classes be scheduled by different means: Critical traffic could follow an optimized centrally generated schedule, while regular traffic follows a decentralized schedule. Similarly, one could differentiate between roles in a topology: Backbone nodes could follow a more rigid centralized schedule, and leaf nodes a dynamic decentralized one. A similar concept is discussed in [115], where a central scheduler provides an overall global perspective and a distributed part handle local real-time variations.

## 10. Future Research Areas and Current Challenges

Through our work with the survey, and by comparing the requirements described in Section 2 with our findings in the previous sections we have identified the following challenges and future research areas.

From Table 6 and Table 12 we see that the autonomous and hybrid areas have seen few published works, leaving many facets unexplored: Autonomous approaches have appealing properties for industrial setting in terms of fault tolerance and zero overhead. However, they have unresolved challenges, e.g., for heterogeneous applications where the traffic is dynamic, or accommodating retransmissions. This also impacts energy efficiency, as the same number of cells is typically allocated regardless of traffic requirement. Flow-based autonomous scheduling contains some uncharted options as exemplified in Section 7 which may be interesting, e.g., in networks with many nodes and few flows, or in combination with flow-concepts from DetNet.

The benefits of hybrid solutions, as well as different combinations of centralized and collaborative schedulers, were surveyed in [113]—with promising results. Yet other unexplored combinations might also prove fruitful, e.g., employing autonomous approaches or partly dividing scheduling mechanisms—as discussed in Section 9. A final related point: The decentralized strategy of end-to-end scheduling, where a reservation is handled end-to-end, has received limited attention, yet seems to have promising properties when determinism is required. The only found contribution is CFDS, which utilizes the existing RSVP protocol—leaving room for further research into end-to-end scheduling and its reservation protocols. This may be particularly relevant when considering the DetNet concepts regarding *flows*.

Few collaborative and centralized schedulers address the issue of node or link faults, as was seen in Table 4 and Table 8. Such faults may lead to violations of requirements such as reliability or latency if not handled properly. Autonomous scheduling is an interesting field in this regard, as it has inherent handling of faults by not requiring any dedicated communication to create a new link. As mentioned above, a hybrid approach containing an autonomous part may be interesting.

Surprisingly, most centralized schedulers assume perfect links, they do not consider retransmissions, or they fail to offer any mechanism adapting to changing link characteristics. Such assumptions are typically not realistic: As discussed in Section 3, the industrial wireless environment is prone to varying channel quality, and mechanisms to assure reliability and other requirements during fading events seem to be necessary. For collaborative schedulers, this is often tackled by, e.g., taking link ETX into account, applying overprovisioning, utilizing housekeeping mechanisms to avoid poor performing cells, etc. Yet results show reliability may still suffer due to, e.g., scheduling collisions, especially when density increases—as illustrated in DeBraS.

Scalability has been given limited attention in publications, yet it is a crucial requirement for the Industrial Internet of Things. Especially centralized schedulers may suffer significant overhead in large networks and when frequent re-scheduling is needed. Only MASTER was found to address this directly by overprovisioning shared cells. Interesting contributions regarding scalability were found in hybrid solutions, detailed in Table 7, where centralized schedulers are supplemented with decentralized features: Both CSS and QSS reduce the necessary signaling by employing a decentralized deallocation mechanism. An investigation into the distribution of centrally calculated schedules in 6TiSCH has been conducted in [146]. It suggests several changes to the standard protocols which may significantly increase the efficiency of transmitting schedules from a PCE to nodes—indicating more research is needed on the topic. Similarly for collaborative schedulers, the overhead associated with the collaborative protocol itself is often not considered.

The 6P protocol is enjoying wide popularity, as highlighted in Table 3, however, experimental evaluations have shown transaction failures may be significant and should be taken into account when designing schedulers [86]. Further investigations into the properties of 6P may thus be prudent. Lastly, it is not uncommon for schedulers to distribute information by piggy-backing on RPL-, 802.15.4-, or application-packets. An increased understanding is needed of these different options, and if a dedicated protocol could be beneficial.

Few proposals directly address the issue of band occupancy, i.e., channels potentially utilized. Although optimizing for minimal overhead is common, the number of channels employed is often overlooked, especially in decentralized solutions. By leaving channels unused, it eases interoperability with co-existing deployments and allows for improved reliability through blacklisting techniques, e.g., with the Escalator autonomous scheduler, all 16 channels in the 802.15.4 2.4 GHz band are required in its full configuration—an issue addressed by the Layered scheduler. Similarly, all the surveyed schedulers assume fixed size timeslots in one band, typically 2.4 GHz. Recent work has challenged this assumption by exploring multi-PHY approaches [147,148] and heterogeneous timeslot duration [149].

The majority of work on TSCH is focused on convergecast, i.e., many devices delivering data to one sink. Clearly, this is the dominant application scenario as seen from the research community. However, new concepts such as Industry 4.0 typically demand different communication patterns: Node-to-node divergecast traffic allows, e.g., a product on the conveyor to directly communicate with the relevant robot. Similarly, multicast traffic allows data to be dispersed across a network, e.g., in a monitoring solution with multiple interested entities. Lastly, scenarios where convergecast applications are terminated at two or more sinks, may be relevant, e.g., for applications where reliability is especially critical.

Furthermore, Industry 4.0 concepts may require more dynamic topology and traffic requirements: As in the manufacturing example with products on a conveyor, the network would need to continuously adapt to nodes moving in the topology. In addition, the traffic load and destinations would be highly dynamic as dictated by the industrial process. As discussed, such requirements might be challenging to achieve with a centralized scheduling approach. However, few proposals have evaluated such applications. Autonomous approaches might be suitable in dynamic topologies, as they don’t introduce any overhead when changing parent—however, their challenges to support heterogeneous traffic has been discussed earlier.

As highlighted throughout Section 2, security is especially crucial in industrial applications. Proposals for schedulers typically do not address security, yet there exist relevant issues such as the security of the signaling, e.g., spoofing of 6P packets. 6TiSCH addresses some of these issues, such as authenticating nodes before they are allowed to join a network. Mitigation against jamming might also be crucial—an adversary able to deduce the schedule could, with high efficiency, jam a network. Such attacks may have severe consequences, especially if they could target specific key timeslots, e.g., those used for 802.15.4 beacons or routing maintenance.

All schedulers will incur overhead for implementing, deploying and maintaining a schedule. In addition, there may also be overhead associated with maintaining state and capturing changes in context and environment. For several of the proposals this seems not to have been investigated. Centralized schedulers need a reliable PCE. In a changing environment, the PCE must also collect information and deploy new schedules. All add overhead and risk that the schedule is out of tune with the changes in the environment. Decentralized schedulers do not require any infrastructure, yet several proposals such as, e.g., OTF, require operators to tune parameters according to the needs of each deployment. The workload required to identify the parameters, and how frequent adjustments are needed, has not been investigated. As discussed in Section 7, the simplicity of autonomous approaches typically poses fewer requirements on the operators, but often with a cost of sub-optimal schedules in terms of, e.g., energy, throughput and band occupancy.

Reproducing and comparing experimental evaluations of schedulers are inherently challenging. Our literature surveying has shown that publications often provide minimal description of the setup, utilize custom testbeds or simulators, and keep implementations closed-source. Furthermore, most evaluations use different settings for traffic patterns, duration, RPL configuration and convergence, software versions, and so on—making comparison inherently difficult. This challenge has been recognized in the community, spurring initiatives such as the IoT Benchmarking consortium (https://iotbench.ethz.ch/ (accessed on 12 December 2021)), OpenBenchmark [150], and others discussed in Section 5.3—yet several tasks remain unresolved.

As argued in [151], a proper understanding of a scheduler requires experiments conducted in testbeds or real-world deployments. Simulators may introduce unrealistic assumptions or oversimplified models, leading to inaccurate results. As discussed in Section 5.3, we found experiments are not uncommon, yet simulation is by far most common tool for evaluation, typically without any other supplement.

## 11. Conclusions

This paper surveys the state of the art of TSCH scheduling targeting the industrial domain, with a total of 76 TSCH schedulers identified, analyzed and evaluated. Each scheduler is categorized according to the generation of the schedule: Collaborative, Autonomous, Centralized, Hybrid, or Static. To gather insight, schedulers are broken down into their components, and their objectives are qualitatively evaluated. The analysis of each scheduler is omitted for brevity and provided as supplementary material.

Collaborative schedulers are divided into local, recursive, and end-to-end according to how requirements and negotiations are dispersed and conducted. This highlights properties such as the simplicity of local collaborative schedulers making them suitable for volatile networks with shifting topology and traffic. Similarly, an end-to-end approach may provide more optimized schedules, yet with only one proposal thus far, more research is needed. Analyzing autonomous schedulers we find they are primarily driven by seminal contributions such as Orchestra, upon which several other proposals are expanding. Crucial properties identified in autonomous scheduling include inherent tackling of faults, in which new links may be added without any overhead at the MAC layer. This is particularly interesting, as other categories of schedulers seldom address fault tolerance. Centralized scheduling is a long-standing and widespread approach, yet unrealistic assumptions such as perfect links are often assumed. Discussions also include the overhead associated with centralized schedulers and their suitability in more stable networks. The few proposals addressing this key issue are highlighted, yet we find this is often tackled by employing collaborative mechanisms—yielding a hybrid scheduler. This trend is also identified with autonomous scheduling, in which shortcomings such as support for heterogeneous traffic are achieved by adding collaborative mechanisms.

A holistic view of the entire research area reveals trends such as the fairly recent introduction and traction of autonomous and hybrid schedulers, the slow adaption of testbed evaluations, and the lack of attention towards traffic patterns beside convergecast. Seminal contributions are identified, such as TASA, DeTAS, OTF, and Orchestra, as well as notable recent proposals such as ALICE, TESLA, and OST.

Lastly, areas of future research and challenges in existing work are identified. Examples of this include further exploration of autonomous, hybrid and end-to-end collaborative approaches, which have received limited attention. Similarly, objectives such as fault tolerance, scalability and band occupancy are rarely addressed. Key challenges include the evaluation of schedulers, where reproducible evaluations and testbed experiments are lacking.

## Figures and Tables

**Table 2:** Properties of collaborative strategies.

Collaborative Strategy	Traffic Requirements	Reservation Protocol	End-to-End Aware	6TiSCH Notation	Example
Local	Local	One-hop		Neighbor-to-neighbor	OTF
Recursive	Multi-hop	One-hop		Neighbor-to-neighbor	DeTAS, DeAMON
End-to-end	Multi-hop	Multi-hop	✓	Hop-by-hop	CFDS

**Table 5:** Components of autonomous TSCH schedulers.

Scheduler	Cell Selection			Cell Amount
	Algorithm	Input		Input
Orchestra [53]	Hash	Node ID		Fixed
ALICE [56]	Hash	Link node IDs, direction, time		Fixed
Escalator [107]	Sequential hash	Node ID, Source ID, hop count		Fixed
Elst et al. [111]	Hash	Node ID		Queue, node ID, rank
Phung et al. [110]	Trial-and-error	N/A		Trial-and-error
BOOST [109]	Odd/even	Rank		Fixed
Layered [108]	Layered hash	Node ID, Source ID, hop count		Fixed

**Table 6:** Evaluation of autonomous TSCH schedulers.

Scheduler	Objectives								
	Det. Latency	Short Latency	Reliability	Fault Tolerance	Scalability	Hetero. Traffic	High Throughput	Energy	Overhead min.
Orchestra [53]			✓	✓					✓
ALICE [56]			✓	✓	✓				✓
Escalator [107]	✓	✓	✓	✓					✓
Elsts et al. [111]			✓	✓		✓	✓		✓
Phung et al. [110]	✓		✓			✓			
BOOST [109]			✓					✓	✓
Layered [108]	✓		✓	✓					✓

**Table 12:** Evaluation of hybrid schedulers.

Scheduler	Objectives								
	Det. Latency	Short Latency	Reliability	Fault Tolerance	Scalability	Hetero. Traffic	High Throughput	Energy	Overhead min.
Fafoutis et al. [142]			✓	✓		✓		✓	✓
PAAS [140]			✓	✓		✓		✓	✓
e-TSCH-Orch [141]			✓	✓		✓			✓
TESLA [144]			✓	✓		✓			✓
OST [145]	✓		✓	✓		✓		✓	✓
MABO-TSCH [57]			✓						
AMUS [143]	✓	✓	✓			✓		✓	
Karaagac et al. [113]	✓	✓	✓		✓	✓		✓	✓
CLS [122]	✓	✓		✓	✓	✓		✓	✓
QSS [123]	✓	✓		✓	✓	✓	✓	✓	✓

## Supplementary Materials

The following are available online at https://www.mdpi.com/article/10.3390/s22010015/s1, Description and evaluation of the 76 surveyed TSCH schedulers.

## References

[1] Gilchrist A. (2016). Industry 4.0: The Industrial Internet of Things.

[2] Hermann M., Pentek T., Otto B. Design Principles for Industrie 4.0 Scenarios. Proceedings of the 2016 49th Hawaii International Conference on System Sciences (HICSS).

[3] International Electrotechnical Commission (2016). Industrial Networks—Wireless Communication Network and Communication Profiles—WirelessHART (IEC 62591:2016).

[4] International Electrotechnical Commission (2014). Industrial Networks—Wireless Communication Network and Communication Profiles-ISA 100.11a (IEC 62734).

[5] International Electrotechnical Commission (2015). Industrial Networks—Wireless Communication Network and Communication Profiles—WIA-PA (IEC 62601:2015).

[6] Shabro M., Ghorashi S.A. (2015). Comparison of IWSN MAC Protocols for IEC 61850 Applications. Int. J. Innov. Res. Electr. Electron. Instrum. Control Eng..

[7] IEEE (2020). IEEE Standard for Low-Rate Wireless Networks. IEEE Std 802.15.4-2020 (Revision of IEEE Std 802.15.4-2015).

[8] IEEE (2012). IEEE Standard for Local and metropolitan area networks—Part 15.4: Low-Rate Wireless Personal Area Networks (LR-WPANs) Amendment 1: MAC sublayer. IEEE Std 802.15.4e-2012 (Amendment to IEEE Std 802.15.4-2011).

[9] Hermeto R.T., Gallais A., Theoleyre F. (2017). Scheduling for IEEE802.15.4-TSCH and slow channel hopping MAC in low power industrial wireless networks: A survey. Comput. Commun..

[10] Kharb S., Singhrova A. (2019). A survey on network formation and scheduling algorithms for time slotted channel hopping in industrial networks. J. Netw. Comput. Appl..

[11] Frotzscher A., Wetzker U., Bauer M., Rentschler M., Beyer M., Elspass S., Klessig H. Requirements and current solutions of wireless communication in industrial automation. Proceedings of the 2014 IEEE International Conference on Communications Workshops (ICC).

[12] Anastasi G., Conti M., Francesco M.D. (2011). A Comprehensive Analysis of the MAC Unreliability Problem in IEEE 802.15.4 Wireless Sensor Networks. IEEE Trans. Ind. Inform..

[13] Luvisotto M., Pang Z., Dzung D. (2016). Ultra High Performance Wireless Control for Critical Applications: Challenges and Directions. IEEE Trans. Ind. Inform..

[14] Grossman E. (2019). Deterministic Networking Use Cases. RFC 8578. https://www.rfc-editor.org/info/rfc8578.

[15] Demir M.O., Pusane A.E., Dartmann G., Ascheid G., Kurt G.K. (2020). A Garden of Cyber Physical Systems: Requirements, Challenges, and Implementation Aspects. IEEE Internet Things Mag..

[16] Palattella M.R., Watteyne T., Wang Q., Muraoka K., Accettura N., Dujovne D., Grieco L.A., Engel T. (2016). On-the-Fly Bandwidth Reservation for 6TiSCH Wireless Industrial Networks. Sens. J. IEEE.

[17] Zhao G. (2011). Wireless sensor networks for industrial process monitoring and control: A survey. Netw. Protoc. Algorithms.

[18] Salam H.A., Khan B.M. (2016). IWSN—Standards, Challenges and Future. IEEE Potentials.

[19] Erman A.T., Incel O.D., Hancke G.P. (2013). Medium Access Control and Routing in Industrial Wireless Sensor Networks. Industrial Wireless Sensor Networks Applications, Protocols, and Standards.

[20] Xia F., Rahim A. (2015). MAC Protocols for Cyber-Physical Systems.

[21] Kumar S.A.A., Ovsthus K., Kristensen L.M. (2014). An Industrial Perspective on Wireless Sensor Networks; A Survey of Requirements, Protocols, and Challenges. IEEE Commun. Surv. Tutor..

[22] Guglielmo D.D., Brienza S., Anastasi G. (2016). IEEE 802.15.4e: A survey. Comput. Commun..

[23] Palattella M., Accettura N., Grieco L., Boggia G., Dohler M., Engel T. (2013). On Optimal Scheduling in Duty-Cycled Industrial IoT Applications Using IEEE802.15.4e TSCH. Sens. J. IEEE.

[24] Rawat P., Singh K.D., Chaouchi H., Bonnin J.M. (2013). Wireless sensor networks: A survey on recent developments and potential synergies. J. Supercomput..

[25] Krishnamurthy L., Adler R., Buonadonna P., Chhabra J., Flanigan M., Kushalnagar N., Nachman L., Yarvis M. (2005). Design and Deployment of Industrial Sensor Networks: Experiences from a Semiconductor Plant and the North Sea. Proceedings of the 3rd International Conference on Embedded Networked Sensor Systems, SenSys ’05.

[26] Iova O., Theoleyre F., Watteyne T., Noel T. (2017). The Love-Hate Relationship between IEEE 802.15.4 and RPL. IEEE Commun. Mag..

[27] Phinney T., Thubert P., Dwars S. (2009). Industrial Routing Requirements in Low-Power and Lossy Networks. RFC 5673. https://www.rfc-editor.org/info/rfc5673.

[28] Zand P., Chatterjea S., Das K., Havinga P. (2012). Wireless Industrial Monitoring and Control Networks: The Journey So Far and the Road Ahead. J. Sens. Actuator Netw..

[29] Bari A., Jiang J., Jaekel A., Hancke G.P. (2013). Fault Tolerant Industrial Wireless Sensor Networks. Industrial Wireless Sensor Networks Applications, Protocols, and Standards.

[30] Somappa A.A.K., Ovsthus K., Kristensen L.M. (2014). Towards a Dual-mode Adaptive {MAC} Protocol (DMA-MAC) for Feedback-based Networked Control Systems. Procedia Comput. Sci..

[31] Akerberg J., Gidlund M., Bjorkman M. Future research challenges in wireless sensor and actuator networks targeting industrial automation. Proceedings of the 2011 9th IEEE International Conference on Industrial Informatics.

[32] Bormann C., Ersue M., Keranen A. (2014). Terminology for Constrained-Node Networks. RFC 7228, RFC Editor. http://www.rfc-editor.org/rfc/rfc7228.txt.

[33] Accettura N., Piro G. Optimal and secure protocols in the IETF 6TiSCH communication stack. Proceedings of the 2014 IEEE 23rd International Symposium on Industrial Electronics (ISIE).

[34] Dujovne D., Watteyne T., Vilajosana X., Thubert P. (2014). 6TiSCH: Deterministic IP-enabled industrial internet (of things). IEEE Commun. Mag..

[35] Scheible G., Dzung D., Endresen J., Frey J.E. (2007). Unplugged but connected [Design and implementation of a truly wireless real-time sensor/actuator interface]. IEEE Ind. Electron. Mag..

[36] Mahmood N.H., Marchenko N., Gidlund M., Popovski P. (2020). Wireless Networks and Industrial IoT: Applications, Challenges and Enablers.

[37] Werb J. (2010). The Technology Behind ISA100.11a User Driven Design.

[38] Gungor V.C., Hancke G.P. (2013). Industrial Wireless Sensor Networks: Applications, Protocols, and Standards.

[39] (2020). Evaluating and Modeling IEEE 802.15.4 TSCH Resilience against Wi-Fi Interference in New-Generation Highly-Dependable Wireless Sensor Networks. Ad Hoc Netw..

[40] Watteyne T., Adjih C., Vilajosana X. Lessons learned from large-scale dense IEEE802.15.4 connectivity traces. Proceedings of the 2015 IEEE International Conference on Automation Science and Engineering (CASE).

[41] Watteyne T., Mehta A., Pister K. (2009). Reliability Through Frequency Diversity: Why Channel Hopping Makes Sense. Proceedings of the 6th ACM Symposium on Performance Evaluation of Wireless Ad Hoc, Sensor, and Ubiquitous Networks, PE-WASUN’09.

[42] Wang Q., Vilajosana X., Watteyne T. (2018). 6TiSCH Operation Sublayer (6top) Protocol (6P). RFC 8480. https://www.rfc-editor.org/info/rfc8480.

[43] Vilajosana X., Watteyne T., Chang T., Vučinić M., Duquennoy S., Thubert P. (2019). IETF 6TiSCH: A Tutorial. IEEE Commun. Surv. Tutor..

[44] Alexander R., Brandt A., Vasseur J., Hui J., Pister K., Thubert P., Levis P., Struik R., Kelsey R., Winter T. (2012). RPL: IPv6 Routing Protocol for Low-Power and Lossy Networks. RFC 6550. https://www.rfc-editor.org/info/rfc6550.

[45] Watteyne T., Handziski V., Vilajosana X., Duquennoy S., Hahm O., Baccelli E., Wolisz A. (2016). Industrial Wireless IP-Based Cyber-Physical Systems. Proc. IEEE.

[46] Thubert P. (2021). An Architecture for IPv6 over the Time-Slotted Channel Hopping Mode of IEEE 802.15.4 (6TiSCH). RFC 9030. https://www.rfc-editor.org/info/rfc9030.

[47] Vilajosana X., Pister K., Watteyne T. (2017). Minimal IPv6 over the TSCH Mode of IEEE 802.15.4e (6TiSCH) Configuration. RFC 8180. https://www.rfc-editor.org/info/rfc8180.

[48] Chang T., Vučinić M., Vilajosana X., Duquennoy S., Dujovne D.R. (2021). 6TiSCH Minimal Scheduling Function (MSF). RFC 9033. https://www.rfc-editor.org/info/rfc9033.

[49] Elsts A., Kim S., Kim H., Kim C. (2020). An Empirical Survey of Autonomous Scheduling Methods for TSCH. IEEE Access.

[50] Awduche D.O., Berger L., Gan D.H., Li D.T., Srinivasan D.V., Swallow G. (2013). RSVP-TE: Extensions to RSVP for LSP Tunnels. RFC 3209. https://www.rfc-editor.org/info/rfc3209.

[51] Palattella M.R., Accettura N., Dohler M., Grieco L.A., Boggia G. Traffic Aware Scheduling Algorithm for reliable low-power multi-hop IEEE 802.15.4e networks. Proceedings of the 2012 IEEE 23rd International Symposium on Personal, Indoor and Mobile Radio Communications—(PIMRC).

[52] Accettura N., Palattella M.R., Boggia G., Grieco L.A., Dohler M. Decentralized Traffic Aware Scheduling for multi-hop Low power Lossy Networks in the Internet of Things. Proceedings of the 2013 IEEE 14th International Symposium on “A World of Wireless, Mobile and Multimedia Networks” (WoWMoM).

[53] Duquennoy S., Al Nahas B., Landsiedel O., Watteyne T. (2015). Orchestra: Robust Mesh Networks Through Autonomously Scheduled TSCH. Proceedings of the 13th ACM Conference on Embedded Networked Sensor Systems, SenSys ’15.

[54] Zorbas D., Kotsiou V., Théoleyre F., Papadopoulos G.Z., Douligeris C. LOST: Localized blacklisting aware scheduling algorithm for IEEE 802.15.4-TSCH networks. Proceedings of the 2018 Wireless Days (WD).

[55] Aijaz A., Raza U. (2017). DeAMON: A Decentralized Adaptive Multi-Hop Scheduling Protocol for 6TiSCH Wireless Networks. IEEE Sens. J..

[56] Kim S., Kim H., Kim C. ALICE: Autonomous Link-based Cell Scheduling for TSCH. Proceedings of the 2019 18th ACM/IEEE International Conference on Information Processing in Sensor Networks (IPSN).

[57] Gomes P.H., Watteyne T., Krishnamachari B. (2017). MABO-TSCH: Multihop and blacklist-based optimized time synchronized channel hopping. Trans. Emerg. Telecommun. Technol..

[58] Accettura N., Vogli E., Palattella M., Grieco L., Boggia G., Dohler M. (2015). Decentralized Traffic Aware Scheduling in 6TiSCH Networks: Design and Experimental Evaluation. IEEE Internet Things J..

[59] Hosni I., Théoleyre F., Hamdi N. Localized scheduling for end-to-end delay constrained Low Power Lossy networks with 6TiSCH. Proceedings of the 2016 IEEE Symposium on Computers and Communication (ISCC).

[60] Hosni I., Théoleyre F. (2017). Self-healing distributed scheduling for end-to-end delay optimization in multihop wireless networks with 6TiSCH. Comput. Commun..

[61] Osterlind F., Dunkels A., Eriksson J., Finne N., Voigt T. Cross-Level Sensor Network Simulation with COOJA. Proceedings of the 2006 31st IEEE Conference on Local Computer Networks.

[62] Municio E., Daneels G., Vučinić M., Latré S., Famaey J., Tanaka Y., Brun K., Muraoka K., Vilajosana X., Watteyne T. (2019). Simulating 6TiSCH networks. Trans. Emerg. Telecommun. Technol..

[63] Watteyne T., Vilajosana X., Kerkez B., Chraim F., Weekly K., Wang Q., Glaser S., Pister K. (2012). OpenWSN: A standards-based low-power wireless development environment. Trans. Emerg. Telecommun. Technol..

[64] Trüb R., Da Forno R., Sigrist L., Mühlebach L., Biri A., Beutel J., Thiele L. FlockLab 2: Multi-Modal Testing and Validation for Wireless IoT. Proceedings of the 3rd Workshop on Benchmarking Cyber-Physical Systems and Internet of Things (CPS-IoTBench 2020).

[65] Adjih C., Baccelli E., Fleury E., Harter G., Mitton N., Noel T., Pissard-Gibollet R., Saint-Marcel F., Schreiner G., Vandaele J. FIT IoT-LAB: A large scale open experimental IoT testbed. Proceedings of the 2015 IEEE 2nd World Forum on Internet of Things (WF-IoT).

[66] (2019). CPS-IoTBench ’19: Proceedings of the 2nd Workshop on Benchmarking Cyber-Physical Systems and Internet of Things.

[67] Boano C.A., Duquennoy S., Förster A., Gnawali O., Jacob R., Kim H.S., Landsiedel O., Marfievici R., Mottola L., Picco G.P. IoTBench: Towards a Benchmark for Low-Power Wireless Networking. Proceedings of the 2018 IEEE Workshop on Benchmarking Cyber-Physical Networks and Systems (CPSBench).

[68] Jacob R., Boano C.A., Raza U., Zimmerling M., Thiele L. (2019). Towards a methodology for experimental evaluation in low-power wireless networking. Proceedings of the 2nd Workshop on Benchmarking Cyber-Physical Systems and Internet of Things.

[69] Kritsis K., Papadopoulos G.Z., Gallais A., Chatzimisios P., Théoleyre F. (2018). A Tutorial on Performance Evaluation and Validation Methodology for Low-Power and Lossy Networks. IEEE Commun. Surv. Tutor..

[70] Baumann D., Mager F., Wetzker U., Thiele L., Zimmerling M., Trimpe S. (2021). Wireless Control for Smart Manufacturing: Recent Approaches and Open Challenges. Proc. IEEE.

[71] Municio E., Latré S. (2016). Decentralized Broadcast-based Scheduling for Dense Multi-hop TSCH Networks. Proceedings of the Workshop on Mobility in the Evolving Internet Architecture, MobiArch ’16.

[72] Morell A., Vilajosana X., Vicario J.L., Watteyne T. (2013). Label switching over IEEE802.15.4e networks. Trans. Emerg. Telecommun. Technol..

[73] Chang T.T.C., Watteyne T., Qin W., Vilajosana X. LLSF: Low Latency Scheduling Function for 6TiSCH Networks. Proceedings of the 12th International Conference on Distributed Computing in Sensor Systems (DCOSS).

[74] Muraoka K., Watteyne T., Accettura N., Vilajosana X., Pister K. (2016). Simple Distributed Scheduling with Collision Detection in TSCH Networks. IEEE Sens. J..

[75] Kim K.T., Kim J. An Energy Efficient Real-Time MAC Protocol. Proceedings of the 2018 International Conference on Information and Communication Technology Convergence (ICTC).

[76] Krueger L., Steenbrink L., Timm-Giel A. Avoiding Local Interference in IEEE 802.15.4 TSCH Networks using a Scheduling Function with Distributed Blacklists. Proceedings of the Mobile Communication—Technologies and Applications; 24. ITG-Symposium.

[77] Lee T.H., Chang L.H., Liu Y.W., Liaw J.J., Chu H.C. (2017). Priority-based scheduling using best channel in 6TiSCH networks. Clust. Comput..

[78] Daneels G., Spinnewyn B., Latré S., Famaey J. (2018). ReSF: Recurrent Low-Latency Scheduling in IEEE 802.15.4e TSCH networks. Ad Hoc Netw..

[79] Fafoutis X., Papadopoulos G.Z., Montavont N., Papadopoulos G.Z. (2018). The Trade-Offs of Cell Over-Provisioning in IEEE 802.15.4 TSCH Networks. Ad-Hoc, Mobile, and Wireless Networks.

[80] Cena G., Scanzio S., Seno L., Valenzano A., Zunino C. Energy-Efficient Link Capacity Overprovisioning In Time Slotted Channel Hopping Networks. Proceedings of the 2020 16th IEEE International Conference on Factory Communication Systems (WFCS).

[81] Kotsiou V., Papadopoulos G.Z., Chatzimisios P., Theoleyre F. (2020). LDSF: Low-Latency Distributed Scheduling Function for Industrial Internet of Things. IEEE Internet Things J..

[82] Hamza T., Kaddoum G. Enhanced Minimal Scheduling Function for IEEE 802.15.4e TSCH Networks. Proceedings of the 2019 IEEE Wireless Communications and Networking Conference (WCNC).

[83] Vergados D.J., Kralevska K., Jiang Y., Michalas A. (2020). Local voting: A new distributed bandwidth reservation algorithm for 6TiSCH networks. Comput. Netw..

[84] Domingo-Prieto M., Chang T., Vilajosana X., Watteyne T. (2016). Distributed PID-Based Scheduling for 6TiSCH Networks. IEEE Commun. Lett..

[85] Jung J., Kim D., Lee T., Kang J., Ahn N., Yi Y. Distributed Slot Scheduling for QoS Guarantee over TSCH-based IoT Networks via Adaptive Parameterization. Proceedings of the 2020 19th ACM/IEEE International Conference on Information Processing in Sensor Networks (IPSN).

[86] Righetti F., Vallati C., Das S.K., Anastasi G. An Experimental Evaluation of the 6top Protocol for Industrial IoT Applications. Proceedings of the 2019 IEEE Symposium on Computers and Communications (ISCC).

[87] Righetti F., Vallati C., Anastasi G., Das S. Performance Evaluation the 6top Protocol and Analysis of its Interplay with Routing. Proceedings of the 2017 IEEE International Conference on Smart Computing (SMARTCOMP).

[88] Juc I., Alphand O., Guizzetti R., Favre M., Duda A. (2017). Stripe: A Distributed Scheduling Protocol for 802.15.4e TSCH Networks.

[89] Yoo D., Chung S., Ha Y. Multipath Scheduling for Energy Balancing and Reliable Transmission over 6TiSCH WSN. Proceedings of the 2019 Eleventh International Conference on Ubiquitous and Future Networks (ICUFN).

[90] Soua R., Minet P., Livolant E. (2015). Wave: A Distributed Scheduling Algorithm for Convergecast in IEEE 802.15.4e Networks (Extended Version).

[91] Duy T.P., Dinh T., Kim Y. (2017). Distributed Cell Selection for Scheduling Function in 6TiSCH Networks. Comput. Stand. Interfaces.

[92] Fahs A.J., Bertolini R., Alphand O., Rousseau F., Altisen K., Devismes S. Collision prevention in distributed 6TiSCH networks. Proceedings of the 2017 IEEE 13th International Conference on Wireless and Mobile Computing, Networking and Communications (WiMob).

[93] Righetti F., Vallati C., Anastasi G., Das S.K. Analysis and Improvement of the On-The-Fly Bandwidth Reservation Algorithm for 6TiSCH. Proceedings of the 2018 IEEE 19th International Symposium on “A World of Wireless, Mobile and Multimedia Networks” (WoWMoM).

[94] Micoli G., Boccadoro P., Valecce G., Petitti A., Colella R., Milella A., Grieco L.A. ASAP: A Decentralized Slot Reservation Policy for Dynamic 6TiSCH Networks in Industrial IoT. Proceedings of the 2019 IEEE International Conference on Communications Workshops (ICC Workshops).

[95] van der Lee T., Exarchakos G., de Groot S.H. Swarm-Based Energy Efficient Scheduling for Wireless Sensor Networks. Proceedings of the 2019 IEEE Conference on Standards for Communications and Networking (CSCN).

[96] Zhang Y., Chen C., Zhu S. An Adaptive Distributed Scheduling Algorithm for IEEE 802.15.4e TSCH Protocol. Proceedings of the 2019 3rd International Symposium on Autonomous Systems (ISAS).

[97] Theoleyre F., Papadopoulos G.Z. (2016). Experimental Validation of a Distributed Self-Configured 6TiSCH with Traffic Isolation in Low Power Lossy Networks. Proceedings of the 19th ACM International Conference on Modeling, Analysis and Simulation of Wireless and Mobile Systems.

[98] Elsts A., Pope J., Fafoutis X., Piechocki R., Oikonomou G. (2019). Instant: A TSCH Schedule for Data Collection from Mobile Nodes. Proceedings of the 2019 International Conference on Embedded Wireless Systems and Networks, EWSN ’19.

[99] Boucetta C., Nour B., Moungla H., Lahlou L. An IoT Scheduling and Interference Mitigation Scheme in TSCH Using Latin Rectangles. Proceedings of the 2019 IEEE Global Communications Conference (GLOBECOM).

[100] Farag H., Grimaldi S., Gidlund M., Österberg P. (2020). REA-6TiSCH: Reliable Emergency-Aware Communication Scheme for 6TiSCH Networks. IEEE Internet Things J..

[101] Tavallaie O., Taheri J., Zomaya A.Y. (2020). Towards Optimizing Time-Slotted Channel Hopping Scheduling on 6TiSCH Networks: Poster Abstract. Proceedings of the 18th Conference on Embedded Networked Sensor Systems, SenSys ’20.

[102] Soua R., Minet P., Livolant E. DiSCA: A distributed scheduling for convergecast in multichannel wireless sensor networks. Proceedings of the 2015 IFIP/IEEE International Symposium on Integrated Network Management (IM).

[103] Ünlü B., Özceylan B., Baykal B. (2019). DIVVY: An Efficient Shared Cell Scheduling Method and Algorithm for 6TiSCH-Based IoT Networks. IEEE Trans. Green Commun. Netw..

[104] Hajian H., Nabi M., Fakouri M., Veisi F. LaDiS: A Low-Latency Distributed Scheduler for Time-Slotted Channel Hopping Networks. Proceedings of the 2019 IEEE Wireless Communications and Networking Conference (WCNC).

[105] Cheng W., Lee I.T.A., Singh N. Time division hashing (TDH): A new scheduling scheme for wireless ad-hoc networks. Proceedings of the International Symposium on Advanced Radio Technologies (ISART).

[106] Halkes G.P., Langendoen K.G., Langendoen K., Voigt T. (2007). Crankshaft: An Energy-Efficient MAC-Protocol for Dense Wireless Sensor Networks. Wireless Sensor Networks, Proceedings of the 4th European Conference, EWSN 2007, Delft, The Netherlands, 29–31 January 2007.

[107] Oh S., Hwang D., Kim K.H., Kim K. (2018). Escalator: An Autonomous Scheduling Scheme for Convergecast in TSCH. Sensors.

[108] Urke A.R., Kure Ø., Øvsthus K. (2020). Layered autonomous TSCH scheduler for minimal band occupancy with bounded latency. Internet Technol. Lett..

[109] Jin Y., Raza U., Sooriyabandara M. BOOST: Bringing Opportunistic ROuting and Effortless-Scheduling to TSCH MAC. Proceedings of the 2018 IEEE Global Communications Conference (GLOBECOM).

[110] Phung K., Huong T.T., Khanh Dung D., Tuong V.X., Pham T., Nguyen T., Steenhaut K. A Scheduler for Time Slotted Channel Hopping Networks supporting QoS Differentiated Services. Proceedings of the 2018 International Conference on Advanced Technologies for Communications (ATC).

[111] Elsts A., Fafoutis X., Pope J., Oikonomou G., Piechocki R., Craddock I. Scheduling High Rate Unpredictable Traffic in IEEE 802.15.4 TSCH Networks. Proceedings of the 13th International Conference on Distributed Computing in Sensor Systems (DCOSS).

[112] Municio E., Spaey K., Latré S. (2018). A distributed density optimized scheduling function for IEEE 802.15.4e TSCH networks. Trans. Emerg. Telecommun. Technol..

[113] Karaagac A., Moerman I., Hoebeke J. (2018). Hybrid Schedule Management in 6TiSCH Networks: The Coexistence of Determinism and Flexibility. IEEE Access.

[114] Finn N., Thubert P., Varga B., Farkas J. (2019). Deterministic Networking Architecture. RFC 8655. https://www.rfc-editor.org/info/rfc8655.

[115] Thubert P., Palattella M.R., Engel T. 6TiSCH centralized scheduling: When SDN meet IoT. Proceedings of the 2015 IEEE Conference on Standards for Communications and Networking (CSCN).

[116] Huynh T., Theoleyre F., Hwang W.J. (2017). On the interest of opportunistic anycast scheduling for wireless low power lossy networks. Comput. Commun..

[117] Matsui T., Nishi H. Time slotted channel hopping scheduling based on the energy consumption of wireless sensor networks. Proceedings of the 2018 IEEE 15th International Workshop on Advanced Motion Control (AMC).

[118] Ojo M., Giordano S., Portaluri G., Adami D., Pagano M. An energy efficient centralized scheduling scheme in TSCH networks. Proceedings of the 2017 IEEE International Conference on Communications Workshops (ICC Workshops).

[119] Gaillard G., Barthel D., Theoleyre F., Valois F. High-reliability scheduling in deterministic wireless multi-hop networks. Proceedings of the 2016 IEEE 27th Annual International Symposium on Personal, Indoor, and Mobile Radio Communications (PIMRC).

[120] Jin Y., Raza U., Aijaz A., Sooriyabandara M., Gormus S. (2018). Content Centric Cross-Layer Scheduling for Industrial IoT Applications Using 6TiSCH. IEEE Access.

[121] Harms O., Landsiedel O. MASTER: Long-Term Stable Routing and Scheduling in Low-Power Wireless Networks. Proceedings of the 2020 16th International Conference on Distributed Computing in Sensor Systems (DCOSS).

[122] Choi K.H., Chung S.H., Galinina O., Balandin S., Koucheryavy Y. (2016). A New Centralized Link Scheduling for 6TiSCH Wireless Industrial Networks. Internet of Things, Smart Spaces, and Next Generation Networks and Systems.

[123] Choi K., Chung S.H. (2017). Enhanced time-slotted channel hopping scheduling with quick setup time for industrial Internet of Things networks. Int. J. Distrib. Sens. Netw..

[124] Wu H., Lee D. Robust QoS Scheduling using alternate path for recovery from link failures in IEEE 802.15.4e. Proceedings of the 2014 Seventh International Conference on Mobile Computing and Ubiquitous Networking (ICMU).

[125] Farias A.A., Dujovne D. A queue-based scheduling algorithm for PCE-enabled Industrial Internet of Things networks. Proceedings of the 2015 Sixth Argentine Conference on Embedded Systems (CASE).

[126] Soua R., Minet P., Livolant E. MODESA: An optimized multichannel slot assignment for raw data convergecast in wireless sensor networks. Proceedings of the 2012 IEEE 31st International Performance Computing and Communications Conference (IPCCC).

[127] Yang D., Xu Y., Wang H., Zheng T., Zhang H., Zhang H., Gidlund M. (2015). Assignment of Segmented Slots Enabling Reliable Real-Time Transmission in Industrial Wireless Sensor Networks. IEEE Trans. Ind. Electron..

[128] Ramachandran G.S., Matthys N., Daniels W., Joosen W., Hughes D. Building Dynamic and Dependable Component-Based Internet-of-Things Applications with Dawn. Proceedings of the 2016 19th International ACM SIGSOFT Symposium on Component-Based Software Engineering (CBSE).

[129] Chen T.S., Kuo S.Y., Kuo C.H. Scheduling for Data Collection in Multi-hop IEEE 802.15.4e TSCH Networks. Proceedings of the 2016 International Conference on Networking and Network Applications (NaNA).

[130] Ojo M., Giordano S. An efficient centralized scheduling algorithm in IEEE 802.15.4e TSCH networks. Proceedings of the 2016 IEEE Conference on Standards for Communications and Networking (CSCN).

[131] Khoufi I., Minet P., Rmili B. Scheduling Transmissions with Latency Constraints in an IEEE 802.15.4e TSCH Network. Proceedings of the 2017 IEEE 86th Vehicular Technology Conference (VTC-Fall).

[132] Nsabagwa M., Muhumuza J., Kasumba R., Otim J.S., Akol R. Minimal Idle-Listen Centralized Scheduling in TSCH Wireless Sensor Networks. Proceedings of the 2018 41st International Conference on Telecommunications and Signal Processing (TSP).

[133] Minet P., Soua Z., Khoufi I. An Adaptive Schedule for TSCH Networks in the Industry 4.0. Proceedings of the 2018 IFIP/IEEE International Conference on Performance Evaluation and Modeling in Wired and Wireless Networks (PEMWN).

[134] Devaja T., Bajovic D., Vukobratovic D., Gardaševič G. Scheduling in 6TiSCH Networks via Max-Product Message-Passing. Proceedings of the IEEE EUROCON 2019—18th International Conference on Smart Technologies.

[135] Shi K., Zhang L., Qi Z., Tong K., Chen H. (2019). Transmission Scheduling of Periodic Real-Time Traffic in IEEE 802.15. 4e TSCH-Based Industrial Mesh Networks. Wirel. Commun. Mob. Comput..

[136] Khorov E., Lyakhov A., Yusupov R. Scheduling of Dedicated and Shared Links for Fast and Reliable Data Delivery in IEEE 802.15.4 TSCH Networks. Proceedings of the 2019 International Conference on Engineering and Telecommunication (EnT).

[137] Brun-Laguna K., Minet P., Tanaka Y. Optimized Scheduling for Time-Critical Industrial IoT. Proceedings of the 2019 IEEE Global Communications Conference (GLOBECOM).

[138] Portaluri G., Giordano S. Gambling on fairness: A fair scheduler for IIoT communications based on the shell game. Proceedings of the 2020 IEEE 25th International Workshop on Computer Aided Modeling and Design of Communication Links and Networks (CAMAD).

[139] Park H., Kim H., Kim K.T., Kim S., Mah P. (2019). Frame-Type-Aware Static Time Slotted Channel Hopping Scheduling Scheme for Large-Scale Smart Metering Networks. IEEE Access.

[140] Jung J., Kim D., Hong J., Kang J., Yi Y. Parameterized slot scheduling for adaptive and autonomous TSCH networks. Proceedings of the IEEE INFOCOM 2018—IEEE Conference on Computer Communications Workshops (INFOCOM WKSHPS).

[141] Rekik S., Baccour N., Jmaiel M., Drira K., Grieco L.A. (2018). Autonomous and traffic-aware scheduling for TSCH networks. Comput. Netw..

[142] Fafoutis X., Elsts A., Oikonomou G., Piechocki R., Craddock I. Adaptive Static Scheduling in IEEE 802.15.4 TSCH Networks. Proceedings of the 2018 IEEE 4rd World Forum on Internet of Things.

[143] Jin Y., Kulkarni P., Wilcox J., Sooriyabandara M. A centralized scheduling algorithm for IEEE 802.15.4e TSCH based industrial low power wireless networks. Proceedings of the 2016 IEEE Wireless Communications and Networking Conference.

[144] Jeong S., Paek J., Kim H., Bahk S. (2019). TESLA: Traffic-Aware Elastic Slotframe Adjustment in TSCH Networks. IEEE Access.

[145] Jeong S., Kim H.S., Paek J., Bahk S. OST: On-Demand TSCH Scheduling with Traffic-Awareness. Proceedings of the IEEE INFOCOM 2020—IEEE Conference on Computer Communications.

[146] Livolant E., Minet P., Watteyne T., Mitton N., Loscri V., Mouradian A. (2016). The Cost of Installing a 6TiSCH Schedule. Ad-Hoc, Mobile, and Wireless Networks, Proceedings of the 15th International Conference, Adhoc-Now 2016, Lille, France, 4–6 July 2016.

[147] Brachmann M., Duquennoy S., Tsiftes N., Voigt T. IEEE 802.15.4 TSCH in Sub-GHz: Design Considerations and Multi-band Support. Proceedings of the 2019 IEEE 44th Conference on Local Computer Networks (LCN).

[148] Rady M., Lampin Q., Barthel D., Watteyne T. (2021). g6TiSCH: Generalized 6TiSCH for Agile Multi-PHY Wireless Networking. IEEE Access.

[149] Rady M., Lampin Q., Barthel D., Watteyne T. (2021). 6DYN: 6TiSCH with Heterogeneous Slot Durations. Sensors.

[150] Vučinić M., Škrbić B., Kočan E., Pejanović-Djurišić M., Watteyne T. OpenBenchmark: Repeatable and Reproducible Internet of Things Experimentation on Testbeds. Proceedings of the IEEE INFOCOM 2019—IEEE Conference on Computer Communications Workshops (INFOCOM WKSHPS).

[151] Imran M., Said A.M., Hasbullah H. A survey of simulators, emulators and testbeds for wireless sensor networks. Proceedings of the 2010 International Symposium on Information Technology.

