# Supplementary material for: A Survey of 802.15.4 TSCH Schedulers for a Standardized Industrial Internet of Things
Source: Sensors (Basel). 2021 Dec 21;22(1):15. doi: 10.3390/s22010015 (PMC8747498; doi:10.3390/s22010015)
Supplement: Supplementary file 1 [file sensors-22-00015-s001.zip › sensors-1486525-supplementary.pdf]

Supplementary Materials: Description of Schedulers in "A Survey of 802.15.4 TSCH Schedulers for a Standardized Industrial Internet of Things"

Andreas Ramstad Urke [1,3,*], Øivind Kure [2] and Knut Øvsthus [3]

1. Collaborative Scheduling

1.1. Completely Fair Distributed Scheduler (CFDS)

CFDS [1] is the only surveyed *hop-by-hop* collaborative scheduler. It employs the Generalized multiprotocol label switching (GMPLS) [2] to forward data along paths through the network. Under this scheme, a node forwards packets based solely on the packet reception timeslot, i.e. without the use of labels or IP addresses. When receiving a packet in a particular cell, the node knows, based on its schedule, which path this packet belongs to, and consequently which cell it should be transmitted on. This technique is part of GMPLS, and can be used for other access technologies by distinguishing e.g. wave-lengths, physical ports, or time-slots as in this case.

To build the paths and schedules, a subset of the resource reservation protocol - traffic engineering (RSVP-TE) [3] is u tilized. This allows nodes to reserve resources by transmitting a RSVP *Path* message, describing the required bandwidth, towards its intended destination (typically the root). A *Resv* message is sent as reply, which when traversing the network, instructs intermediate nodes to reserve cells for this particular path.

Such reservations can be sent at any time during the network operation; thus a heterogeneous traffic scenario may be supported. All reservations expire after a configurable number of slotframes, N_{SF}, allowing for improved energy efficiency as unused cells may be removed - yet, may incur increased overhead. These properties might make the scheduler inherently tolerant to faults and topology changes, however this is not treated.

The selection of cells in CFDS focuses on convergecast applications. Time- and channel-offset are tackled separately: For the time-offset, each child node is ordered according to their demanded bandwidth (this is initially learned through Path messages), and each node is assigned slots matching the smallest demand. This process is then repeated until all demands have been met, ensuring deterministic latency. Selection of cells are done utilizing information in the Resv messages, such that a daisy chain is formed towards the root aiming for short latency. Key to achieving this is the knowledge of a node *level* (level 0 is the bottom-most leaf, root is at the max level). Using this, a node knows where in the slotframe to place cells, e.g. a node at level 0 would start at the very beginning of the slotframe. Following transmissions are then scheduled on every even-numbered timeslot after the initial offset. This ensures a continuous transmission opportunity upwards, which reduces queue utilization and the chance of buffer overflow.

Channel-offsets are randomly selected at each parent node limited by a blacklist signaled in the Resv message - this prevents 2-hop frequency re-use. The selected channel and blacklist is inserted into the Resv message before being forwarded to the child. The blacklist is maintained at each node by monitoring metrics such as packet reception ratio (PRR) and RSSI to estimate potential collisions. If a threshold is reached, the blacklist is updated and a new random selection is made - thus ensuring a minimum link reliability.

1.2. On-the-Fly Bandwidth Reservation (OTF)

OTF [4] is intended for the 6TiSCH architecture, utilizing 6top to conduct the negotiation of cells between nodes. It employs a *bandwidth estimation algorithm*, which estimate the amount of cells needed to accomodate traffic to a neighbor. The authors recommended this algorithm be tailored to the application requirements. Thus, in [4] several strategies are discussed, while evaluating one based on cell utilization from child nodes and own traffic. In the *allocation algorithm*, the bandwidth estimate is compared to the already allocated cells,

and if pre-defined thresholds are reached, cells are added or removed such that sufficient resources are available while still remaining energy efficient. This also allows OTF to support heterogeneous traffic scenarios. The algorithm also incorporate over-provisioning, i.e. scheduling of redundant cells, to increase reliability. This mechanism also decreases the overhead since the number of cell add/delete negotiations are reduced.

Deterministic latency is addressed by making sure sufficient cells are reserved compared to the calculated bandwidth requirements. Cells are however randomly picked in the slotframe by each node, thus there is no aim for short latency. The collaboration in OTF is limited to the negotiation of cells to be allocated. This is done through the 6P protocol, in which the randomly selected cells are proposed and accepted by the neighbor, as long as there are no collision with existing allocations. The scheduler is evaluated mainly in simulations, yet limited to a 50-node setup with no treatment of its scalability. However, as pointed out in other proposals such as DeBraS in Section 1.5, random selection of cells suffer from increased collisions in the neighborhood as the network scale.

The 6TiSCH Scheduling Function Zero (SF0) is based on OTF, with negligble differences in later revisions [1]. This makes OTF especially relevant because several schedulers utilize SF0 for comparison, use it for parts of their proposals, or propose enhancements to it. These schedulers are presented in the following nine sections.

1.3. Low Latency Scheduling Function (LLSF)

LLSF [6] augments the cell selection mechanism in SF0. In LLSF, when a child node schedules towards a parents, it selects the closest available cell to the right (in the slotframe) of the reception slot from its own child. This way the cells are daisy-chained from the leaf towards the sink, drastically reducing latency.

1.4. Simple Distributed Scheduling with Collision Detection

In [7], Muraoka et al. expand on OTF by adding more sophisticated housekeeping mechanisms to optimize *which* cells are utilized: The main idea is for each node to individually monitor the performance of allocated cells, and when necessary, negotiate for new randomly selected cells. This increases reliability and improve scalability as nodes are more likely to eventually find satisfying cells as the number of nodes increases.

They add two complementary mechanisms which both run autonomously on each node: With *tx-housekeeping*, a node monitors its tx-cells Packet Delivery Ratio (PDR), i.e. the ratio of received acknowledgments to transmissions attempts. If a cell has a lower PDR than other comparable cells, the node will decide to negotiate for a new cell. In *rx-housekeeping*, a node is cognizant of packets from nodes other than the expected neighbor in its rx-cells. Receiving such a packet would indicate a scheduling collision, and the node will trigger a re-allocation.

1.5. Decentralized Broadcast-Based Scheduling for Dense Multi-Hop TSCH Networks (DeBraS)

The DeBraS algorithm [8] is applied on top of OTF and aims at dense multi-hop networks with the main idea to improve cell selection by making nodes aware of their neighborhood. It was extended in [9] with, amongst other, two new modes of initial network configuration: *DeBraS-Aloha* and *DeBraS-TDMA*. It was also re-defined to fit the 6TiSCH paradigm including utilization of the 6top Protocol, and lastly it was extensively evaluated and compared with SF0 [2].

If nodes in a multi-hop networks randomly select cells, such as in OTF, the probability of collisions increases as the network becomes denser. DeBraS therefore suggest having nodes broadcast their current schedule in shared slots for all nodes in the neighborhood to receive. Using this information, each node can avoid already allocated cells when

[1] In earlier drafts of SF0, the bandwidth estimation algorithm relied on bandwidth requests from neighboring nodes. However since the third revision [5] this has been shifted to cell utilization statistics as in OTF [4]

[2] [9] actually references SFX [10] which was the final name of SF0. SFX was short-lived and no significant changes were made before being abandoned.

expanding their own schedule through negotiations with neighbors. This results in fewer collisions, especially in dense large-scale topologies. As shown by the authors, this increases reliability while allowing the network to scale. The overhead introduced by the broadcasts are however significant, causing DeBraS to also display an increased energy consumption in the authors evaluations.

1.6. Enhanced Scheduling Function Zero (E-SF0)

In [11], the authors expand on SF0 and propose the Enhanced SF0 (E-SF0). Similar to DeBraS, they focus on *which* cells are utilized:

To select timeslot-offset, each node divides it slotframe into portions and monitor the density of allocated cells in each portion. When a node needs to schedule additional cells to a neighbor, it uses an *enhanced 6P protocol* to send its list of densities for each portion. The receiving node uses its own list of densities along with the received list, to select the portion with the lowest average density from which cells will be allocated.

To select channel-offset, a node first learns the channels utilized by its RPL parent. It then randomly selects an offset from the remaining available, ensuring a minimum distance of two-hops before re-using channel-offsets. By combining these techniques, collisions are reduced, and the authors point out how this increases energy efficiency and reliability. Similar to DeBraS, this approach avoids the scaling problem where it becomes harder to find available cells as the network scale. In addition, the authors show how selecting cells with less contention reduces the overhead from signaling as fewer re-allocations are needed.

1.7. Collision Prevention in Distributed 6TiSCH Networks

In [12], Fahs et al. propose a mechanism to prevent collisions among cells negotiated between neighbors using the 6top Protocol (6P). This mechanism is applied on top of OTF, and this "OTF + collision avoidance mechanism" is evaluated through simulations. The main idea is to enable nodes to overhear 6P transactions between other nodes in their neighborhood. Doing this, each node is able to maintain a *avoid table* of cells which are already utilized and should be avoided in its own subsequent negotiations.

1.8. Enhanced-OTF (E-OTF)

OTF is analyzed in [13], and the authors argue it is unsuitable for industrial use-cases due to its poor handling of disruptive events such as TSCH de-synchronization, 6P negotiation failures, and RPL parent changes. They propose E-OTF, where link ETX is included in the bandwidth estimation algorithm, thus allocating more cells to nodes with poor link qualities.

In addition, a *bonus mechanism* is added, where nodes with queue sizes beyond a certain threshold will aggressively allocate additional cells - this to recover faster from disruptive events, e.g. during a parent change. Simulations show these additions improve reliability and latency, at a small cost of increased radio duty cycle.

1.9. Decentralized Slot Reservation Policy for Dynamic 6TiSCH Networks (ASAP)

ASAP [14] operates similar to SF0 yet with some key differences: It is conservative when deallocating cells to avoid the scheduler reacting to a temporary outage. This reduces the overhead since some scheduling operations may be omitted, yet would inevitable increase the energy consumption from e.g. cells towards unreachable nodes. Further it does not use a bandwidth estimation algorithm as in SF0, yet immediately negotiate for a fixed amount of cells through their proprietary protocol. This accommodates the evaluated request-response traffic scenario, yet diminish support for further heterogeneous traffic, and decreases reliability since ETX or similar is not taken into account. It addresses fault tolerance by adding shared cells which are utilized as fallback if a dedicated link fails.

1.10. TRaffic-Aware Energy Efficient (TREE) Scheduling

TREE [15] operates similar to SF0, yet the scheduler decisions are inspired by the field of biological swarm behavior. Each node maintain two variables:

Φ is maintained per neighbor and indicate the traffic intensity, i.e. it increases as packets are added to the queue, and otherwise decreases over time. When passing a configured threshold, additional cells are added through a regular 6P negotiation. The cells are selected at random, thus there is no optimization for short latency.

Ψ is maintained per cell and indicates the cell quality and the cell utilization. Thus it is increased when a packet is successfully transmitted, and reduced by retransmissions and idling. This ensures energy efficiency by removing any unused or poorly performing cells.

By monitoring queues, it allows TREE to support heterogeneous traffic patterns and intensities, and thus allow for deterministic latency. The amount of cells are also adapted to the links' ETX, increasing the scheduler reliability. Scalability, overhead, fault tolerance and throughput is not specifically addressed. TREE's performance relies heavily on parameters for the calculation of Φ and Ψ. These must be set by network operators a priori which may be challenging.

1.11. Distributed PID-Based Scheduling for 6TiSCH Networks

Departing from the above string of OTF/SF0-related schedulers, Domingo-Prieto et al. [16] employs the "well-known industrial control paradigm referred as proportional, integral, and derivative (PID) control". It is typically used for stabilizing industrial control loops, i.e. adjusting process variables to minimize deviance from a given target. The authors argue a schedule can be controlled by such an algorithm, utilizing it to decide how many cells should be added or removed. This as opposed to the simpler threshold-based algorithm used in OTF. The implementation of the schedule is deferred to 6top and the 6P protocol, which also handles the selection of which cell to schedule.

In their proposal the controller optimize for queue size and unused slots - thus aiming for deterministic latency and energy. The PID algorithm is configured such that it is more aggressive the further away the current state is from the target. This is shown to be key for bursty heterogeneous traffic patterns, yet it also reduces overhead as unnecessary schedule adjustments are avoided when being close to target.

Lastly, reliability is increased by having the controller monitor the PDR of the randomly selected cells. If found to be below a given threshold, the cell is relocated through a new negotiation using 6P. Further handling of faults are not treated, and the evaluation in [16] is limited to small networks, with the largest scenario being a 7-node daisy-chain.

1.12. Adaptive Distributed Scheduling Algorithm

The scheduler proposed by Zhang et al. [17] shares most of its traits with [16] as both utilize a PID algorithm with the same input to decide when and how many cells to schedule. However, Zhang et al. strengthen the reliability by adding an adaptive channel whitelisting and selection: Firstly, nodes measure the noise level in each channel and generate a global whitelist for the entire network to follow. This list is updated over a long timeframe in the order of "tens of minutes". How nodes agree upon and distribute an updated list is not specified. Secondly, each node decide which channel within the whitelist they want to utilize in their schedule. This decision is taken per slotframe. Game theory is applied to calculate which channel to select, utilizing interference in the utility function. It is assumed neighbors will measure the same interference and thus agree on the channel selected.

1.13. Local Scheduling Function (SF_{loc})

SF_{loc} is proposed in [18], where nodes use a local information to decide when, and how many, cells to insert/remove [3]. This information includes queue size, currently allocated

[3] The rationale behind SF_{loc} came from early drafts of SF0 where nodes estimated bandwidth based on requirements sent from neighbors (see Section 1.2). As opposed to this, Theoleyre et al. proposed a SF based on *local* information only.

cells, and their Estimated Transmission Count (ETX). Cells are then scheduled using the 6top protocol (6P) such that the estimated number of packets which can be sent in one slotframe match the queue size, thus aiming for deterministic latency. Note that ETX is also part of the evaluation, thus taking poorly performing cells into account when building the schedule - leading to increased reliability. Further, a timeout mechanism is applied to each cell such that unused cells are removed, thus improving energy efficiency - note however that this is, alongside the overhead introduced by 6P, not evaluated. This mechanism is similar in strategy to SF0 which monitors overall cell utilization to a neighbor and adapts the number of cells accordingly.

Reservations are done in tracks, where a set of cells from source to destination is associated to e.g. a particular application on a node. Thus the process described above is executed per track. This isolates each flow of traffic and ensures support for heterogeneous applications. The scalability of this approach is not evaluated, yet the authors note it becomes harder to find available cells as the topology grows, as well as increased failure of negotiations due to collisions.

Lastly, random vs. contiguous cell selection strategies are explored, where the latter aims to schedule cells back-to-back towards the sink. As in LLSF (Section 1.3, this is done by having each node, after reserving cells for a track from its child, select the closest available cells when scheduling onward to its parent. This optimizes for short latency, and results confirm this e ffect. Note that the authors envision heterogeneous applications for their proposal, however only a static homogeneous scenario is evaluated.

1.14. Localized Scheduling for End-to-End Delay Constrained Low Power Lossy Networks with 6TiSCH

The main idea by Hosni et al. in [19–21] is the concept of *stratum*, i.e. dividing the slotframe into different blocks or layers, and assigning each block to be utilized by nodes in a certain rank in the routing tree. Nodes at higher ranks will re-use the blocks in the same fashion, i.e. rank five would utilize the first. Thus the number of blocks needs to be set sufficiently high such that interference is avoided when re-using.

SF_{loc} is re-used to decide when and how many cells to allocate, yet *which cells* two neighbors select are now bounded by the rank and its corresponding block: When negotiating with a neighbor to reserve a cell, the node must select randomly from within its designated block. This scheme allows the schedule to be optimized for short latency in upstream traffic as the blocks are arranged sequentially towards the sink.

The concept of 6TiSCH tracks is adopted similarly to SF_{loc}, such that each cell reservation is associated with an application - thus aiming for deterministic latency. However, a necessity for this to perform is the setting of block sizes: To mitigate the funneling effect, the block closest to the sink is the largest (the authors propose halving the block size for each increase in rank).

By re-using SF_{loc}, the number of cells reserved are adjusted according to the queue size, this optimizes for energy and accommodates heterogeneous scenarios. ETX is also used as input, thus aiming for improved reliability. There are no dedicated mechanisms targeting fault tolerance or scalability, and experiments done in the Grenoble FIT IoT-Lab testbed [4] shows slightly reduced reliability for large-scale topologies. This is explained by the increased probability for collisions when nodes randomly select cells confined within each block.

1.15. Priority-Based Scheduling Using Best Channel (P-SBC)

The main idea in P-SBC [22] stems from having each node maintain PDR statistics for each channel using their proposed *Smooth PDR estimate* algorithm which adds some hysteresis to PDR measurements. Using this notion of PDR, the nodes improve their channel hopping scheme - the details on how this is done are not provided, but some sort

[4] https://www.iot-lab.info

of black-listing of worst channels may be assumed. The authors show through simulation how this increases the overall reliability compared to random selection of channels.

In addition, the proposal accommodate heterogeneous applications by including a concept of *emergency packets* (EP). When an EP is received from upper layers, a node will set an EP-flag in the current packet being transmitted to the neighbor - this signals the next transmission will be an emergency packet. The EP is then moved to the front of the queue, ensuring shortest latency possible. To increase reliability, both nodes will not use the channel dictated in the schedule for this timeslot, but switch to the *best channel*. This is maintained by each node for this purpose, and is the channel with the highest PDR.

These two mechanisms are the sole focus of the proposal, and other aspects such as the number of cells to allocate, or when to add cells are not described - thus the remaining objectives are left open.

1.16. Stripe: A Distributed Scheduling Protocol for 802.15.4e TSCH Networks

Stripe [23] assumes each nodes have pre-existing dedicated links to "some or all" of their neighbors. The authors suggest using an autonomous approach or a neighbor discovery protocol to achieve this. In short, Stripe aims to re-organize these reservations to minimize latency, and to add additional cells to accommodate for funneling effects - thus aiming for deterministic latency.

The scheduling is divided into two phases: *Relocation* and *reinforcement*. The relocation phase is initiated by the root node sending a *Stripe Request* to each of its child nodes, suggesting new positions for the pre-allocated cells. The children respond with a *Stripe ACK*, and next sends requests to their children - and so on, until the entire tree is covered. However, whereas the root suggests a random position for the initial cells, regular nodes propose daisy-chained positions which minimize latency towards the root - this sequence of cells are denoted as *stripes*. Note that a Stripe ACK is not a separate packet, but is sent instead of a regular TSCH packet acknowledgment. This reduces overhead as a complete negotiation can be done in one cell, as opposed to the 2- or 3-step 6top transactions.

In the reinforcement phase, which is running in parallel, each parent aims to allocate additional cells to accommodate traffic which it must forward on behalf of its children. This is done using an exchange of *Stripe confirm* and *Stripe reply* messages. The number of additional cells is simply set equal to the number of children, thus a homogeneous traffic scenario is assumed. Fault tolerance and scalability is not treated in [23], there is also no mention of housekeeping or similar mechanisms targeting energy efficiency, and lastly no dedicated mechanism such as over-provisioning to increase reliability.

1.17. Scheduling Function with Soft Blacklisting (SFSB)

SFSB [24] focuses solely on reliability, assuming nodes which are able to continuously monitor all channels. The number of cells allocated are fixed, while the selection is based on channel properties: Nodes maintain a blacklist of channels experiencing interference. This list is continuously shared with 1-hop neighbors through 6P protocol signal messages.

Whenever the schedule dictates to use a cell in a blacklisted channel, the node will either skip this cell (simple mode), or skew the channel-offset of the cell with a pre-defined offset (extended mode). Since blacklists are synchronized between neighbors they will agree on when and which channel such skewed transmissions will occur. Through simulations, the authors find that simple mode may reduce capacity to such a level that it impacts performance, while extended mode is shown to be beneficial in heavy interference scenarios.

1.18. Multipath Scheduling for Energy Balancing and Reliability

Yoo et al. focuses on multi-path scheduling in [25] where they propose allocating cells towards multiple parents. This is done to improve fault tolerance, as well as increase network lifetime by load balancing the energy consumption. All nodes maintain and exchange statistics on the number of packets transmitted and received. This, as well as

measured ETX, is used by child nodes to learn how much energy their candidate parents have consumed. Based on this, nodes may load balance allocations such that lifetime is maximized. This is the only scheduler with such considerations. Reservations are done through the 6P protocol, yet [25] focus on load balancing and does not treat how many cells to allocate, making further analysis difficult.

1.19. Instant: A TSCH Schedule for Data Collection from Mobile Nodes

Instant [26] targets the healthcare sector with wearable TSCH nodes transmitting large amounts of data. A home is equipped with multiple "access points" consisting of TSCH + RPL nodes forming a wireless backbone to receive and forward data from the wearable devices. Instant focuses on the scheduling between these wearables and the access points.

Every slotframe includes a fixed set of probing cells. In these, wearable nodes may anycast a probing packet which indicate they want to be scheduled. Upon reception of a probe, access points adds the node to its list of active nodes and randomly select one node which should get allocated. When receiving a probe from the selected node, it piggybacks in the ACK, a number indicating how many of the following slotframes is allocated to the node. This random selection might cause e.g. unfair distribution of resources - an issue which the authors defer to future work. The resulting allocation-procedure is lightweight (compared to e.g. 6P), yet does require nodes to periodically transmit probing packets. The authors do however remark that many existing wearable applications already rely on periodic broadcast which thus could be exploited by Instant with minimal added overhead.

The number of slotframes allocated are adapted according to the level of mobility - if the neighborhood has remained similar for a period, a larger number is selected to improve throughput. The authors do however note there are scalability issues when the numbers of nodes grow.

If an access point stops receiving probes from a node, it is removed from the list of active nodes. This allows Instant to adapt to the heterogeneous traffic pattern caused by the mobile nodes. Further if data is not received from a node during the allocated slotframe, the remaining slotframes are deallocated to improve energy efficiency and throughput.

1.20. Enhanced Minimal Scheduling Function for IEEE 802.15.4e TSCH Networks (EMSF)

EMSF [27] expands on the then unfinished 6TiSCH Minimal Scheduling Function (MSF). It aims to reduce overhead from 6P by predicting the scheduling needs in an event-triggered convergecast application: Each nodes monitors the average traffic it generates and forwards. By assuming the traffic generated follows a Poisson distribution, the node may calculate the future expected traffic and schedule cells accordingly. Through simulations with OpenWSN, the authors show how this significantly reduces the amount of 6P negotiations compared to MSF, especially as the number of nodes in the network increases. There is however no evaluation of the energy cost of presumable a higher amount of cells, nor is it clear if retransmissions are taken into account when calculating the average traffic.

1.21. Scheduling and Interference Mitigation Scheme Using Latin Rectangles (SIM)

SIM's [28] main idea is to use Latin Rectangles when selecting cells. This with a aim to reduce collisions and thus improve reliability, scalability, and energy consumption. A Latin rectangle is a nxm matrix where m symbols are assigned to each cells such that no symbol occurs more than once in any row or column. A symbol in this case would be a communication link, while rows would be channels and columns would be timeslots. Under this ruleset, collisions could not occur.

Thus, rather than randomly selecting cells when allocating, a node will first calculate a Latin Rectangle based on a knowledge of neighborhood allocations. The details on how a should learn about these allocation are not provided - one option is overhearing, as proposed by Fahs et al. in Section 1.7. Regardless, by selecting in the Latin Rectangle, contention with neighboring nodes is avoided. However, collisions may still occur if nodes outside of the neighborhood are within interference range. Simulations show how

scheduling collisions are reduced compared to a random strategy such as in SF0. The amount of cells to allocate are calculated based on each nodes offered traffic such that determinstic latency is possible even with heterogeneous traffic.

1.22. Reliable Emergency-Aware Communication Scheme (REA-6TiSCH)

REA-6TiSCH [29] focuses on scheduling for spurious emergency traffic (similar to P-SBC in Section 1.15) through a network with an existing schedule. They propose two mechanisms: First, by jamming in the Clear Channel Assessments (CCA) part of a timeslot, a node may hijack cells from other in order to transmit emergency traffic. This allows any allocated cells to immediately be re-purposed for minimal latency. Secondly, to improve reliability, the number of retransmissions attempts is dynamically calculated at each node. This to avoid the packet being needlessly dropped if there are still time left before the deadline of delivery. This is calculated by having packets piggy-back the time it has been in transmit, the number of hops to the destination, and a packet delivery deadline.

1.23. Local Voting

The Local Voting scheduler [30] operates similar to OTF, however, when deciding the number of cells it tries to balance the *load*, defined as the traffic-to-allocated-cells ratio: Every node in a neighborhood is aware of its neighbors queue size. Together with it's own queue it calculates the appropriate load and assigns cells via 6P accordingly. The aim is for all traffic to be accommodated, while fairly distributing the resources according to load. Nodes with less traffic will thus receive less resources, and vice versa, which reduces energy consumption and latency. How information should be broadcasted in the neighborhood is not described.

1.24. Optimized Adaptive TSCH Scheduling Function (OA-TSCH)

OA-TSCH is briefly described in poster abstract [31]. It aims to dynamically adjust the number of cells allocated according to the queue size. The deciding of channel offset is done separately by having TSCH coordinators broadcast the channels to be used such that all nodes on one depth in one sub-tree uses the same channel. This is presumably to avoid collisions within the sub-tree. Timeslots are allocated by parents for its children by receiving their request based on queue size, and finding the optimal value to allocate. Similar to DeTAS in Section 1.25, this value must be less than the parents own amount of nodes to avoid congestion. This problem is treated as a convex optimization problem for which the authors suggest a solution. With the short description in [31], it is not possible to further evaluate the scheduler.

1.25. Decentralized Traffic Aware Scheduling (DeTAS)

DeTAS is the first *recursive* collaborative scheduler surveyed. It was introduced in [32] yet extensively expanded and further evaluated in [33]. It is one of the earlier proposals of collaborative schedulers, and is frequently referred to in literature. Its main goals are short and deterministic latency with minimal overhead. The root initiate the schedule generation after receiving the *global packet number* Q_i from each child, i.e. the amount of packets generated per slotframe in the sub-tree. Initiating scheduling at the root allows for daisy-chaining of cells to minimize latency, while knowing the traffic requirement allows for allocating sufficient cells to ensure deterministic latency. In addition, it allows the root to calculate and set a minimal slotframe length optimized for the traffic requirements signaled in Q_i.

Starting at the root, a number of rx cells matching Q_i for each sub-tree is allocated and signaled to the respective child. Interestingly, this allocation is done for every other timeslot, e.g. at offset 1, 3, 5, etc. At the child, this triggers a corresponding allocation of tx cells, and a reservation of rx cells at offset 2, 4, 6, etc. towards its own child - again with an amount matching the child Q_i. This ensures rx and tx cells alternates towards the

root which reduces queue utilization and the chance for buffer overflow. Together with over-provisioning cells to account for retransmissions, this increases reliability.

The channel offset of these cells is set according to the node rank. Spatial reuse is employed at every W rank, where W is the number of channels available. This allows for a trade-off between band occupancy and interference which is shown through simple experiments in [33]. The performance in real-life deployments are however not known. The utilization of rank also minimizes overhead as it is already learned via the routing protocol.

Signaling between nodes are done utilizing a proprietary protocol (even though DeTAS in [33] targets 6TiSCH, the 6P protocol was not yet drafted). Through experiments they show how DeTAS exhibits the same performance as the network size increases - this can probably be attributed to the collision-free nature of the schedule. Their protocol includes signaling to allow for re-scheduling to occur when traffic requirements are changed or topology altered, e.g. due to node faults. However, this is never evaluated, and both papers assume a static homogeneous topology and traffic scenario.

1.26. Wave

Wave is introduced in [34] and [35]. It focuses on convergecast applications and its main idea is building the schedule in a series of waves initiated by the root. It assumes that any node know of all its *conflicting nodes*, i.e. nodes where collision may occur if they transmitted simultaneously. In addition, nodes must know the traffic demands of all these conflicting nodes.

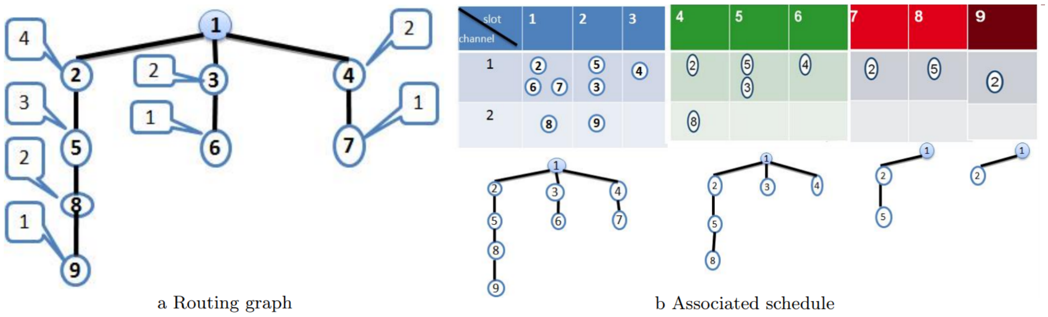

a Routing graph b Associated schedule

Figure S1. Wave example schedule [34].

The root initiates the first wave by transmitting a *Start* message to its children. They then inspect its set of conflicting nodes and their traffic demands: The node with the highest traffic demand allocates a cell for itself, and announces this to the other conflicting nodes in an *Assign* message. The cell selected is the first possible where both node and parent is available, and the first channel which does not conflict with other scheduled links. This process is repeated until all nodes have scheduled one cell each, at which point the first wave is completed. An example of the process can be seen in Figure S1, where each color represents one wave (blue being the first), and the number in each bubble denotes the accumulated traffic demand for the node.

To execute the next wave, nodes need to know how many timeslots the first wave utilized, and for how many waves each allocation should be repeated. To this end, nodes transmit its local information towards root in *Notify* messages, which in turn broadcast the accumulated information to the entire network through *Repeat* messages. Using this, nodes are able to locally calculate the remaining waves by simply repeating allocations from the first wave until the traffic demand is met. The result is a compact slotframe with deterministic latency, which through simulations is shown to be close to an optimal length. This also aims for short latency as it will result in a daisy-chaining of cells towards the root.

It should be noted that the set of conflicting nodes are derived from the network topology and not physical measurements. In real-world deployments this does not guarantee absence of interference. As Wave is evaluated in simulations only, a test-bed experiment

may be warranted to explore this aspect. Wave does however incorporates support for retransmissions to increase reliability by allowing nodes to utilize cells beyond their last designated wave. To accommodate this, all parents are required to listen in the next wave if the child transmitted in the previous wave. This can also be used to support heterogeneous traffic demands. Such an allowance is dependent on the structure of the schedule, and in cases where it is not possible the Wave algorithm above must be re-built. A similar mechanism is described to handle faults: Wave can detect and in certain scenarios avoid re-scheduling for e.g. topology changes.

1.27. Distributed Scheduling for Convergecast (DiSCA)

The authors of Wave later proposed DiSCA [36]. It shares most of its properties and operation with Wave, yet is aimed to be more efficient in its allocation, i.e. minimizing the slotframe size. This is done by allowing the allocations done in one iteration (corresponding to waves in Wave) partly overlap with the previous one, if collision-free operation can be guaranteed. DiSCA does not address, as Wave does, its handling of faults, topology changes, or other disruptive events.

1.28. Decentralized Adaptive Multi-Hop Scheduling Protocol (DeAMON)

The generation of a schedule in DeAMON [37] is always started at the leafs, which is used to ensure that slots are assigned sequentially from the leafs to towards the sink - aiming for the shorter latency. As with DeTAS and Wave, the process is triggered by the root transmitting a *Build command*, which is propagated until it reaches the leaf nodes. This also includes coordinates for dedicated cells to be utilized for signaling between each parent and child. After receiving the build command, the leaf nodes propose cells to fulfill its own traffic requirement to its parent node. The cells selected are the earliest possible in the slotframe, and ones which have not been overheard to be in use - similar to the mechanism suggested by Fahs et al. in Section 1.7. If the parent node also has not received or overheard other requests for the same cells, it accepts the allocation from its child and both nodes updates their schedule. By employing overhearing, the possibility of colliding cells is greatly reduced, and reliability is increased. DeAMON additionally optimizes for parallel transmissions, i.e. spatial re-use of cells, by having nodes embed additional information on their neighborhood schedule in request to parents. Utilizing this information from all their children, parents gets an enhanced view of the topology and can suggest cells which increase parallelization, and consequently reduces the necessary slotframe length.

This process is then repeated towards the sink, where each node utilizes the traffic requirements of its children as well as any self-generated traffic when deciding on the number of cells to reserve - thus aiming for a deterministic latency and accommodating heterogeneous traffic. Note the different approaches to daisy-chain cells from leaf to root: Whereas Wave include all nodes in each scheduling wave, DeAMON starts allocations at the leaf, working upward. DeTAS employs a third approach with the root allocating sets of cells which are distributed downward leaving the leftmost piece to leafs. The authors of DeAMON highlight an objective for minimal overhead, and show how their procedure of building a schedule - starting at leafs with a Build command - is more efficient compared to DeTAS and Wave.

To ensure reliability and deterministic latency, DeAMON introduce the concept of *backup slots*, which are over-provisioned cells to accommodate MAC layer retransmissions. The number of backup slots are determined by the node rank, traffic load, and ETX to its parent. Lastly the OTF algorithm is employed to monitor and dynamically adjust the number of slots. This is done to ensure efficient usage of the over-provisioning resources and thus improve energy efficiency.

In cases of node or link failure, the authors propose mechanisms to avoid a complete re-building of the schedule. This requires a child to inform its new parent of its allocated slots, its old parent address, and the cause of the switch. Depending on the topology

location of the old parent, and if it is still active or not, the authors describe how all scenarios can be tackled by negotiation between child and the new parent, using same principles as in the build-phase.

1.29. Recurrent Low-Latency Scheduling (ReSF)

ReSF [38] exploits the fact that many monitoring applications senses and sends data at a longer period than a slotframe. Its main idea is thus to accommodate scheduling of cells which does not occur in every slotframe. The authors point out this improves energy efficiency due to reduction of idle listening in unused cells. In addition, overhead is reduced since other schedulers often will de-allocate cells if the traffic interval is long.

In ReSF, the application signals the scheduler with traffic characteristics such as starting point (the first occurrence of data ready to be sent) and interval. For each flow, a path from source to destination with *recurring cells* (cells not activated in every slotframe), is generated. This path is implemented by utilizing an extended version of the 6P protocol including additional fields such as the traffic starting point in ASN (Absolute Slot Number), and interval. Using this, a node on the path reserves cells as close to the starting point as possible, and matches their recurrence with the interval. The starting point is then updated to match the reserved cells, and the updated request is utilized in the next hop towards the destination. Thus a *recurrent path* with an aim for deterministic and minimal latency is created. Since the application can at any point signal additional or altered requirements, support for heterogeneous traffic is achieved.

To increase reliability, over-provisioning is applied at every hop by scheduling a number of cells equal to the ceiled ETX (e.g. if ETX is 1.2, 2 cells are allocated). In addition, when application requirements overlap (e.g. similar starting point), a *collision rate* is considered when selecting which cells to allocate: Each node calculate the contention level of each candidate cell as seen from their own schedule, resulting in a cell collision rate. This is utilized when negotiating allocations through the 6top protocol: Cells with the lowest collision rate from the transmitter point-of-view is proposed, while the receiver subsequently selects the cells with lowest rate from its point-of-view.

Lastly, deterministic latency and reliability is strengthened by introducing a housekeeping mechanism which monitors the queue: If a threshold is reached, additional cells are allocated to ensure no packets are lost due to overflow. The authors show through simulations how these mechanisms allow ReSF to scale favorably in comparison to LLSF (Section 1.3). The scheduler reaction and handling of faults are not treated.

1.30. Localized Blacklisting Aware Scheduling (LOST)

LOST [39] employs a range of mechanisms such as overhearing, dynamic over-provisioning, channel blacklisting, etc. to achieve high reliability and deterministic latency. The scheduling process is divided into several phases which is executed multiple times: In the first phase each node signals its intended amount of generated traffic q as well as its rank to their neighbors. This information is used to 1) Decide which nodes starts the *timeslot allocation* first - lower rank (closer to root) and more traffic is prioritized to avoid queue overflows, and 2) Learn how many slots each node requires such that heterogeneous traffic is supported. In the slot allocation phase, each prioritized node allocates slots to its children according to the received information. The child then reduces its q according to the reserved slots, while the parent increases its q to reflect the increased anticipated traffic. This ensures sufficient slots are allocated upstream, thus aiming for deterministic latency. These phases are repeated until all nodes have a q of zero.

Next the channel offset is decided by having the leafs inspect the reservations made in their neighborhood. If any timeslots overlap with its own reservations, it will select the next available channel offset and signal this to its parent. When the parent has received a channel-offset from all its child's, it can repeat this process towards its own parent.

To increase reliability, over-provisioning is employed by having nodes allocate more timeslots than indicated by q. The amount of over-provisioning is set according to the

measured Packet Error Rate for each link, and is dynamically updated according to cell utilization - such as to optimize for energy. In addition, LOST alters the channel hopping sequence by employing a blacklisting mechanism: Each parent and child maintain a local blacklist of the poorest performing channels, and avoid these when executing their channel hopping. Since this list needs to be synchronized among the two nodes, it is assumed to be piggybacked on data or acknowledgment packets.

The authors argue LOST exhibits low overhead due to only 1-hop information is being exchanged. They evaluate scalability in terms of this overhead, as well as delay and PDR - finding it has linear performance up to their largest scenario of 50 nodes. Fault tolerance is however not treated.

1.31. Energy Efficient Real-Time MAC Procotol

In [40], Kim et al. proposes a scheduler which group a range of timeslots (typically 31) into *Linksets*. The first timeslot in the linkset is used for beacon transmission, while the remaining is used for data. Each node in the network is assigned a linkset, with the idea that the large data-section is sufficient to accommodate any heterogeneous traffic intensity such that deterministic latency can be retained. This will however penalize energy consumption due to idle listening. How the linksets are assigned and distributed are not specified.

In addition, nodes can use 6P negotiations to generate a sequence of cells from leaf to sink for short latency: Starting at the sink, a child node negotiate for a linkset large enough to accommodate all traffic in its sub-tree. Somewhat similar to DeTAS (Section 1.25), the child next assigns the beginning of the linkset to its own child nodes through new 6P negotiations. This process is repeated down to the leafs, resulting in a sequential cells towards the sink.

1.32. Efficient Shared Cell Scheduling Method and Algorithm (DIVVY)

DIVVY [41] focuses on event-based applications where traffic is transmitted sporadic and in an heterogeneous fashion, yet still require a level of reliability and deterministic short latency. The authors argue dedicated cells are sub-optimal for these applications since they either waste energy due to idle listening when there is no traffic, or violates latency requirements if added too scarcely.

First a model for star networks running TSCH *shared cells* and its backoff mechanism is created. Through analysis they find a mathematical expressions for optimal configurations of slotframe length and number of child nodes per shared cell, given requirements for reliability and latency, and optimized for energy. It takes into account differing traffic intensity per child node, yet do require measurement of the current PDR and latency such that the model may account for e.g. the current radio environment.

In DIVVY, each parent node periodically measure these parameters and execute the above calculations. Thus it can create a corresponding schedule with optimized length and level of contention per shared cell. The parent node needs to know the number of child nodes and their offered traffic. It also needs to know the schedule of nodes in interfering range such that collisions can be avoiding when selecting shared cells. The authors suggest this can be collected, and the schedule distributed, through piggy-backing on 802.15.4 beacon frames.

1.33. Low-Latency Distributed Scheduler (LaDiS)

LaDiS [42] aims at large-scale convergecast scenarios. A key trait is that it regards traffic requirements according to payload bytes instead of packets. It is further assumed that nodes can freely aggregate and combine payloads. This allows the number of cells to be optimized such that exactly enough cells to transmit the given bytes are allocated - improving the energy consumption and scalability.

Similar to DeAMON in Section 1.28, scheduling starts at the leaf nodes which transmits scheduling requests to their parents which includes their specific traffic requirement

specified in b ytes. The parent allocates sufficient cells at the beginning of the slotframe and informs the child in its response. This process is then repeated upwards yet with nodes also signaling its rightmost scheduled cells. This is used by a parent to allocate cells sequentially from leaf to root, thus optimizing for short and deterministic latency.

An optional feature is described where the root node, after receiving scheduling requests from all its children, may calculate and broadcast the slotframe length to utilize in the network. This allows for energy- or throughput-optimization. Link loss, topology changes or faults are not treated in [42]

1.34. Slot Scheduling with Adaptive Parameterization (SSAP)

SSAP [43] divides the scheduling problem into two: First it schedules each nodes with at least one slot (ALOS) where the selection of the cell aims solely for reliability, i.e. the cell with the least amount of contention. Secondly, decide an *access period control* which control how often the cell is activated. Here SSAP optimizes for energy, given that a latency can be met.

ALOS scheduling is triggered at the root which assign one cell to each child node from the start of the slotframe. When receiving the assignment, the child node assigns its own children in the same fashion, starting at its own timeslot + 1, yet with a channel offset derived from its own timeslot. This results in a tightly populated slotframe with spatial reuse (and possible contention) depending on the slotframe length, number of channels and nodes.

The authors propose a distributed solution to deciding the access period by employing Lagrangian- and dual functions. To solve the problems, each node must exchange parameters with its parent and children nodes recursively. The authors suggest this is done through piggybacking in packet- and ACK-headers. The result is a schedule in which each node's cell is activated often enough to meet a targeted latency, while optimizing for energy. The authors also argue the distributed solution allows for quick convergence and fault tolerance. In a 43-node network with traffic intensity following a Poisson distribution with mean transmission once every two minute, convergence was found to be between 195 and 700 seconds, depending on the change introduced.

1.35. Low-Latency Distributed Scheduling Function for Industrial Internet of Things (LDSF)

LDSF [44] operates similarly to stratum-based approach by Hosni et al. in Section 1.14. The slotframe is divided into a set of numbered *blocks*, where the even-numbered blocks are used for transmission by nodes which are an even count of hops from the root - and vice versa for odd-numbered. This as opposed to Hosni et al., where one block/stratum was tied to one particular hop distance. This allows LDSF a shorter latency since a packet may be delivered before the end of the slotframe. It also allows for spatial reuse across hops - yet contention may occur and must be handled by a housekeeping mechanism. The size of each block is intended to be small to keep latency short, yet there is a trade-of with scalability since contention would be more likely.

To accommodate retransmissions and improve reliability, additional *ghost cells* are added in every other block after the *primary cell*. The ghost cells are added autonomously through simple calculations after the negotiation of the primary cell, and does thus not incur any additional overhead. The number of ghost cells are set to accommodate the maximum retransmissions possible along a path from source to destination. This as opposed to Hosni et al. and many other which rely on an average ETX. LDSF limits the energy cost of this approach by having nodes sleep in ghost cells after the packet has been successfully received. This requires the traffic intensity to be homogeneous during operation.

2. Autonomous Scheduling

2.1. Orchestra

Orchestra [45] lets nodes build their schedule based on node addresses (or any other unique identifiers/ID) broadcasted in existing mechanism such as TSCH beacons or RPL

packets. Using a hash function, each node ID is translated into a cell, identifying the cell to transmit or receive from that particular node. Thus, the only operation needed to establish a link between two nodes is to know each other's ID and calculate the cell.

In *receiver-based mode*, cells are scheduled according to the receiver ID - consequently there could be contention as multiple nodes could send to the same receiver simultaneously. I.e. when wanting to transmit, a node would use the receiving node ID to identify which cell to utilize - other nodes might do the same, at the same time. However in *sender-based mode*, the cells are scheduled according to the sender ID. This may increase reliability as it allows for a contention-free schedule if the slotframe length is longer than the number of nodes in the network.

In both modes, every node is inherently allocated the same static number of cells, making Orchestra unable to achieve deterministic latency or adopt to heterogeneous traffic scenarios. It is therefore also susceptible to the funneling effect where traffic increases close to the sink. However, the authors points out the slotframe length may be adjusted to trade-off between throughput and energy. Shorter slotframes allow for increased throughput, which may absorb heterogeneous traffic in an opportunisitic fashion. Short latency is however difficult since cells are selected based on the node ID, thus will be "randomly" placed in the slotframe for any traffic path.

2.2. *Autonomous Link-Based Cell Scheduling for TSCH (ALICE)*

With ALICE [47], the author argues the Orchestra node-based approached (assigning cells per node) is inefficient. The key difference in ALICE compared to Orchestra is that cells are assigned to each directional link instead of nodes. With Orchestra, the timeslot and channel hash utilizes either receiver or sender node ID as input. However with ALICE, both IDs are employed, as well as the direction of the link. Consequently, a node will have dedicated cells to receive and transmit to each of his neighbors. To mitigate cell collisions, ALICE makes sure every slotframe is unique: An Absolute SlotFrame Number (ASFN) is introduced, which counts the number of slotframes since network deployment. This is added to the hash input, resulting in a unique schedule for every slotframe - avoiding any persistent collisions.

The authors shows how performance is improved compared to Orchestra. This is especially true as the network scales and traffic increases, since Orchestras allocate only one cell per node regardless of neighborhood, and suffer from increased contention if not setup in its collision-free sender-based mode.

2.3. *Escalator*

Escalator [46] focuses on the convergecast traffic scenario which the authors argue is the main communication pattern for WSNs. They point to the lack of traffic and delay considerations in Orchestra: All nodes get a fixed number of cells allocated. To remedy this, Escalator schedules dedicated cells for the traffic of every node in its sub-tree: When a node receives an RPL DAO message (RPL DAO messages are sent from a node towards the root to allow for downward routing), it immediately schedules a cell to receive unicast traffic from this child, and a cell to transmit this child traffic upwards to its parent. This process repeats as the DAO traverses towards the root, thus creating a dedicated path for this node's upwards traffic - allowing for deterministic latency.

The timeslot offset of these cells are first set by the node id, e.g. traffic from node id 4 will be transmitted at offset 8. Next, a *sliding slotframe* concept is employed where the allocations are left-shifted by the hop count from the sink. Consequently, at hop count 1, the traffic from node 4 will be transmitted at offset 7, at hop count 2 it will be transmitted at 6, and so on. Figure S2 shows this mechanism in an example schedule for a simple network with 4 nodes, v_1 to v_4. Grey cells are broadcasts, while the colored cells are unicasts corresponding to each node in the topology on the left side. Each cell is marked according to the transmitting or receiving node and the traffic original source: E.g. RX_{13} lets node 1 receive traffic originated from node 3. Similarly, TX_{24} allows node 2 to transmit traffic

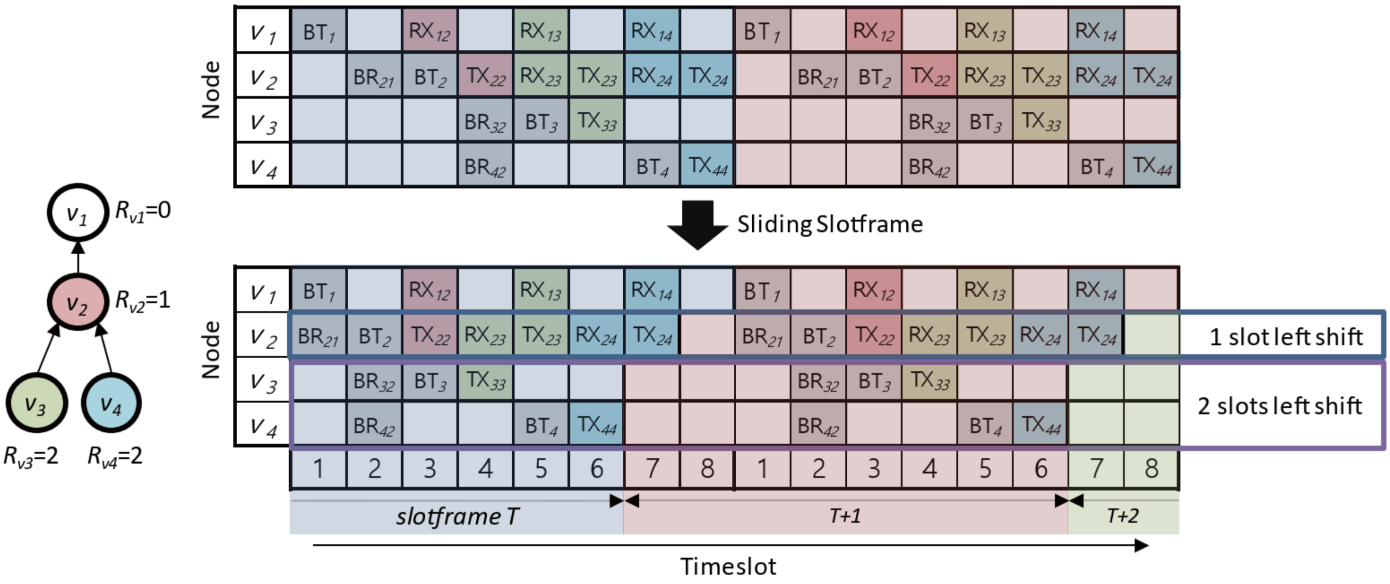

Figure S2. Escalator sliding slotframe [46].

sourced from node 4. The resulting schedule will thus have cells being set consecutively towards the sink (looking at the schedule, it resembles escalators going from bottom of topology to the sink - hence the name Escalator), to minimize the end-to-end delay.

The channel offset for unicast traffic is also decided by the hop count such that one channel is used per two hops: Links at first and second hop uses channel offset 0, third and fourth uses offset 1, and so on. This avoids collisions between operations at same timeslot offset, yet imposes a maximum supported network depth of two times the available channels.

As with Orchestra, the number of cells is fixed, thus there is no support for a heterogeneous traffic scenario, or optimization for energy. Lastly there is no evaluation or objective of scalability identified.

2.4. *Scheduling High-Rate Unpredictable Traffic in IEEE 802.15.4 TSCH Networks*

In [48], the authors address a healthcare scenario in which wearable sensors introduce high-rate traffic into a TSCH network. Since these sensors are highly mobile, it is not possible to predict which portion of the TSCH network has to forward the traffic.

They start out with an autonomous schedule based on node ID similar to Orchestra. However, there is accommodation for one cell for every possible link in the network. This assumes that the number of nodes is less than or equal to the number of channels available. To increase throughput, they implement a cell reuse mechanism where a node is allowed to use its indirect children cells. For example, a node which is a neighbor to the sink can use cells of its indirect children which is allocated for transmission to the sink.

When a node experiences a sudden spike of traffic, or an abrupt reduction of link quality, it needs additional resources. The authors argue this could be done by proactive over-provisioning; however this approach would exhaust the slotframe capacity which needs to be kept short for the high throughput application. Rather they propose adding shared slots which can be utilized to ensure reliability in such cases. The rationale behind this approach assumes change in traffic or link quality will not be experienced by all nodes at the same time, thus the contention for shared slots should be limited. A nodes queue size is used to decide if it should try to employ a shared slot.

2.5. *TSCH Scheduler Supporting QoS Differentiated Service*

Phung et al. [49] focus on a heterogeneous scenario with multiple applications and traffic classes. They let RPL build a routing topology for each type of traffic, and then

schedule for the different RPL instances independently. During a network-wide *scheduling phase*, every node execute a novel trial-and-error strategy by randomly trying to receive or transmit to parents in either instance. This process runs for every timeslot in the slotframe and a reward is associated with every success (a RX or TX). A successful TX must be conducted within a given deadline, this to ensure deterministic latency matching the different traffic classes. The process is then repeated for a given number of slotframes until the nodes converges to a schedule.

Although not discussed, the trial-and-error approach inherently take link quality and thus reliability into account during the scheduling phase. However, [49] does not evaluate the cost of the scheduling phase, neither its energy consumption, overhead introduced or how it would scale. Re-convergence in case of faults would be slow as it seemingly require both a new scheduling phase and for RPL to converge.

2.6. Bringing Opportunistic Routing and Effortless-Scheduling to TSCH (BOOST)

BOOST [50] proposes a simple autonomous scheduler: The network is divided into layers based on hop count from the sink. At even timeslots (2, 4, 6 etc.) all nodes in even layers may transmit, while all nodes in odd layers enable their receiver - and vice versa for odd timeslots. Note that different channels are used by two adjacent pairs of layers to avoid interference. E.g. layer 2, 4, 6 will all utilize different channels, while layer 8 reuse the channel of layer 0 - thus enabling spatial reuse.

All nodes in each layer does contend, which makes the latency probabilistic. The authors do however point out that reliability is improved by receiver diversity: BOOST also include an opportunistic routing, where all nodes in the next layer may accept the data from previous. To improve energy efficiency, receiving nodes may use the historical traffic intensity to adjust their schedule: When no transmission is received in a cell, α is increased by 1, and the node will now skip α number of its scheduled cells. This is repeated until a transmissions is received, upon which α is reset.

2.7. Layered Autonomous TSCH Scheduler for Minimal Band Occupancy with Bounded Latency

Similar to Escalator, the Layered [51] focus on latency in convergecast applications. The slotframe is divided into layers, typically two, with each layer allocated to transmissions at odd and even hops from the root. Every node is allocated one cell for its traffic to be forwarded in each layer, thus guaranteeing a dedicated resource at every hop from source to sink. When a packet is to be forwarded, the source IP is inspected and the corresponding TX cell in the layer is found using a collision-free hash. Reservations are triggered as in Escalator by exploiting RPL DAO packets. The same layer may be used at multiple hops, however, a trade-off in band occupancy and reliability is offered where adding more channels increases the distance in hops between transmissions. Thus, Layered provides spatial reuse while minimizing the band occupied - an objective seldom addressed. This as compared to Escalator which requires one channel per two hops supported. Layered does however induce longer latency depending on the number of nodes and hops needing to be traversed.

3. Hybrid and Static Schedulers

3.1. Adaptive Static Scheduling

The adaptive static scheduling proposed by Fafoutis et al. in [52] focuses on energy efficiency in TSCH network with varying traffic intensities - caused by either the application or channel conditions triggering retransmissions. It assumes a static schedule with a certain over-provisioning to ensure reliability through such traffic events, yet they point out this incurs an energy penalty in low traffic periods due to idle listening. The authors therefore propose nodes to continuously monitor their queue size and slot utilization to deduce S_a which denotes the actual number of slots to utilize, out of all the statically allocated slots. S_a is piggybacked in packet-headers between neighbors and they are thus able to

synchronize how many slots should currently be used. Since this exchange is necessary, we categorize this portion as collaborative, and thus the scheduler as a hybrid.

3.2. Parameterized Adaptive and Autonomous Scheduling (PAAS)

PAAS [53] intends to improve the autonomous scheduler Orchestra (see Section 2.1) by making it adaptable to traffic intensity, thus improving support for heterogeneous traffic. Since PAAS requires exchange of dedicated information through piggybacking on RPL DIO messages, we classify it as a collaborative and autonomous hybrid.

Each link in PAAS is, as with Orchestra, limited to be implemented by maximum 1 dedicated cell. PAAS however optimizes for energy by having multiple low-traffic links contend for a shared cell in varying degree: Each node monitors its number of children, and the volume of traffic received. This is utilized to calculate the number of child nodes which could share one cell and still satisfy a given reliability requirement (i.e. a collision probability) - this number is denoted as n. The parent node then piggybacks this information on a RPL DIO message, such that children can adjust their schedule to achieve the slot sharing dictated by n. As traffic intensity changes, the process is repeated, always aiming to find a given balance between the reliability requirement and energy efficiency. PAAS also define a separate *Recovery phase* to handle node faults, significant link quality changes, and mobility scenarios, in which the schedule is reset and re-built with new traffic estimations.

3.3. Autonomous and Traffic-Aware Scheduling for TSCH Networks (e-TSCH-Orch)

As with PAAS, e-TSCH-Orch [54] aims to improve on the autonomous scheduler Orchestra. Through simulations they illustrate that Orchestra suffers under the funneling effect, leading to unbounded delays when traffic intensifies such as in heterogeneous scenarios.

To address this, e-TSCH-Orch allows a node to schedule additional cells when needed: If a node is unable to empty its queue through the regular schedule, it signals the neighbor to add additional cells. The number of new cells are equal to the node queue size, or as many as the slotframe length allows. This number is signaled at the end of a packet, which upon reception triggers the neighbor to schedule additional rx cells. These new cells are simply added consecutively after the regularly scheduled cell. Since this mechanism requires additional dedicated information to be sent between nodes, we categorize it as a hybrid scheduler. Lastly, e-TSCH-Orch introduces the concept of critical and periodic traffic classes, in which critical traffic is always put in front of the queue.

3.4. Traffic-Aware Elastic Slotframe Adjustment in TSCH Networks (TESLA)

Similar to both PAAS and e-TSCH-Orch, TESLA [55] aims to add support for heterogeneous traffic in Orchestra, as well as improving its energy efficiency. It builds on top of receiver-based Orchestra, where each node have a RX-cell, with other nodes contending when transmitting. TESLA adds a mechanism for nodes to adjust their slotframe length: All nodes signal their traffic load to neighbors by piggy-backing it in every unicast packet. Receivers use this to lengthen their slotframe if load is light - which improves energy efficiency. Similarly, the slotframe is shortened if traffic is heavy. For neighbors to be informed of the new slotframe length, nodes piggy-back it in acknowledgments, RPL packets and beacons.

3.5. On-Demand TSCH Scheduling with Traffic-Awareness (OST)

Continuing the trend, OST [56] expands on Orchestra to support heterogeneous traffic. The authors point out how static allocations risk wasting energy due to over-provisioning or suffer poor reliability and long latency due to under-provisioning. To address these issues they add three mechanisms: Firstly, a new *Periodic-provisioning slotframe (PPS)* is added per neighbor. This contains one cell, but its length is dynamically adjusted to accommodate the average traffic load, similar to TESLA. The synchronization between nodes happens by

piggybacking information on unicast packets and ACKs. Secondly, *on-demand provisioning (ODP)* is introduced to handle sudden bursts. The principle is similar yet more advanced than what is found in e-TSCH-Orch: When a node has two or more packets queued towards a neighbor, it piggybacks a bitmap of upcoming available timeslots towards the neighbor. In the ACK, the neighbor responds with the first common timeslot available. A one-time cell is then added at both neighbors allowing for additional data to be sent. This process is repeated until the queue is empty. Lastly, to avoid continuous collisions, channel offsets are calculated using ASFN (see ALICE in Section 2.2) and ASN for PPS and ODP respectively.

3.6. Multihop and Blacklist-Based Optimized Time Synchronized Channel Hopping (MABO-TSCH)

MABO-TSCH [57] focuses on the list of channels utilized by nodes when following the TSCH frequency-hopping scheme: Its main idea is a distributed way for nodes to 1) Estimate channel quality, and 2) maintain and synchronize a black-list of channels with degraded performance. By avoiding these channels when hopping, links can achieve significantly improved packet delivery ratio.

However, MABO-TSCH also includes a centralized scheduling mechanism, yet with only the channel-offset selection described in [57]. We therefore include a short description: It defines the task as a graph coloring problem and employs a *greedy degree-ordering* algorithm to solve it. First nodes are listed in an array and sorted in a non-increasing order according to their degree. The greedy algorithm is then applied to each node, assigning a color/channel-offset which does not have any conflicts or interference. This step is repeated until it is not possible to assign any colors. This leaves each node with a set of non-interfering channel offsets for each time slot, available for selection by later mechanisms in MABO-TSCH.

3.7. Adaptive MUlti-hop Scheduling (AMUS)

AMUS [58] assumes a Path Computation Entity (PCE) with knowledge of the physical- and routing topology, as well as the offered traffic per node. It builds a Multi-hop Scheduling Sequence matrix (MSS) which maps out all links pertaining to each signaled path in the topology, together with their associated traffic rate. The links, in each matrix column, are ordered such that the first hop from the source is placed in the earliest column. Next, cells are assigned to each link in the matrix, with an amount matching the required rate. This assures sufficient resources as well as a sequential allocation - aiming for deterministic and short latency. The actual selection of cells follows similar *scheduling rules* as TASA: Cells which results in conflicts or interference are avoided, while spatial reuse is allowed wherever possible.

AMUS also introduces the concept of a *Tentative Schedule* where additional *backup cells* are allocated for all links - to be used in cases where a retransmission is necessary. This is to ensure reliability and latency in cases of degraded wireless link quality, without involving the PCE. The backup cells are however only for active links, i.e. will not alleviate a situation of e.g. node fault or topology change. The authors do however realize this would incur a significant energy penalty, as nodes would be idle listening in unused backup cells. They therefore propose nodes to collaborate by informing parents when all packets for the current slotframe has been sent. This is done with a *End-Of-Queue* (EoQ) notification either piggybacked or dedicated, allowing the parent to avoid idle listening for the remaining slots in the slotframe - thus conserving energy.

3.8. Hybrid Schedule Management in 6TiSCH

Karaagac et al. TimecriticalcommunicationKaraagac2018, HybridScheduleManagementKaraagac2018 notes that centralized approaches can theoretically obtain better performance, yet suffer in terms of scalability and flexibility, where decentralized solutions may excel. They hence explore different ways centralized and decentralized schedulers can be combined:

1. *Determinism and flexibility*: Nodes allocate resources for non-critical traffic in a decentralized manner, while a centralized scheduler is employed for critical traffic.
2. *Local support for centralized scheduling*: A centralized scheduler handle all traffic, except during network bootstrapping and unstable network conditions where a limited set of cells are allocated by a decentralized solution to maintain connectivity.
3. *Infrastructure-based*: Assuming a network where nodes are divided into static infrastructure nodes, and mobile nodes (similar to the healthcare scenario tackled by Elst et al. in Section 2.4) - a centralized scheduler is responsible for scheduling the infrastructure, while communication between mobile nodes and infrastructure nodes are handled in a decentralized manner.
4. *Centrally assisted distributed scheduling*: All allocations are handled decentralized, yet with a centralized scheduler utilizing its global view to add improvements such as load balancing.

Although Karaagac et al. focus on exploring the concept of hybrid scheduling and feasibility of its implementation, they conduct a brief experimental evaluation of the first approach presented above. They employ a heterogeneous convergecast scenario with periodic non-critical traffic as well as event-driven time-sensitive critical packets. The centralized scheduler itself is out of the authors scope, so they assume a pre-calculated optimized schedule, which allocates a path of deterministic and short latency for the critical traffic. The remaining periodic traffic is handled in a decentralized fashion using Scheduling Function Zero (SF0, see Section 1.2).

3.9. Centralized Link Scheduling (CLS)

CLS [59] aims to reduce the overhead, i.e. control packets, typically associated with centralized schemes. As opposed to most other schedulers who collects all information and next builds a schedule, CLS continuously edit the schedule as request are received from nodes. This approach is similar to collaborative hop-by-hop scheduling, as seen with CFDS in Section 1.1. However, with CLS the cell time- and channel-offset is decided at the central PCE.

When a node needs resources, it creates a CLS *allocation request* in which it puts a link request entry containing the node address, its rank, the parent address, and the amount of cells required. The allocation request is transmitted towards the sink, and at each relaying node, a corresponding entry is added to the allocation request. This is necessary to achieve deterministic latency, as each node in the path needs additional resources to handle the new traffic. When received at the sink (where the scheduler resides), the request contains a list of entries detailing the traffic requirements, populated by all nodes from the source to sink.

At reception, the scheduler goes through the list starting with the source entry and assign cells accordingly: The timeslot offset is set as the "last assigned time slot of the link parent" plus 1. The channel offset is set to node rank minus 1. If there is already an allocation in this cell, the next timeslot is tried. This results in a collision-free schedule where a packet is delivered in one slotframe for short latency. After allocating for all the entries, an assign-message is transmitted to the requesting nodes for them to update their schedule.

For de-allocation, CLS utilizes a collaborative strategy where a node locally immediately deallocates the relevant cells, and signals this to its parent in a CLS *deallocation request* message. This is repeated until it reaches the sink, upon which the scheduler removes the cells from its view of the schedule - thus minimizing overhead since no re-calculation or distribution of new schedule is required. Through simulations the authors show how this allows the network to scale and handle disruptive events such as parent changes with limited overhead. The request-based approach of CLS allows it to support heterogeneous applications, and adapt allocation to the node requirements, consequently saving energy by de-allocating when possible.

3.10. Quick Setup Scheduling (QSS)

CLS is enhanced in [60], which introduces the scheduler QSS. The key difference is the addition of a *Optimization Processing* step in the scheduler. In CLS, the allocation algorithm is such that it will result in a slotframe close to the minimum length possible. However, as cells are added and removed based on node requests, it is likely that the size of the slotframe will move longer and longer away from the optimal minimum. QSS therefore monitors the frequency of incoming requests, if it evaluates the network as stable, it will execute a optimization step. In it, the schedule is rebuilt to achieve the minimum slotframe length, and the result is signaled to all nodes. This is done to improve the end-to-end delay, yet it introduces some additional overhead.

3.11. Frame-Type Aware Static Scheduling for Large-Scale Smart Metering Networks

In [61], the authors focus on smart metering network where a large number of devices report data in a periodic and/or sporadic fashion. To address scalability, they suggest assigning dedicated cells for broadcast and unicast frames. This is key especially for broadcasted network control traffic, which typically suffers from collisions as the network scales. A static schedule is proposed, where the first timeslot is allocated for broadcast traffic and the remaining for unicast - all of which are shared among all nodes. The slotframe length is set before deployment to achieve the desired broadcast-to-unicast ratio.

The resulting schedule is similar to a slotted ALOHA approach with every cell contended. The authors meet their reliability and latency objectives through significant over-provisioning, since nodes are active in every timeslot. This also allows the scheduler to support any topology or heterogeneous traffic intensity among the nodes. Obviously, this is traded for increased energy consumption.

3.12. Scheduling of Dedicated and Shared Links for Fast and Reliable Delivery

Khorov et al. [62] focuses on the impact of shared cells in static scheduling, similar to Elst et al. in Section 2.4. Khorov et al. investigates a static scheduler in which every node has the same amount of resources, plus a fixed portion of shared cells. Through simulations in Cooja they show how an optimal amount may be decided a priori depending on how the shared cells are utilized, e.g. if they are reserved for retransmissions only.

4. Centralized Scheduling

4.1. Traffic Aware Scheduling Algorithm (TASA)

TASA was proposed in [63] and further evaluated in [64] - it is one of the earlier schedulers targeting 802.15.4e TSCH, and is often used as a benchmark for newer proposals. It assumes the PCE knows the routing- and physical topology (interference domains), and the offered traffic per node. The application is convergecast only, with an assumption of every node having on average over time the same traffic rate.

The authors define a network model which is utilized in several later schedulers. This include sets of conflicts: A node cannot receive from multiple nodes, nor can it transmit and receive at the same time - this is called duplex-conflicts (later schedulers denote this simply as *conflicts*). The *duplex-conflict-free links* (DCFL) set is constructed per timeslot and identifies the links which can be scheduled in the same timeslot without such conflicts. Similarly, but per cell, the *interference-conflict-free links* (ICFL) set contains links which can be scheduled in the same cell without interfering, i.e. spatial re-use.

TASA aims to make the slotframe as compact as possible to keep latency low, while avoiding any conflicts or interference between nodes. To achieve this, it iteratively goes through each timeslot and employs techniques from graph theory: In the *matching* step, a DCFL is built by starting at the top of the routing tree and prioritizing nodes with the highest traffic demand. Secondly, the *coloring* step builds the ICFLs for each cell in the timeslot, also here prioritizing traffic demand - this yields the channel-offsets to be utilized for each of the links in the DCFL. These steps are repeated for every timeslot until all traffic demands are met.

By default, the amount of cells allocated matches the signaled load, thus aiming for deterministic latency. However, the authors point out that over-provisioning could be applied for increased reliability. Loss of packets can be detected if TASA is located at the root: It knows the expected traffic and can thus detect topology changes, node- or link failures. However, this would require a re-collection of information from the network, re-computation of a new schedule, and finally re-distributing it.

4.2. TASA with Hop-by-Hop Retransmission Capability (TASA-RTX)

Gaillard et al. proposes an enhancement to TASA in [65]. They note TASA does not consider link packet losses and thus introduce an overprovisioning mechanism to increase its reliability.

They assume a network with multiple convergecast applications, each with its own Packet Delivery Ratio (PDR) requirement that must be met by the scheduler. Knowing the PDR of each link it runs a *inverse greedy algorithm*: Each link along an applications path is first assigned its maximum allowable cells (e.g. 9 (1 transmission and 8 retransmission attempts)). The schedule is then evaluated against the application PDR requirement - if not met, it is not possible to make a valid schedule. Otherwise, one cell is removed from the most loaded link (i.e. with the most previous + current allocated cells), its load is updated, and the schedule is re-evaluated. If still valid, these steps are repeated on the current most loaded link. When the schedule does not meet the requirement, the last removed cell is re-added, the link is marked as treated, and the process repeats until all links are treated. By targeting the most loaded links first, the algorithm aims to load-balance the allocation of overprovisioned cells.

4.3. Queue-Based Scheduling Algorithm for PCE-Enabled Industrial Internet of Things Networks

Based on the same network- and conflict models as TASA, Farias et al. [66] propose a scheduler which has complete knowledge of the routing topology. It relies on each node submitting traffic requests which thus allows for heterogeneous traffic and deterministic latency as sufficient resources are allocated. The scheduler fetches the requests from a request queue, hence the term *queue-based scheduling*, and allocates them one-by-one. As opposed to TASA it does not aim to minimize the slotframe size and allocate cells sequentially towards the sink - it rather distributes the cells for each link as uniformly as possible across the slotframe. This to decrease the intra-slotframe delay.

4.4. Multichannel Optimized DElay Time Slot Assignment (MODESA)

MODESA [67] is an early scheduler with a sole aim to minimize the slotframe length, i.e. use the least amount of timeslots, to reach objectives of short latency and high throughput. The authors identify these objectives from convergecast data-gathering applications in the aerospace domain. They assume no loss at the physical layer and no failure of nodes, and lastly also considers multi-radio sinks.

Nodes are sorted after *priority* which is defined as the nodes number of unscheduled packets multiplied with the number of packets its parent has to receive this slotframe. Thus, "busy" nodes on a "busy" sub-tree has high priority. As with TASA, the algorithm works iteratively and starts at the first timeslot, however it assigns first to the node with the highest priority. It continues through the sorted list of nodes and tries to assign the same cell to multiple nodes if conflict and interference can be avoided - thus employing spatial reuse which is key to reduce the slotframe size. If allocation is not possible within the constraints, another channel-offset is tested. After iterating the sorted node list, the priorities and hence list of nodes is updated, and the algorithm is re-run on the next timeslot. This is repeated until all nodes have zero unscheduled packets, thus deterministic latency is achieved.

4.5. Robust QoS Scheduling

Wu et al. [68] solely target the fault tolerance problem. They propose a scheduler which handles link or node failures by preparing alternate paths: Additional *margin cells* are added from a node towards alternative parents upwards to the sink. These cells are added in separate portions of the slotframe, with individual portions being allocated per rank in the topology.

When a link fails, a node will utilize the alternate path which is available through the margin cells.

4.6. Assignment of Segmented Slots in IWNS

Yang et al. [69] proposes three separate mechanisms to increase reliability and fault tolerance, which are evaluated in a monitoring scenario with homogeneous convergecast traffic.

The *Fast Slot Competition (FSC)* assumes a network where some shared slots are present to be utilized for retransmissions in case of link packet loss. In the shared slots, FSC adds several, e.g. four, Clear Channel Assessments (CCA) opportunities (*CCA slot*) at the beginning of the slot. CCA is a common technique where the medium is first sensed for traffic, before being utilized for transmission. When a node wants to transmit in the shared slot, it randomly selects one of the CCA slots. If the medium is found to be free, it transmits a preamble for the remaining CCA slots to block other nodes, before the actual data transmission starts. This is done to improve the retransmissions success rate.

With *Segmented Slot Assignment (SSA)*, a short and deterministic latency is aimed at by providing nodes with resources such that all data is received at the sink within the same slotframe. This is achieved by assigning cells in segments based on the hop count, with the highest hop count in the first segment in the slotframe. This is similar to the *stratum* approach employed by Hosni et al. in Section 1.14. To tackle retransmissions, it also adds a group of shared slots at the end of each segment - this way, a retransmission can happen before the segment of the next hop towards the sink.

Lastly, a concept of *free node* is introduced, which is any node one hop away from the sink without children. These are utilized to reduce the impact on the schedule when a link or node failure occurs: The mechanism rests on the principle that the cell of a free node towards the sink could be scheduled anywhere in the slotframe, as it would in any case meet the latency requirement. Thus this cell can be simply swapped with other nodes in need, incurring limited overhead.

4.7. Dawn

Dawn [70] lets nodes individually calculate the required bandwidth, and signals this to a PCE. The focus is put on how these requirements can be modeled and collected by each node: Dawn utilizes a *Loosely-coupled Component Infrastructure* (LooCI) as a backbone, which allows nodes to be modeled as a set of components (e.g. sensors, actuators, aggregators). Each component has certain requirements, e.g. a sensor on a node which needs some bandwidth to transmit its data, or capabilities, e.g. a node capable of buffering and aggregating data.

The *compositional analysis algorithm* and *bandwidth allocation algorithm* running on each node evaluate both its local components and remote components (i.e. other nodes), to identify the bandwidth requirements. The result is signaled to the network manager which allocates the required slots to accommodate the signaled need.

Since each node takes the traffic from remote nodes into account, sufficient resources, and thus deterministic latency, along the entire path is aimed for. Heterogeneous applications are handled by design, as a component is allowed to be removed, added or changed at any time. This triggers a re-run of the algorithms, and a new request to the network manager to update the allocations.

The actual operation of the centralized scheduler is not described, making any further evaluation difficult.

4.8. Scheduling for Data Collection in Multi-Hop IEEE 802.15.4e TSCH Networks

Chen et al. [71] considers a data collection application with homogeneous convergecast traffic p atterns. They assume a random deployment with no packet loss at the physical layer, with every node performing aggregation of data when relaying.

The main objective is to minimize the length of the slotframe. Allocations are constrained to avoid interfering or conflicting cells, as the scheduler is assumed to know the physical- and routing topology. In the homogeneous traffic scenario, each node is assumed to generate one packet per slotframe, with deterministic latency achieved by meeting this demand.

Three separate implementations are proposed to meet these objectives, with each expanding and improving on the other. The level-based selection scheduling (LSS) starts at the left-most leaf in the tree and assigns the first available timeslot-offset and any available channel-offset. Both timeslot and channel must be chosen such as to avoid conflicts. If there is no channel available, the next timeslot is utilized. This process is then repeated until all nodes have been visited and assigned one cell. Next, the entire process is repeated a number of times equal to the tree-depth minus one. These repetitions are denoted as *rounds*, and assures all data can be delivered to the sink during one slotframe.

As pointed out in [71], LSS can be optimized since it would allocate multiple cells per slotframe to e.g. a leaf which only needs to transmit one packet. Leaf Pruning Scheduling (LPS) is therefore introduced, and is which basically identical to LSS except that after each round, the tree is pruned for any leaf - thus avoiding the unnecessary allocations.

Lastly, the authors introduce pipeline scheduling (PS) which is employed on top of LSS and LPS. However, the details of this mechanism are not described except of some indication it could concern spatial reuse of cells.

4.9. An Efficient Centralized Scheduling Algorithm

Ojo et al. explores a graph theoretical approach to centralized scheduling in [72]. The authors assume a convergecast scenario and apply a connectivity and interference model based on physical distance between nodes. Based on this they provide two formulations of the scheduling problem; one as a throughput maximization problem, and one as a delay minimization problem.

Next, they propose a solution to the throughput maximization problem. The scheduler thus solely optimizes for network throughput, while avoiding conflict and interference. It does however assume each node signal, at the start of each slotframe, their queue size and number of packets transmitted. Thus a deterministic latency and support for heterogeneous traffic should be achievable, with significant ov erhead. For the solution to be solved in polynomial time, they first transform the problem into an equivalent maximum weighted bipartite matching (MWBM) problem. They are then able to approximate a solution by employing a *Hungarian algorithm* [72], which has reduced computational complexity. Through simulation they show this approximation to be close to the optimal throughput.

4.10. An Energy Efficient Centralized Scheduling Scheme

In [73], Ojo et al. focuses on energy optimization for TSCH networks. The models, constraints, and assumptions are adopted from the work in [72], described above.

A TSCH energy consumption model is introduced and utilized to formulate the scheduling problem as an energy efficiency maximization problem. The authors note it is a non-linear programming problem which is difficult to solve, and they therefore propose two low-complexity solutions. Both assume the scheduler is able to calculate the energy efficiency (bits per joule) of each node for each cell according to their energy consumption model.

Energy Efficiency Scheduler (EES) is a simple heuristic scheduler, which uses a greedy approach: If multiple nodes are destined for allocation in the same cell, the node with the highest energy efficiency for this cell is selected.

Vogels Approximation Based Heuristic Scheduling Algorithm (VAM-HSA) is a more refined solution which also considers the next assignment before making a decision. This is to avoid situations where a good efficiency in one assignment results in poor results in the next. This VAM approach have been shown to give results close to the optimal.

4.11. Approximate Dynamic Policy (ADP)

ADP [74] focus on convergecast applications with a tree routing topology where each node have selected a parent out of several alternatives - e.g. as done in RPL. However, ADP proposes to have a subset of these alternative parents to listen for traffic in the same cell. If one of the parent nodes receive the packet, it replies with an ACK (overhearing and a priority-scheme is utilized to avoid packet duplication and ACK collisions), and forward the packet upwards. This way, a packet is lost only if none of the parents are able to receive - thus greatly increasing the reliability.

This does however increase energy spent listening, yet ADP employs a technique to conserve energy: The set of parents is selected based on the channel condition, such that transmission energy is optimized. I.e. parents which allows the transmitting node to reduce its output power. This assumes nodes measure the channel conditions and communicate its quality (following a Markovian channel model introduced in [74]) to the Path Computation Element (PCE) before every slotframe. The schedule is also expected to avoid conflicts and interference, as well as allocate enough resources for deterministic latency in heterogeneous scenarios - thus it is assumed the physical topology and nodes queue size is known to the PCE. Scalability and overhead of these requirements and assumptions are mentioned as future work.

Finding an optimal schedule meeting the constraints above is described as NP-hard. They therefore employ an *approximate dynamic programming scheme* which is shown to be close to the optimal solution. The details of this scheme can be found in [74].

4.12. Scheduling Transmission with Latency Constraints in an IEEE 802.15.4e TSCH Network

Khoufi et al. [75] first determine theoretical bounds on maximum delivery time and minimal number of slots needed to meet certain requirements in a industrial data gathering application. They next propose a centralized *debt-based* scheduler which meet these, and aims to be "(...) collision-free, traffic-aware and minimizes the data gathering delays.".

Knowing the routing topology and the offered traffic per node, it operates iteratively starting at the node with the highest *debt* (the number of packets remaining to be scheduled, multiplied with rank, i.e. hops from sink). If the node and its parent are not already scheduled, it inserts a transmission in the current cell. Otherwise it moves to the next node in terms of debt. This process is repeated, increasing the channel-offset, and next the time-offset, until all packets are scheduled - aiming for a minimized slotframe length. This way, sequential allocation towards the sink is possible, and the resulting schedule should allow for all packets to be delivered to the sink within one slotframe - achieving deterministic and short latency.

The authors discuss spatial reuse of cells, which allows for a cell to be used by two links simultaneously. However, they note this requires a significantly increased level of signaling messages for the scheduler to be able to calculate, thus opts it out of their evaluated solution. Lastly they also evaluate the concept of multi-interfaced sinks and its impact on latency.

4.13. PRuning-Based COloring Scheduling (PRCOS)

PRCOS [76] aims to maximize the lifetime of the most power-consuming node, such that the network lifetime is prolonged, i.e. it focuses on the *hotspot problem* where certain nodes, typically close to the sink, consume a lot of energy.

To achieve this, a PCE is assumed with knowledge of the physical topology as well as the node remaining energy. In addition to scheduling, PRCOS also generate the routing topology. This is done before scheduling, and contains the main contribution towards

increased network lifetime: It utilizes the *ECORS* routing algorithm [77] which optimizes for node remaining energy.

The scheduling algorithm utilizes pruning and coloring techniques, borrowed from graph theory. The timeslots are allocated via the pruning process, with allocation starting at the leaf such that cells are ordered in sequence towards the sink - thus shortening latency. To avoid collision among neighbors, coloring is employing to select the channel offset. Although not explicitly stated it does seem like PRCOS aim for deterministic latency by ensuring resources are allocated for each flow from source all the way to destination.

[76] does not treat other aspects such as fault handling, or traffic requirements from nodes beyond stating their assumption for a convergecast data collection scenario.

4.14. Minimal Idle-Listen Centralized Scheduling (MILS)

The MILS centralized scheduler [78] is proposed with the aim to minimize idle listening. The scheduler is formulated as a Constraint Satisfaction Problem (CSP), and assumes the scheduler has knowledge of the physical and routing topology. It focuses on transmitting data towards the sink with a shortest possible end-to-end delay, thus it daisy-chains the cells as long as no conflicts arises. In the evaluated scenario, each node has a small fixed number of cells allocated (number of children + 1), and one burst of traffic is sent from each node towards the sink. Through simulation it is shown that the queue delay is minimized and the amount of idle listening, and thus energy consumption, is kept low. The scheduler operation is scarcely described; thus a more thorough evaluation is not possible.

4.15. Adaptive Schedule for Industry 4.0

Minet et al. [79] describes a simple scheduler which they evaluate on topologies with max two hops. To keep the complexity limited, they do not employ spatial reuse. This reduces the amount of information required by the scheduler: Nodes report only their neighbors and its offered traffic. After collecting this information, a debt-based approach is applied to build the schedule. This is similar to Khoufi et al. in [75] where nodes with the largest debt (depth times packets remaining) are allocated first. This allows for sequential scheduling, resulting in short and deterministic latency.

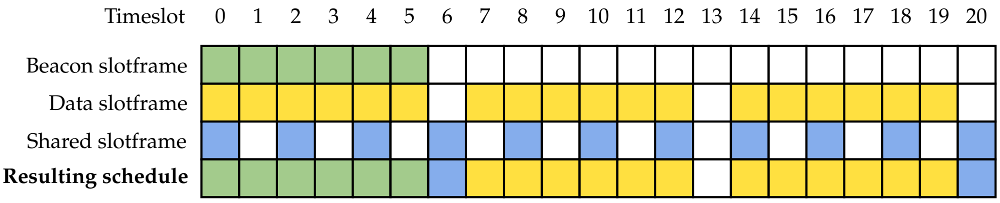

Figure S3. Example schedule built by multiple slotframes, from Minet et al. [79].

The schedule consists of three slotframes which execute simultaneously. They are prioritized in the following order: Beacon-slotframe for 802.15.4 beacons which also piggyback the schedule itself, a slotframe for application-data, and a final for shared slots which can be used to e.g. notify the coordinator of any new topology information. An example of these slotframes and the resulting schedule can be seen in Figure S3.

They assume the sink has multiple radio interfaces. This is exploited by the scheduler which allocates multiple cells towards the sink in the same timeslot to increase the throughput. Further they focus on the adaptability of the schedule: The periodically allocated shared cells allows nodes to promptly notify the coordinator of any faults, topology- or application-changes, etc. An adjusted schedule can be distributed in the beacon slotframe and will go into effect after the next beacon slotframe.

4.16. Content Centric Cross-Layer Scheduling (CONCISE)

In [80] a larger framework is proposed including not only scheduling but also e.g. routing and data aggregation - all being content-aware. CONCISE introduces a centralized *controller* which has information on the physical topology and the rate per content-type per

node. Using this it builds independent routing topologies and schedules for each type of content.

CONCISE first builds a tree-based routing topology, taking aggregation into account: It assumes all relaying nodes can perform aggregation of messages of same content type. This reduces the amount of traffic sent, number of cells needed, and consequently the node energy usage.

Scheduling is done using a strategy similar to TASA: It iteratively assigns cells matching the signaled requirements to achieve deterministic latency, while avoiding conflicts and interference. However, with CONCISE, the resulting schedule is evaluated for funneling effect and traffic hot-spots; if found, a *path optimizer* routine is executed to find a more optimal routing topology, before running the scheduler again. This cross-layer strategy is repeated until an optimized schedule is found, or the number of iterations reach a set threshold.

CONCISE also introduces a concept of *premium timeslots*, which can be used for emergency traffic requiring short delay. The authors do not details their allocation, but gives an example of inserting such timeslots at fixed intervals based on the available resources in the network. Packets with critical data should be marked by a special byte in its header, and any node are then allowed to utilize the premium timeslot for transmission.

4.17. Scheduling via Max-Product Message-Passing

In [81], the main motivation is to avoid interfering transmissions and employ spatial reuse to increase reliability and throughput.

It is assumed the routing topology and interference is described by a conflict graph showing which links are destructive to each other. Further the scheduler must know nodes' traffic load such that sufficient cells can allocated for deterministic latency. Each node is assigned a weight based on this load. The scheduling task can then be stated as, for each cell, to select a set of transmitting nodes which 1) Do not interfere and 2) With priority given to the largest weight. This is equivalent to finding a Maximum Weighted Independent Set (MWIS), which is known to be NP-hard. The authors therefore suggest an approximate solution using probabilistic graphical models and a *message-passing max-product belief propagation* method with limited complexity. The details of the method can be found in [81].

4.18. Scheduling of Periodic Real-Time Flow (SPRF)

SPRF [82] aims to accommodate as many as possible streams of traffic, between any two nodes, with a latency deadline. It assumes a centralized scheduler with knowledge of all possible node links and their interference domains. In addition it must know all the desired flows of traffic.

The schedule is built in an iterative fashion with the following being executed for every timeslot: First, links are arranged after priority based on the flows they are carrying. Flows which have many hops and shorter time until their deadline are prioritized. Second, maximum matching on the graph of all the links (starting with the highest priority) are found using a *blossom algorithm*. The result is a set of non-conflicting (as defined in Section 4.1) links which can be scheduled in this timeslot. However, the links are not guaranteed to be interference-free. This is handled in the last stage where an interference-graph is constructed and links are *colored* with channel offsets following a heuristic algorithm. All variables are finally updated and the procedure repeated for the next timeslot.

This yields a schedule where all flows are received within one slotframe. It also maximizes throughput since it minimizes slotframe size through spatial reuse. Reliability is increased by a local repairing mechanism, in which nodes who do not receive an acknowledgment scans its schedule and retransmit in the nearest available timeslot.

4.19. Scheduling for Dedicated and Shared Links

How to best schedule for retransmissions is the focus of the centralized scheduler by Khorov et al. [62]. A static approach to this problem is found in Section 3.12. They assume a network with convergecast traffic, where the centralized scheduler is able to broadcast a new schedule to all nodes in the first timeslot of each slotframe. The scheduler divides the slotframe into three parts: The round-robin segment gives every node a guaranteed amount of dedicated cells for the traffic at hand. Next, it is assumed the scheduler is aware if failed transmissions, and hence it allocates dedicated cells for retransmissions in the retry segment to increase reliability. Lastly, a shared segment allows for opportunistic transmissions without having to wait for the allocation of a dedicated cell in the next slotframe.

4.20. Optimized Scheduling for Time-Critical Industrial IoT

Brun-Laguna et al. [83] focuses on what they argue is the key metrics for time-critical industrial applications: Latency, reliability and energy. They analyze four different approaches which may be applied by a centralized scheduler which knows all network flows, the network topology, and all link qualities. They find the *load-based* scheduler to be the most optimal in their convergecast traffic scenario. In this approach, the node with the highest load is scheduled first, where load defined as the total number of cells needed to transmit all flows through it. This as opposed to e.g. *cascading* approach where individual flows are scheduled in sequence for minimal latency and buffer requirements. The link quality is also taken into account and additional cells are added to reach the reliability target. Trace-based simulations show how the number of timeslots is kept minimal, thus conserving energy. The authors also explore prioritization of packets, and show how it may greatly reduce the maximum latency if "old" packets are prioritized.

4.21. MASTER: Long-Term Stable Routing and Scheduling

MASTER [84] addresses one of the key issues in centralized scheduling, namely how to accommodate a varying radio environment while keeping overhead due to scheduling updates limited. The key mechanism is a focus on flow-based retransmission, where over-provisioned resources are assigned to flows of traffic instead of particular links. These resources can be utilized by any nodes forwarding traffic in the flow. This allows the schedule to adapt to fluctuations in ETX across multiple links without re-scheduling. The schedule and over-provisioning is decided based on the physical topology of the network, including ETX measurements for all links. Given a set of flows, the schedule is built to accommodate the specified flow latency deadlines, and include over-provisioned shared resources according to the measured ETX.

The reduced overhead will most likely be a significant contribution to the scalability of MASTER, however the evaluation is limited to a 20-node testbed. Other scalability challenges might include the amount of data needed during the bootstrapping phase, in which all nodes measure link properties for collection at the centralized scheduler.

4.22. Fair Scheduler Based on Shell Game

Portaluri et al. [85] is a minimal scheduler which focuses solely on the number of cells which are allocated, and how these can be made fairly between requests from nodes. The scheduler follows a simple algorithm where a penalty counter is maintained for each node per slotframe: When receiving multiple requests for resources, the node with the lowest penalty is served first. The penalty is increased for every cell allocated, such that any node which were unable to be served in a slotframe would have an advantage in the next. The authors show through simulations how this improves fairness compared to other strategies such as round-robin or random allocation.

1. Morell, A.; Vilajosana, X.; Vicario, J.L.; Watteyne, T. Label switching over IEEE802.15.4e networks. *Transactions on Emerging Telecommunications Technologies* **2013**, *24*, 458–475. doi:10.1002/ett.2650.
2. Viswanathan, A.; Rosen, E.C.; Callon, R. Multiprotocol Label Switching Architecture. RFC 3031, 2001. doi:10.17487/RFC3031.
3. Awduche, D.O.; Berger, L.; Gan, D.H.; Li, D.T.; Srinivasan, D.V.; Swallow, G. RSVP-TE: Extensions to RSVP for LSP Tunnels. RFC 3209, 2013. doi:10.17487/rfc3209.
4. Palattella, M.R.; Watteyne, T.; Wang, Q.; Muraoka, K.; Accettura, N.; Dujovne, D.; Grieco, L.A.; Engel, T. On-the-Fly Bandwidth Reservation for 6TiSCH Wireless Industrial Networks. *Sensors Journal, IEEE* **2016**, *16*, 550–560. doi:10.1109/JSEN.2015.2480886.
5. Dujovne, D.; Grieco, L.A.; Palattella, M.R.; Accettura, N. 6TiSCH 6top Scheduling Function Zero (SF0). Internet-Draft draft-ietf-6tisch-6top-sf0-03, Internet Engineering Task Force. Work in Progress.
6. Chang, T.T.C.; Watteyne, T.; Qin, W.; Vilajosana, X. LLSF: Low Latency Scheduling Function for 6TiSCH Networks. The 12th International Conference on Distributed Computing in Sensor Systems (DCOSS); , 2016.
7. Muraoka, K.; Watteyne, T.; Accettura, N.; Vilajosana, X.; Pister, K. Simple Distributed Scheduling with Collision Detection in TSCH Networks. *IEEE Sensors Journal* **2016**, *PP*, 1–1. doi:10.1109/JSEN.2016.2572961.
8. Municio, E.; Latré, S. Decentralized Broadcast-based Scheduling for Dense Multi-hop TSCH Networks. Proceedings of the Workshop on Mobility in the Evolving Internet Architecture; ACM: New York, NY, USA, 2016; MobiArch '16, pp. 19–24. doi:10.1145/2980137.2980143.
9. Municio, E.; Spaey, K.; Latré, S. A distributed density optimized scheduling function for IEEE 802.15.4e TSCH networks. *Transactions on Emerging Telecommunications Technologies* **2018**, *29*, e3420, [https://onlinelibrary.wiley.com/doi/pdf/10.1002/ett.3420]. e3420 ett.3420, doi:10.1002/ett.3420.
10. Dujovne, D.; Grieco, L.A.; Palattella, M.R.; Accettura, N. 6TiSCH Experimental Scheduling Function (SFX). Internet-Draft draft-ietf-6tisch-6top-sfx-01, Internet Engineering Task Force, 2018. Work in Progress.
11. Duy, T.P.; Dinh, T.; Kim, Y. Distributed Cell Selection for Scheduling Function in 6TiSCH Networks. *Comput. Stand. Interfaces* **2017**, *53*, 80–88. doi:10.1016/j.csi.2017.03.008.
12. Fahs, A.J.; Bertolini, R.; Alphand, O.; Rousseau, F.; Altisen, K.; Devismes, S. Collision prevention in distributed 6TiSCH networks. 2017 IEEE 13th International Conference on Wireless and Mobile Computing, Networking and Communications (WiMob), 2017, pp. 1–6. doi:10.1109/WiMOB.2017.8115798.
13. Righetti, F.; Vallati, C.; Anastasi, G.; Das, S.K. Analysis and Improvement of the On-The-Fly Bandwidth Reservation Algorithm for 6TiSCH. 2018 IEEE 19th International Symposium on "A World of Wireless, Mobile and Multimedia Networks" (WoWMoM), 2018, pp. 1–9. doi:10.1109/WoWMoM.2018.8449793.
14. Micoli, G.; Boccadoro, P.; Valecce, G.; Petitti, A.; Colella, R.; Milella, A.; Grieco, L.A. ASAP: A Decentralized Slot Reservation Policy for Dynamic 6TiSCH Networks in Industrial IoT. 2019 IEEE International Conference on Communications Workshops (ICC Workshops), 2019, pp. 1–6.
15. van der Lee, T.; Exarchakos, G.; de Groot, S.H. Swarm-Based Energy Efficient Scheduling for Wireless Sensor Networks. 2019 IEEE Conference on Standards for Communications and Networking (CSCN), 2019, pp. 1–6.
16. Domingo-Prieto, M.; Chang, T.; Vilajosana, X.; Watteyne, T. Distributed PID-Based Scheduling for 6TiSCH Networks. *IEEE Communications Letters* **2016**, *20*, 1006–1009. doi:10.1109/LCOMM.2016.2546880.
17. Zhang, Y.; Chen, C.; Zhu, S. An Adaptive Distributed Scheduling Algorithm for IEEE 802.15.4e TSCH Protocol. 2019 3rd International Symposium on Autonomous Systems (ISAS), 2019, pp. 193–198.
18. Theoleyre, F.; Papadopoulos, G.Z. Experimental Validation of a Distributed Self-Configured 6TiSCH with Traffic Isolation in Low Power Lossy Networks. Proceedings of the 19th ACM International Conference on Modeling, Analysis and Simulation of Wireless and Mobile Systems; ACM: New York, NY, USA, 2016; MSWiM '16, pp. 102–110. doi:10.1145/2988287.2989133.
19. Hosni, I.; Théoleyre, F.; Hamdi, N. Localized scheduling for end-to-end delay constrained Low Power Lossy networks with 6TiSCH. 2016 IEEE Symposium on Computers and Communication (ISCC), 2016, pp. 507–512. doi:10.1109/ISCC.2016.7543789.

20. Hosni, I.; Théoleyre, F. Self-healing distributed scheduling for end-to-end delay optimization in multihop wireless networks with 6TiSCH. *Computer Communications* **2017**, *110*, 103 – 119. doi: http://dx.doi.org/10.1016/j.comcom.2017.05.014.
21. Hosni, I. Distributed scheduling with efficient collision detection for end-to-end delay optimization in 6TiSCH multi-hop wireless networks. *Annales des télécommunications* **2019**, *74*, 239–255.
22. Lee, T.H.; Chang, L.H.; Liu, Y.W.; Liaw, J.J.; Chu, H.C. Priority-based scheduling using best channel in 6TiSCH networks. *Cluster Computing* **2017**. doi:10.1007/s10586-017-1185-9.
23. Juc, I.; Alphand, O.; Guizzetti, R.; Favre, M.; Duda, A. Stripe: a Distributed Scheduling Protocol for 802.15.4e TSCH Networks. Research Report RR-LIG-54, Laboratoire d'Informatique de Grenoble, 2017. Les rapports de recherche du LIG - ISSN: 2105-0422.
24. Krueger, L.; Steenbrink, L.; Timm-Giel, A. Avoiding Local Interference in IEEE 802.15.4 TSCH Networks using a Scheduling Function with Distributed Blacklists. Mobile Communication - Technologies and Applications; 24. ITG-Symposium, 2019, pp. 1–6.
25. Yoo, D.; Chung, S.; Ha, Y. Multipath Scheduling for Energy Balancing and Reliable Transmission over 6TiSCH WSN. 2019 Eleventh International Conference on Ubiquitous and Future Networks (ICUFN), 2019, pp. 555–560.
26. Elsts, A.; Pope, J.; Fafoutis, X.; Piechocki, R.; Oikonomou, G. Instant: A TSCH Schedule for Data Collection from Mobile Nodes. Proceedings of the 2019 International Conference on Embedded Wireless Systems and Networks; Junction Publishing: USA, 2019; EWSN '19, pp. 35–46.
27. Hamza, T.; Kaddoum, G. Enhanced Minimal Scheduling Function for IEEE 802.15.4e TSCH Networks. 2019 IEEE Wireless Communications and Networking Conference (WCNC), 2019, pp. 1–6. doi:10.1109/WCNC.2019.8885940.
28. Boucetta, C.; Nour, B.; Moungla, H.; Lahlou, L. An IoT Scheduling and Interference Mitigation Scheme in TSCH Using Latin Rectangles. 2019 IEEE Global Communications Conference (GLOBECOM), 2019, pp. 1–6.
29. Farag, H.; Grimaldi, S.; Gidlund, M.; Österberg, P. REA-6TiSCH: Reliable Emergency-Aware Communication Scheme for 6TiSCH Networks. *IEEE Internet of Things Journal* **2020**, *8*, 1871–1882. doi:10.1109/JIOT.2020.3016643.
30. Vergados, D.J.; Kralevska, K.; Jiang, Y.; Michalas, A. Local voting: A new distributed bandwidth reservation algorithm for 6TiSCH networks. *Computer Networks* **2020**, *180*, 107384. doi: https://doi.org/10.1016/j.comnet.2020.107384.
31. Tavallaie, O.; Taheri, J.; Zomaya, A.Y. Towards Optimizing Time-Slotted Channel Hopping Scheduling on 6TiSCH Networks: Poster Abstract. Proceedings of the 18th Conference on Embedded Networked Sensor Systems; Association for Computing Machinery: New York, NY, USA, 2020; SenSys '20, p. 737–738. doi:10.1145/3384419.3430454.
32. Accettura, N.; Palattella, M.R.; Boggia, G.; Grieco, L.A.; Dohler, M. Decentralized Traffic Aware Scheduling for multi-hop Low power Lossy Networks in the Internet of Things. 2013 IEEE 14th International Symposium on "A World of Wireless, Mobile and Multimedia Networks" (WoWMoM), 2013, pp. 1–6. doi:10.1109/WoWMoM.2013.6583485.
33. Accettura, N.; Vogli, E.; Palattella, M.; Grieco, L.; Boggia, G.; Dohler, M. Decentralized Traffic Aware Scheduling in 6TiSCH Networks: Design and Experimental Evaluation. *Internet of Things Journal, IEEE* **2015**, *2*, 455–470. doi:10.1109/JIOT.2015.2476915.
34. Soua, R.; Minet, P.; Livolant, E. Wave: a Distributed Scheduling Algorithm for Convergecast in IEEE 802.15.4e Networks (Extended Version). Research Report RR-8661, Inria, 2015. Wireless Networks.
35. Soua, R.; Minet, P.; Livolant, E. Wave: a distributed scheduling algorithm for convergecast in IEEE 802.15.4e TSCH networks. *Transactions on Emerging Telecommunications Technologies* **2016**, *27*, 557–575. ett.2991, doi:10.1002/ett.2991.
36. Soua, R.; Minet, P.; Livolant, E. DiSCA: A distributed scheduling for convergecast in multichannel wireless sensor networks. 2015 IFIP/IEEE International Symposium on Integrated Network Management (IM), 2015, pp. 156–164. doi:10.1109/INM.2015.7140288.
37. Aijaz, A.; Raza, U. DeAMON: A Decentralized Adaptive Multi-Hop Scheduling Protocol for 6TiSCH Wireless Networks. *IEEE Sensors Journal* **2017**, *17*, 6825–6836. doi: 10.1109/JSEN.2017.2746183.
38. Daneels, G.; Spinnewyn, B.; Latré, S.; Famaey, J. ReSF: Recurrent Low-Latency Scheduling in IEEE 802.15.4e TSCH networks. *Ad Hoc Networks* **2018**, *69*, 100 – 114. doi: https://doi.org/10.1016/j.adhoc.2017.11.002.

39. Zorbas, D.; Kotsiou, V.; Théoleyre, F.; Papadopoulos, G.Z.; Douligeris, C. LOST: Localized blacklisting aware scheduling algorithm for IEEE 802.15.4-TSCH networks. 2018 Wireless Days (WD), 2018, pp. 110–115. doi:10.1109/WD.2018.8361705.
40. Kim, K.T.; Kim, J. An Energy Efficient Real-Time MAC Protocol. 2018 International Conference on Information and Communication Technology Convergence (ICTC), 2018, pp. 1180–1184.
41. Ünlü, B.; Özceylan, B.; Baykal, B. DIVVY: An Efficient Shared Cell Scheduling Method and Algorithm for 6TiSCH-Based IoT Networks. *IEEE Transactions on Green Communications and Networking* **2019**, *3*, 170–179.
42. Hajian, H.; Nabi, M.; Fakouri, M.; Veisi, F. LaDiS: a Low-Latency Distributed Scheduler for Time-Slotted Channel Hopping Networks. 2019 IEEE Wireless Communications and Networking Conference (WCNC), 2019, pp. 1–7.
43. Jung, J.; Kim, D.; Lee, T.; Kang, J.; Ahn, N.; Yi, Y. Distributed Slot Scheduling for QoS Guarantee over TSCH-based IoT Networks via Adaptive Parameterization. 2020 19th ACM/IEEE International Conference on Information Processing in Sensor Networks (IPSN), 2020, pp. 97–108.
44. Kotsiou, V.; Papadopoulos, G.Z.; Chatzimisios, P.; Theoleyre, F. LDSF: Low-Latency Distributed Scheduling Function for Industrial Internet of Things. *IEEE Internet of Things Journal* **2020**, *7*, 8688–8699. doi:10.1109/JIOT.2020.2995499.
45. Duquennoy, S.; Al Nahas, B.; Landsiedel, O.; Watteyne, T. Orchestra: Robust Mesh Networks Through Autonomously Scheduled TSCH. Proceedings of the 13th ACM Conference on Embedded Networked Sensor Systems; ACM, ACM: New York, NY, USA, 2015; SenSys '15, pp. 337–350. doi:10.1145/2809695.2809714.
46. Oh, S.; Hwang, D.; Kim, K.H.; Kim, K. Escalator: An Autonomous Scheduling Scheme for Convergecast in TSCH. *Sensors* **2018**, *18*. doi:10.3390/s18041209.
47. Kim, S.; Kim, H.; Kim, C. ALICE: Autonomous Link-based Cell Scheduling for TSCH. 2019 18th ACM/IEEE International Conference on Information Processing in Sensor Networks (IPSN), 2019, pp. 121–132.
48. Elsts, A.; Fafoutis, X.; Pope, J.; Oikonomou, G.; Piechocki, R.; Craddock, I. Scheduling High Rate Unpredictable Traffic in IEEE 802.15.4 TSCH Networks. 13th International Conference on Distributed Computing in Sensor Systems (DCOSS). IEEE, 2017.
49. Phung, K.; Huong, T.T.; Khanh Dung, D.; Tuong, V.X.; Pham, T.; Nguyen, T.; Steenhaut, K. A Scheduler for Time Slotted Channel Hopping Networks supporting QoS Differentiated Services. 2018 International Conference on Advanced Technologies for Communications (ATC), 2018, pp. 232–236.
50. Jin, Y.; Raza, U.; Sooriyabandara, M. BOOST: Bringing Opportunistic ROuting and Effortless-Scheduling to TSCH MAC. 2018 IEEE Global Communications Conference (GLOBECOM), 2018, pp. 1–7. doi:10.1109/GLOCOM.2018.8647851.
51. Urke, A.R.; Kure, Ø.; Øvsthus, K. Layered autonomous TSCH scheduler for minimal band occupancy with bounded latency. *Internet Technology Letters* **2020**, *4*, e255, [https://onlinelibrary.wiley.com/doi/pdf/10.1002/itl2.255]. doi:https://doi.org/10.1002/itl2.255.
52. Fafoutis, X.; Elsts, A.; Oikonomou, G.; Piechocki, R.; Craddock, I. Adaptive Static Scheduling in IEEE 802.15.4 TSCH Networks. 2018 IEEE 4rd World Forum on Internet of Things, 2017.
53. Jung, J.; Kim, D.; Hong, J.; Kang, J.; Yi, Y. Parameterized slot scheduling for adaptive and autonomous TSCH networks. IEEE INFOCOM 2018 - IEEE Conference on Computer Communications Workshops (INFOCOM WKSHPS), 2018, pp. 76–81. doi:10.1109/INFCOMW.2018.8407031.
54. Rekik, S.; Baccour, N.; Jmaiel, M.; Drira, K.; Grieco, L.A. Autonomous and traffic-aware scheduling for TSCH networks. *Computer Networks* **2018**, *135*, 201 – 212. doi: https://doi.org/10.1016/j.comnet.2018.02.023.
55. Jeong, S.; Paek, J.; Kim, H.; Bahk, S. TESLA: Traffic-Aware Elastic Slotframe Adjustment in TSCH Networks. *IEEE Access* **2019**, *7*, 130468–130483.
56. Jeong, S.; Kim, H.S.; Paek, J.; Bahk, S. OST: On-Demand TSCH Scheduling with Traffic-Awareness. IEEE INFOCOM 2020 - IEEE Conference on Computer Communications, 2020, pp. 69–78. doi:10.1109/INFOCOM41043.2020.9155496.
57. Gomes, P.H.; Watteyne, T.; Krishnamachari, B. MABO-TSCH: Multihop and blacklist-based optimized time synchronized channel hopping. *Transactions on Emerging Telecommunications Technologies* **2017**, *29*, e3223, [https://onlinelibrary.wiley.com/doi/pdf/10.1002/ett.3223]. e3223 ett.3223, doi:10.1002/ett.3223.

58. Jin, Y.; Kulkarni, P.; Wilcox, J.; Sooriyabandara, M. A centralized scheduling algorithm for IEEE 802.15.4e TSCH based industrial low power wireless networks. 2016 IEEE Wireless Communications and Networking Conference, 2016, pp. 1–6. doi:10.1109/WCNC.2016.7565002.
59. Choi, K.H.; Chung, S.H. A New Centralized Link Scheduling for 6TiSCH Wireless Industrial Networks. Internet of Things, Smart Spaces, and Next Generation Networks and Systems; Galinina, O.; Balandin, S.; Koucheryavy, Y., Eds.; Springer International Publishing: Cham, 2016; pp. 360–371.
60. Choi, K.; Chung, S.H. Enhanced time-slotted channel hopping scheduling with quick setup time for industrial Internet of Things networks. *International Journal of Distributed Sensor Networks* **2017**, *13*, 1550147717713629, [https://doi.org/10.1177/1550147717713629]. doi: 10.1177/1550147717713629.
61. Park, H.; Kim, H.; Kim, K.T.; Kim, S.; Mah, P. Frame-Type-Aware Static Time Slotted Channel Hopping Scheduling Scheme for Large-Scale Smart Metering Networks. *IEEE Access* **2019**, *7*, 2200–2209.
62. Khorov, E.; Lyakhov, A.; Yusupov, R. Scheduling of Dedicated and Shared Links for Fast and Reliable Data Delivery in IEEE 802.15.4 TSCH Networks. 2019 International Conference on Engineering and Telecommunication (EnT), 2019, pp. 1–5. doi:10.1109/EnT47717.2019.9030540.
63. Palattella, M.R.; Accettura, N.; Dohler, M.; Grieco, L.A.; Boggia, G. Traffic Aware Scheduling Algorithm for reliable low-power multi-hop IEEE 802.15.4e networks. 2012 IEEE 23rd International Symposium on Personal, Indoor and Mobile Radio Communications - (PIMRC), 2012, pp. 327–332. doi:10.1109/PIMRC.2012.6362805.
64. Palattella, M.; Accettura, N.; Grieco, L.; Boggia, G.; Dohler, M.; Engel, T. On Optimal Scheduling in Duty-Cycled Industrial IoT Applications Using IEEE802.15.4e TSCH. *Sensors Journal, IEEE* **2013**, *13*, 3655–3666. doi:10.1109/JSEN.2013.2266417.
65. Gaillard, G.; Barthel, D.; Theoleyre, F.; Valois, F. High-reliability scheduling in deterministic wireless multi-hop networks. 2016 IEEE 27th Annual International Symposium on Personal, Indoor, and Mobile Radio Communications (PIMRC), 2016, pp. 1–6. doi:10.1109/PIMRC.2016.7794839.
66. Farias, A.A.; Dujovne, D. A queue-based scheduling algorithm for PCE-enabled Industrial Internet of Things networks. Embedded Systems (CASE), 2015 Sixth Argentine Conference on, 2015, pp. 31–36. doi:10.1109/SASE-CASE.2015.7295844.
67. Soua, R.; Minet, P.; Livolant, E. MODESA: An optimized multichannel slot assignment for raw data convergecast in wireless sensor networks. 2012 IEEE 31st International Performance Computing and Communications Conference (IPCCC), 2012, pp. 91–100. doi: 10.1109/PCCC.2012.6407742.
68. Wu, H.; Lee, D. Robust QoS Scheduling using alternate path for recovery from link failures in IEEE 802.15.4e. 2014 Seventh International Conference on Mobile Computing and Ubiquitous Networking (ICMU), 2014, pp. 99–100. doi:10.1109/ICMU.2014.6799076.
69. Yang, D.; Xu, Y.; Wang, H.; Zheng, T.; Zhang, H.; Zhang, H.; Gidlund, M. Assignment of Segmented Slots Enabling Reliable Real-Time Transmission in Industrial Wireless Sensor Networks. *IEEE Transactions on Industrial Electronics* **2015**, *62*, 3966–3977. doi:10.1109/TIE.2015.2402642.
70. Ramachandran, G.S.; Matthys, N.; Daniels, W.; Joosen, W.; Hughes, D. Building Dynamic and Dependable Component-Based Internet-of-Things Applications with Dawn. 2016 19th International ACM SIGSOFT Symposium on Component-Based Software Engineering (CBSE), 2016, pp. 97–106. doi:10.1109/CBSE.2016.18.
71. Chen, T.S.; Kuo, S.Y.; Kuo, C.H. Scheduling for Data Collection in Multi-hop IEEE 802.15.4e TSCH Networks. 2016 International Conference on Networking and Network Applications (NaNA), 2016, pp. 218–221. doi:10.1109/NaNA.2016.23.
72. Ojo, M.; Giordano, S. An efficient centralized scheduling algorithm in IEEE 802.15.4e TSCH networks. 2016 IEEE Conference on Standards for Communications and Networking (CSCN), 2016, pp. 1–6. doi:10.1109/CSCN.2016.7785164.
73. Ojo, M.; Giordano, S.; Portaluri, G.; Adami, D.; Pagano, M. An energy efficient centralized scheduling scheme in TSCH networks. 2017 IEEE International Conference on Communications Workshops (ICC Workshops), 2017, pp. 570–575. doi:10.1109/ICCW.2017.7962719.
74. Huynh, T.; Theoleyre, F.; Hwang, W.J. On the interest of opportunistic anycast scheduling for wireless low power lossy networks. *Computer Communications* **2017**, *104*, 55 – 66. doi: https://doi.org/10.1016/j.comcom.2016.06.001.
75. Khoufi, I.; Minet, P.; Rmili, B. Scheduling Transmissions with Latency Constraints in an IEEE 802.15.4e TSCH Network. 2017 IEEE 86th Vehicular Technology Conference (VTC-Fall), 2017, pp. 1–7. doi:10.1109/VTCFall.2017.8288164.

76. Matsui, T.; Nishi, H. Time slotted channel hopping scheduling based on the energy consumption of wireless sensor networks. 2018 IEEE 15th International Workshop on Advanced Motion Control (AMC), 2018, pp. 605–610. doi:10.1109/AMC.2019.8371162.
77. Matsui, T.; Nishi, H. ECORS: Energy consumption-oriented route selection for wireless sensor network. 2016 IEEE 14th International Conference on Industrial Informatics (INDIN), 2016, pp. 1044–1049. doi:10.1109/INDIN.2016.7819318.
78. Nsabagwa, M.; Muhumuza, J.; Kasumba, R.; Otim, J.S.; Akol, R. Minimal Idle-Listen Centralized Scheduling in TSCH Wireless Sensor Networks. 2018 41st International Conference on Telecommunications and Signal Processing (TSP), 2018, pp. 1–5. doi:10.1109/TSP.2018.8441425.
79. Minet, P.; Soua, Z.; Khoufi, I. An Adaptive Schedule for TSCH Networks in the Industry 4.0. 2018 IFIP/IEEE International Conference on Performance Evaluation and Modeling in Wired and Wireless Networks (PEMWN), 2018, pp. 1–6.
80. Jin, Y.; Raza, U.; Aijaz, A.; Sooriyabandara, M.; Gormus, S. Content Centric Cross-Layer Scheduling for Industrial IoT Applications Using 6TiSCH. *IEEE Access* **2018**, *6*, 234–244. doi:10.1109/ACCESS.2017.2762079.
81. Devaja, T.; Bajovic, D.; Vukobratovic, D.; Gardaševič, G. Scheduling in 6TiSCH Networks via Max-Product Message-Passing. IEEE EUROCON 2019 -18th International Conference on Smart Technologies, 2019, pp. 1–6.
82. Shi, K.; Zhang, L.; Qi, Z.; Tong, K.; Chen, H. Transmission Scheduling of Periodic Real-Time Traffic in IEEE 802.15. 4e TSCH-Based Industrial Mesh Networks. *Wireless Communications and Mobile Computing* **2019**, *2019*.
83. Brun-Laguna, K.; Minet, P.; Tanaka, Y. Optimized Scheduling for Time-Critical Industrial IoT. 2019 IEEE Global Communications Conference (GLOBECOM), 2019, pp. 1–6. doi:10.1109/GLOBECOM38437.2019.9014218.
84. Harms, O.; Landsiedel, O. MASTER: Long-Term Stable Routing and Scheduling in Low-Power Wireless Networks. 2020 16th International Conference on Distributed Computing in Sensor Systems (DCOSS), 2020, pp. 86–94. doi:10.1109/DCOSS49796.2020.00025.
85. Portaluri, G.; Giordano, S. Gambling on fairness: a fair scheduler for IIoT communications based on the shell game. 2020 IEEE 25th International Workshop on Computer Aided Modeling and Design of Communication Links and Networks (CAMAD), 2020, pp. 1–6. doi:10.1109/CAMAD50429.2020.9209261.
